# Gut microbiome transitions across generations in different ethnicities in an urban setting—the HELIUS study

**DOI:** 10.1186/s40168-023-01488-z

**Published:** 2023-05-08

**Authors:** Eduard W. J. van der Vossen, Mark Davids, Lucas R. F. Bresser, Henrike Galenkamp, Bert-Jan H. van den Born, Aeilko H. Zwinderman, Evgeni Levin, Max Nieuwdorp, Marcus C. de Goffau

**Affiliations:** 1 Department of Experimental Vascular Medicine, Amsterdam UMC Location University of Amsterdam, Meibergdreef 9, Amsterdam, The Netherlands; 2 Horaizon BV, Marshalllaan 2, 2625 GZ Delft, The Netherlands; 3 Department of Public and Occupational Health, Amsterdam UMC Location University of Amsterdam, Meibergdreef 9, Amsterdam, The Netherlands; 4 Department of Vascular Medicine, Amsterdam UMC Location University of Amsterdam, Meibergdreef 9, Amsterdam, The Netherlands; 5 Department of Clinical Epidemiology, Biostatistics and Bioinformatics, Vascular Medicine, Amsterdam UMC Location University of Amsterdam, Meibergdreef 9, Amsterdam, The Netherlands; 6 Sanger Institute, Cambridge, UK

**Keywords:** The HELIUS study, Ethnicity, Migration generation, Gut microbiota, Trophic network, Machine learning

## Abstract

**Background:**

During the course of history, various important lifestyle changes have caused profound transitions of the gut microbiome. These include the introduction of agriculture and animal husbandry, a shift from a nomadic to a more sedentary lifestyle, and recently increased levels of urbanization and a transition towards a more Western lifestyle. The latter is linked with shifts in the gut microbiome that have a reduced fermentative capability and which are commonly associated with diseases of affluence. In this study, in which 5193 subjects are included, we investigated the direction of microbiome shifts that occur in various ethnicities living in Amsterdam by comparing 1st and 2nd generation participants. We furthermore validated part of these findings with a cohort of subjects that moved from rural Thailand to the USA.

**Results:**

The abundance of the *Prevotella* cluster, which includes *P. copri* and the *P. stercorea* trophic network, diminished in the 2nd generation Moroccans and Turks but also in younger Dutch, whilst the Western-associated *Bacteroides/Blautia/Bifidobacterium* (BBB) cluster, which has an inverse correlation with α-diversity, increased. At the same time, the *Christensenellaceae/Methanobrevibacter/Oscillibacter* trophic network, which is positively associated with α-diversity and a healthy BMI, decreased in younger Turks and Dutch. Large compositional shifts were not observed in South-Asian and African Surinamese, in whom the BBB cluster is already dominant in the 1st generation, but ASV-level shifts towards certain species, associated amongst others with obesity, were observed.

**Conclusion:**

The Moroccan and Turkish populations, but also the Dutch population are transitioning towards a less complex and fermentative less capable configuration of the gut microbiota, which includes a higher abundance of the Western-associated BBB cluster. The Surinamese, whom have the highest prevalence of diabetes and other diseases of affluence, are already dominated by the BBB cluster. Given the continuous increase in diseases of affluence, this devolution towards low-diversity and fermentatively less capable gut microbiome compositions in urban environments is a worrying development.

Video Abstract

**Graphical Abstract:**

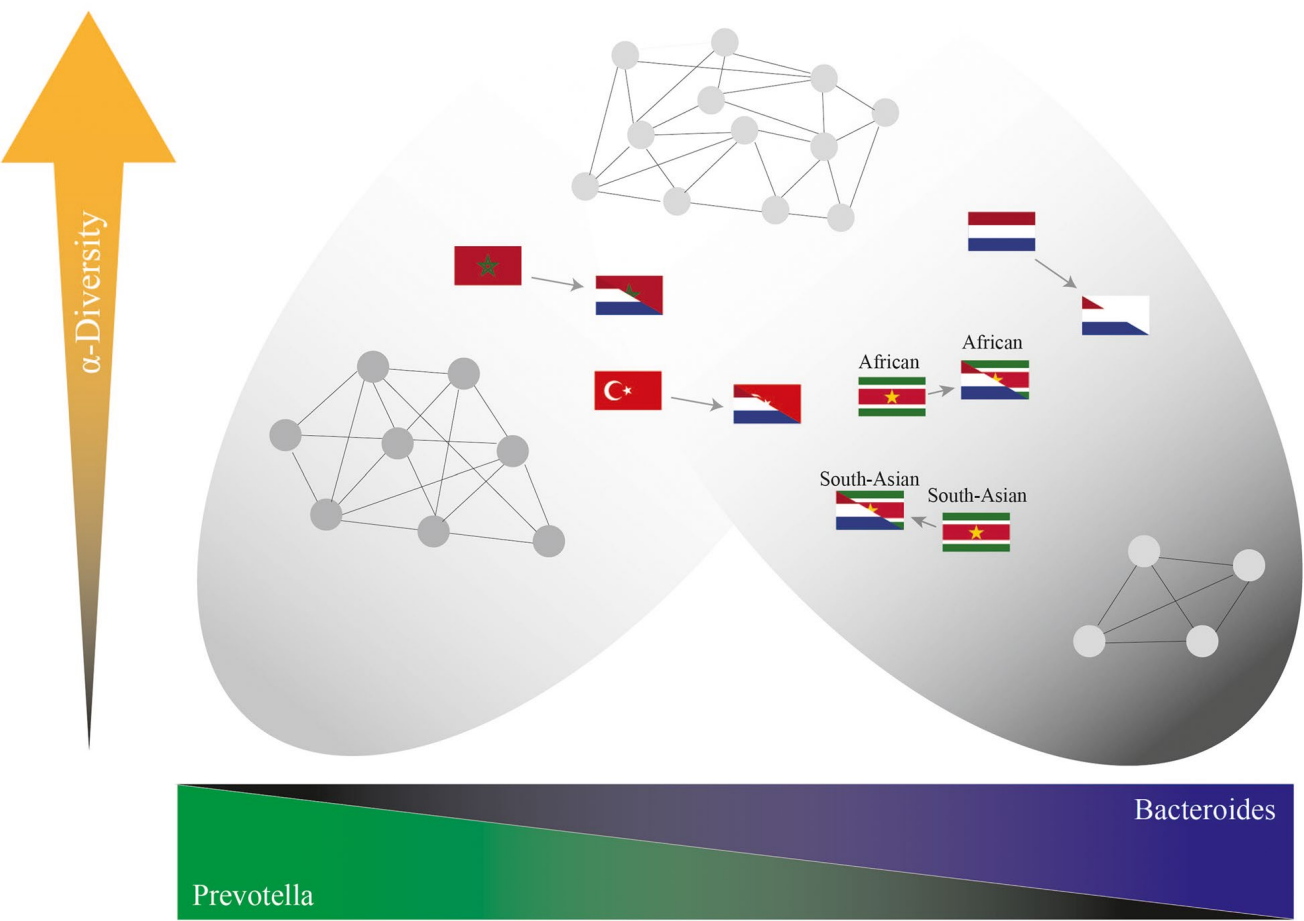

**Supplementary Information:**

The online version contains supplementary material available at 10.1186/s40168-023-01488-z.

## Background

The advent of agriculture and animal husbandry [1, 2], the shift from a nomadic to a sedentary lifestyle [3], urbanization [4, 5], and the (concomitant) shift towards a more Western lifestyle has dramatically affected the microbiome composition in humans [6–8]. The accompanying (d)evolution of the human microbiome has been associated with the rise of diseases of affluence, both inside and outside the gut, immune-mediated, and otherwise [6, 7, 9]. The more Western lifestyle-associated gut microbiome, in particular ones dominated by *Bacteroides*, have been shown both in vitro [10] and in vivo [11] to be fermentatively inferior to (non-Western) *Prevotella* dominated gut microbiomes as short chain fatty acid (SCFA) production levels of acetate, butyrate, and propionate are halved or even less. This difference in fermentative capability is not associated with ethnicity as Americans of African descent typically have the same gut microbiome compositions as Americans of European descent and have similarly “low” SCFA production levels [12]. Studies on the gut microbiota of people, and their descendants, migrating from non-Western societies into Western societies like the US or Europe often show the incremental replacement of *Prevotella* with *Bacteroides* [9, 13]. Interestingly, similar shifts were seen in Irish Travellers forced into a more sedentary urbanized lifestyle [3]. Bacterial composition and functionality are however not just determined by the abundance of a few important genera but by many (phylogenetically) different species, many of whom form collaborative complexes of microbes that interchange metabolites via cross-feeding and syntrophic interactions [14] here called “trophic networks.”

The Healthy Life in an Urban Setting (HELIUS) study is a prospective cohort study that includes nearly 25,000 participants of various ethnicities living in the same geographical location (Amsterdam). In over 5196 participants, V4-16S ribosomal data has been generated. Though the H in HELIUS stands for “healthy,” obesity, diabetes, and depression rates are high but also unevenly distributed amongst the different ethnicities [15]. The continued westernization of the gut microbiome is thought to contribute to these rates as functional fermentative diversity decreases. These shifts can be visualized by comparing the microbiota of the 1st and 2nd generation of ethnic minorities found with the HELIUS cohort, which include Moroccans, Turks, African Surinamese, and South-Asian Surinamese. The 1st generation consists out of immigrants (born outside the Netherlands) and the 2nd generation participants were born in the Netherlands but whose parents were born abroad. In this high-powered study cohort, we tested our hypothesis that a convergence of the microbiota occurs in ethnic minorities, both at a strain- as well as on a trophic-network level, towards a composition more similar to the Dutch origin group. Specific findings were validated using the multi-ethnic, multi-generational cohort of Vangay et al. (USI cohort) [9].

## Methods

### Clinical study inclusion/exclusion criteria and study design

This study was conducted on a subset from the prospective HELIUS study [15]. The aim of the HELIUS study is to investigate the causes of the unequal burden of disease across ethnic groups living in Amsterdam, the Netherlands. Between 2011 and 2015, participants aged 18–70 years were randomly sampled, stratified by ethnic origin, through the municipal registry of Amsterdam, and were sent an invitation letter (and a reminder after 2 weeks) by mail. 55% of those invited were contacted (55% among Dutch, 62% among Surinamese, 57% among Ghanaians, 46% among Turks, and 48% among Moroccans), either by response card or after a home visit by an ethnically matched interviewer. Of those, 50% agreed to participate (60% among Dutch, 51% among Surinamese, 61% among Ghanaians, 41% among Turks, and 43% among Moroccans). Therefore, the overall response rate was 28% with some variations across ethnic groups, resulting in a total of nearly 25,000 participants included at baseline. Subjects from five of the largest ethnic groups included in HELIUS were used in this investigation, including those of Dutch (Northwestern European ancestry), Moroccan (Northern African, Mediterranean, and Middle Eastern ancestries), African Surinamese (“Creoles” descending from former slaves, mixed Western African and Dutch ancestries), South-Asian Surinamese (“Hindustanis” descending from workers arriving to the Suriname post-slavery, South-Asian/Indian ancestry) and Turkish origin (Mediterranean, Caucasian, and Middle Eastern ancestries) [15]. Those of Ghanaian origin were excluded in this study due to the low number of the 2nd generation participants. A person from a non-Dutch ethnic group was considered to be the 1st generation when the person was born outside of the Netherlands and had at least one parent who was born outside the Netherlands. A person from a non-Dutch ethnic group born in the Netherlands of whom both parents were born outside of the Netherlands was considered to be the 2nd generation [16]. A person was considered Dutch and considered for inclusion when he/she was born in the Netherlands and had two parents that were also born in the Netherlands. Participants of the Surinamese ethnic group were further sub-classified according to their self-reported ethnic origin.

### Data collection

Participants filled out a questionnaire on migration-related variables, sociodemographic characteristics, lifestyle, and general quality of life. Information on smoking behavior and alcohol consumption was obtained by questionnaire. Participants also underwent a physical examination at the research location. Weight and height were measured in duplicate in barefoot subjects wearing light clothes only. Body mass index (BMI) was calculated as weight (kg) divided by height squared (m^2^). They were furthermore asked to collect a morning stool sample in the pre-labeled tube and to bring this to the research location within 6 h after collection. The fecal sample was temporarily stored at -20 °C until transportation to the Amsterdam University Medical Center, location AMC. Here, the samples were checked by a study nurse and stored at -80 °C before the analysis of the microbiota composition. In total, 6032 fecal samples were collected and sequenced. After only including the ethnicities with sufficient 2nd generation participants (excluding Ghanaian, Javanese Surinamese, and other Surinamese), 5193 samples remained for analyses.

### Intestinal microbiota—extraction of fecal genomic DNA

Processing of the fecal samples was previously described in Deschasaux et al. [17]. In short, stool samples were shipped to the Wallenberg Laboratory (Gothenburg, Sweden). DNA was extracted from a 150-mg aliquot using the repeated bead beating method, previously described by Salonen et al. [18]. Here, fecal samples were placed in Lysing Matrix E tubes (MP Biomedicals) and extracted twice in lysis buffer (4% w/v SDS; 500 mmol/L NaCl; 50 mmol/L EDTA; 50 mmol/L Tris·HCL; pH 8) with bead beating at 5.5 m/s for 45 s in a FastPrep®-24 Instrument (MP Biomedicals). After each cycle of bead beating, samples were heated at 95 °C for 5 min and then centrifuged at full speed for 5 min at 4 °C. The supernatants from the two extractions were pooled. Six hundred microliters of aliquot from each sample was purified using the QIAamp DNA Mini kit (QIAGEN) in the QIAcube (QIAGEN) instrument using the procedure for human DNA analysis. The samples were eluted in 200 µL of AE buffer (10 mmol/L Tris·Cl; 0.5 mmol/L EDTA; pH 9.0). After the DNA extraction, the 16S rRNA gene was amplified using PCR with the following conditions: initial denaturation for 3 min at 94 °C, followed by 25 cycles of denaturation for 45 s at 94 °C, annealing for 60 s at 52 °C, elongation for 90 s at 72 °C, and a final elongation step for 10 min at 72 °C. Duplicates were combined, purified with the NucleoSpin Gel and PCR Clean-Up kit (Macherey–Nagel), and quantified using the Quant-iT PicoGreen dsDNA kit (Invitrogen). Negative controls were included, and the absence of DNA in these controls was confirmed with gel electrophoresis. Positive controls were not included as the protocol was optimized on mock samples. After PCR, the V4 region of the 16 s rRNA gene was sequenced on a MiSeq system (RTA version 1.17.28, bundled with MCS version 2.5; Illumina) with 515F and 806R primers designed for dual-index sequencing [19] and the MiSeq reagent kit V2 (2 × ​ 250 bp paired-end reads; Illumina). All analytical procedures were blinded for ethnicity (but not randomized).

### Bioinformatic pipeline for gut microbiota profiling

The USEARCH pipeline (v11.0.667) was used to merge, filter, and dereplicate reads. In more detail, paired-end reads were merged (option “fastq_mergepairs”) with a maximum of 100 mismatches (“fastq_maxdiffs”) and at least 70% identity (“fastq_pctid”). Reads were filtered (“fastq_filter”) if the total expected errors based on the Phred (Q) score are larger than 1 (“fastq_maxee”), and hereafter, dereplication was done (“fastx_uniques”) [20]. Next, the UNOISE3 pipeline (“unoise3”) was applied for ASV-level denoising to find the correct biological sequences from the reads [21]. Here, true biological sequence variants were identified, and technical noise and chimeras were removed. Unoise3 denoising was executed at default settings [21]. The final ASV reference database was constructed from ASVs that were inferred in at least one in a thousand samples. ASV abundance was determined per sample (“otutab”). ASV reads with a length lower than 100 bp were omitted. ASV taxonomy was assigned using the RDP classifier and database (v18) [22]. The 300 most abundant ASVs were also individually blasted (Blastn) to confirm their identity and were triple checked by creating a phylogenetic tree to prune out misclassifications. Furthermore, the standard database Nucleotide collection (nr/nt) was applied, excluding uncultured/environmental sample sequences (https://blast.ncbi.nlm.nih.gov/Blast.cgi, Nucleotide Blast). The blast names attached to the ASVs are merely indicative. Many blast results will in time get better matches as the reference library gets expanded or will have their taxonomic designation updated whilst the ASV representative sequence is library and taxonomy independent and should thus be considered leading in regards to all taxonomic designations used in this manuscript and can be used for comparisons with other studies similarly studying the V4 16S region. In order to create a phylogenetic tree, MAFFT (v7.453) [23] was used for multiple sequence alignment using automatically the appropriate setting (“auto”) and FastTreeDbl (v2.1.11) was used to make the phylogenetic tree using the generalized time-reversible model (“GTR”) [24]. To compare subjects to each other, rarefaction was performed at a read depth of 15,221 (using the function “rarefy_even_depth” from R-package “phyloseq” v1.40.1).

### Machine learning analysis

The extreme gradient Boosting (XGBoost) algorithm was utilized to identify a panel of ASVs that best predicted allocation of the migrant generation group within each ethnicity. Thus, for each ethnicity, (Moroccan, Turkish, African and South-Asian Surinamese origin), a model was deployed. Dutch participants were stratified into a young and old age group (≥ 42 years) and were analyzed likewise. Similarly, an age model was also built including all ethnicities with the same age cut-off. ASVs were filtered prior to each simulation to reduce dimensionality. Per model, the top 1000 most abundant ASVs were selected and hereafter, a univariate feature selection was applied based on the ANOVA *F* value to select 100 ASVs used in each simulation. The same stability selection procedure was used in all simulations and all ethnicities to ensure robustness of the results and prevent overfitting [25]. In total, per ethnicity, 20 different subsets were made of the complete dataset. Within each random subset, random under sampling was performed for the 1st generation to have equal group sizes as the 1st generation consisted of more subjects than the 2nd generation. After under sampling, a fractional subset of the under sampled dataset was selected. The fraction was 0.5. Next, within each random subset, LeaveOneOut cross-validation was applied where the training set included all samples except for one, in which this one sample left out was included in the test set. Within the training set, the hyperparameters of XGBoost model were found by performing a randomized search with a three-fold cross-validation, based on 90% of the training set and validated on the remaining 10%. The parameter grid on which the randomized search was applied is given in Table S1, and the number of parameter settings tried was 10. The performance of the different models was estimated via an area under the curve (AUC) of the test dataset to distinguish the 1st from the 2nd generation. The importance of each ASV in the models was extracted and was based on the mean decrease in impurity. This machine learning pipeline was implemented in python (v3.7.7), using the scikit-learn (v0.23.1) package.

### Bacteroides to Prevotella ratio

To assess the *Bacteroides* to *Prevotella* ratio, all *Prevotella*, *Bacteroides*, and *Phocaeicola* (formerly classified as *Bacteroides*) ASVs were identified for this analysis by blasting all Bacteriodales ASVs and verifying their identities by checking their position in a phylogenetic tree (Table S2, Fig. S1).

### Trophic networks

Different (phylogenetically distinct) species represented by various ASVs can be found to cluster together as they might be derived from a particular niche (small intestinal species) or because they represent a network of microbes that thrive together in the same environment (potentially excluding other bacteria) and which together achieve higher rates of growth by means of cross-feeding [14]. This syntrophy between microbes is achieved via chains of conversions of metabolites available in the food web [26]. These trophic networks can often be visualized using the Spearman *ρ* correlation coefficient between ASVs and plotting these in a heatmap, as previously described [27]. Heatmaps were generated by hierarchically clustering using the Euclidean distance of the Spearman *ρ* coefficients. ASVs that are strongly positively correlated with one another form blocks in which all the ASVs tend to be negatively/positively correlated in a similar manner with other competing/synergistic “blocks” of ASVs. These blocks of ASVs (also referred to as clusters) can be considered to represent a trophic network with a degree of confidence if there is either evidence of syntropy (for example the *Christensenellaceae minuta* producing H_2_ which is consumed by *Methanobrevibacter smithii* [28]) or evidence of a co-dependent development over time as can be observed in cohorts of infants during the first 3 years of life (as observed in The Gambia cohort in the case of the *Prevotella stercorea* trophic network which importantly does not include *P. copri* [27]). Furthermore, ASVs within a trophic network should correlate positively with one another and must be found to do so consistently in multiple cohorts/studies. Within this study, we considered ASVs as a core part of a cluster if the ASV was found to be part of a cluster in 6 out of the 10 heatmaps. Here, each heatmap was generated per ethnicity and generation (Tables S3, S4 and S5).

### Statistical analyses

Bray–Curtis dissimilarity between subjects (function “vegdist” of the vegan R-package v2.5.7 [29]) was used to asses interindividual dissimilarity in gut microbiota composition (β-diversity) and was plotted using principal coordinate analysis (PCoA, function “cmdscale” of the stats R-package v4.1.1). Additionally, we applied the generalized UniFrac distance (function “GUniFrac” of the GUnifrac R-package v1.6) [30]. For optimal resolution, ASVs were clustered based on the phylogeny of the sequence. Clustering was done by the agglomeration of tips in the phylogenetic tree at a height of 0.10 (Table S6). This threshold for agglomeration was specifically chosen to represent a genus-like level. A higher height (> 0.10) would for example cluster ASVs of *Faecalibacterium* and *Fournierella* together, which are not only taxonomically but also functionally clearly different. Differences in β-diversity between the different ethnicities and migration generations were assessed using the Permutational Analysis of Variance (PERMANOVA [31]; function “adonis2” from the vegan R-package [29]). The PERMANOVA was applied on the dissimilarity between subjects based on Bray–Curtis dissimilarity of the non-clustered ASVs and the number of permutations was 999.

Comparisons between generations for the log10 (*Bacteroides*/*Prevotella*) ratio, *Faecalibacterium*, and α-diversity of the various ethnicities were assessed using the Mann–Whitney *U* test. The Benjamini–Hochberg method was applied for multiple comparisons [32]. *P* values ≤ 0.05 were considered statistically significant.

The gut microbiota diversity was assessed per individual. Three different metrics were applied, namely the Shannon index, Richness (functions “diversity” and “specnumber” of the vegan R-package v2.5.7 [29], respectively), and Faith’s Phylogenetic Diversity (function “pd” of R-package picante v1.8.2 [33]).

### Validation of our findings using a separate cohort

To validate patterns observed within the HELIUS cohort, we used the publicly available data of Vangay et al. [9]. This cohort consists of subjects living in the rural parts of Thailand, a 1st generation of subjects who moved from Thailand to the USA and a 2nd generation, similarly defined as within our cohort, and European Americans born and living in the USA. The 16s rRNA gene data of the different subjects were obtained from the European Nucleotide Archive under accession number PRJEB28687. This data includes the same V4 region as the HELIUS cohort. Processing of the data from this cohort was done together with the HELIUS cohort using the same pipeline, described above in “Bioinformatic pipeline for gut microbiota profiling.”

### Enterotypes classification

Discretization of subjects in the classical three enterotypes was done as previously described [34]. In short, samples were clustered based on relative genus abundance using the Jensen-Shannon Distance and the partitioning around medoids cluster algorithm. The optimal number for clustering was 3. Stratification of subjects based on their microbiota composition in the form of four enterotypes was established using the Dirichlet Multinomial Mixture approach as previously described [35]. For optimal resolution, ASVs were clustered based on the phylogeny of the sequence (described previously in “Statistical analyses”). Clusters of ASVs were filtered in which the detection limit of a cluster was 0.1%, and the prevalence was at least 50%. The matrix was fed to the Dirichlet Multinomial Mixture Model in which we set the number of components in to 4.

## Results

**Fig. 1:**
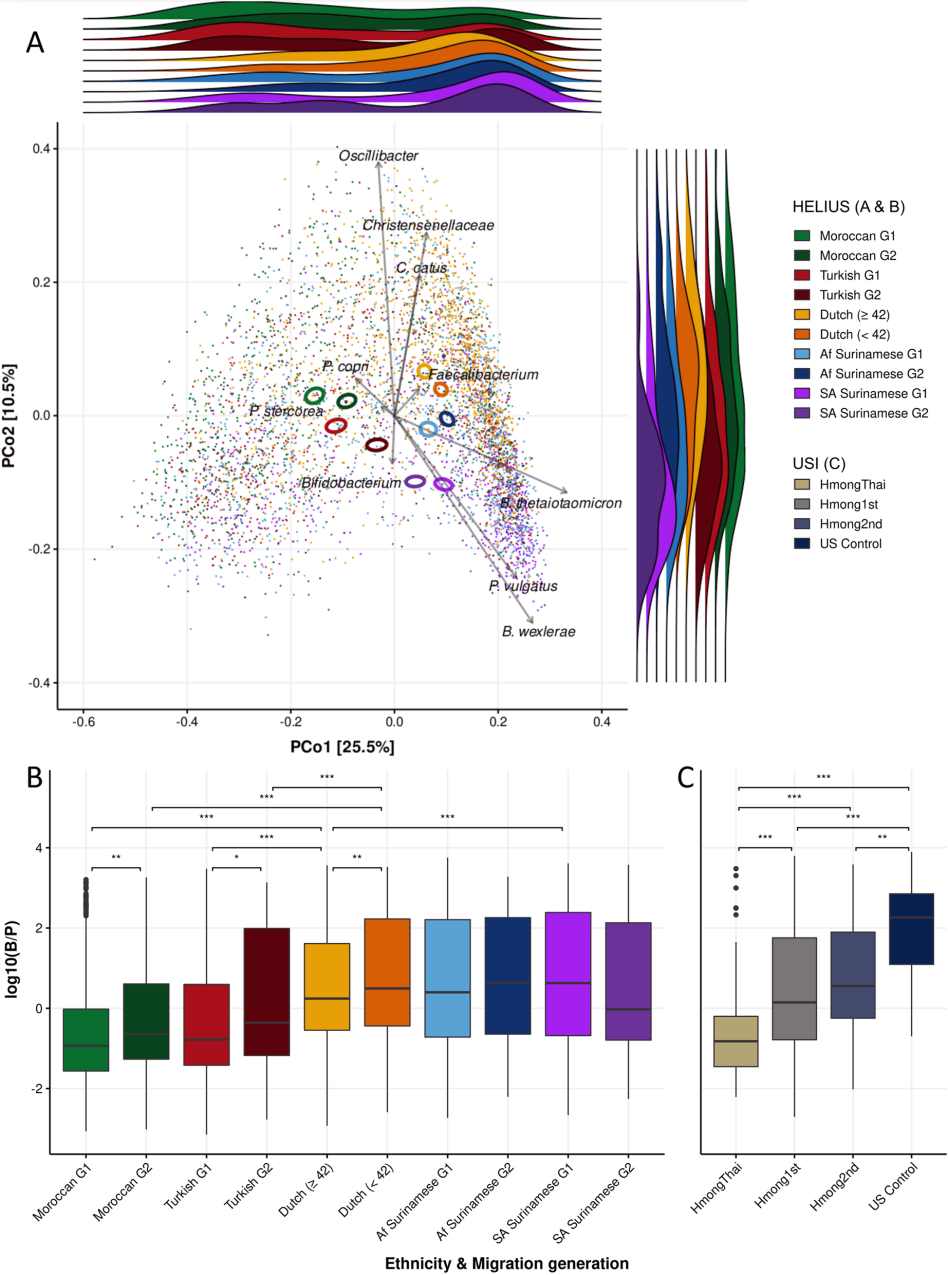
Overview of the gut microbiota of different ethnicities and generations. **A** PCoA plot representing the interindividual differences in the gut microbiota. A ridgeline density plot is positioned on the top and right sides of the PCoA plot, depicting the sample distribution of the different ethnicities and migration generations for each principal coordinate. Scaled loadings of important species are depicted in the different directions. **B**,** C** Log-transformed ratio of *Bacteroides* to *Prevotella* (B/P) abundances per generation and ethnicity for the HELIUS cohort and USI cohort. Significance is based on the Mann–Whitney *U* test (*p value ≤ 0.05; **p value ≤ 0.01; ***p value ≤ 0.001)

A total of 5193 participants from the HELIUS cohort, including 1611 Dutch, 827 Moroccans, 581 Turks, 1421 African Surinamese, and 753 South-Asian Surinamese (Table S7) were analyzed to visualize and understand shifts in the fecal microbiota composition as a result of living in an urban environment by comparing 1st and 2nd generation migrants. Dutch participants were stratified into a young and old age group (≥ 42 years) to mimic the 1st and 2nd generation age difference for comparison purposes to better account for age mediated effects. The USA immigration cohort (USI) by Vangay et al. [9] was used for comparison (see the “Methods” section). The principal coordinate analysis (PCoA) combined with a ridgeline density plot visualizes that each ethnicity and each generation per ethnicity has a different composition distribution (Fig. 1A; PERMANOVA, *R* ^2^ = 0.00292; *p* value ≤ 0.001, Fig. S2 per ethnicity and Fig. S3. Permanova per ethnicity; Moroccan, migration generation, *R* ^2^ = 0.00561; *p* ≤ 0.001, age, *R* ^2^ = 0.00332, *p* = 0.007; Turkish, migration generation, *R* ^2^ = 0.00964; *p* ≤ 0.001, age, *R* ^2^ = 0.00332, *p* = 0.046; Dutch artificial migration generation, *R* ^2^ = 0.006; *p* ≤ 0.001, age, *R* ^2^ = 0.00245, *p* ≤ 0.001; African Surinamese, migration generation, *R* ^2^ = 0.00221; *p* = 0.003, age, *R* ^2^ = 0.00269, *p* ≤ 0.001; South-Asian Surinamese, migration generation, *R* ^2^ = 0.00352; *p* ≤ 0.010, age, *R* ^2^ = 0.00743, *p* ≤ 0.001). Next, we looked at the distance, based on the Bray–Curtis dissimilarity, between the 1st and 2nd generation per ethnicity and the older (≥ 42 years) and younger Dutch, respectively (Fig. S4). The dissimilarity with Dutch became significantly smaller for the 2nd generation in all ethnic minorities when compared to the 1st generation dissimilarity. These results suggest that the gut microbiota composition is changing in the direction of a more Dutch-like gut microbiota composition.

### Bacteroides shift

Enterotyping is commonly employed to stratify subjects based on their microbial composition [34, 36] which reduces the complexity of gut microbiota compositions frequently allowing larger trends to be visualized more easily [36] whilst at the same time circumventing multiple test correction problems. From a birds-eye point of view (enterotypes) of Fig. 1A, it can be said that the Turkish and especially the Moroccan ethnicities are associated with the enterotype driven by *Prevotella* (left side), both Surinamese ethnicities are associated with the enterotype driven by *Bacteroides* (right side), whilst Dutch are associated with the Firmicutes enriched enterotype (top) [36]. A similar analysis including the USI cohort data (Fig. 1C, Fig. S1, Fig. S3B, and Table S2) shows that American controls are *Bacteroides* dominated (Fig. S3B, right side) whilst people in Thailand are *Prevotella* dominated (left side) and that 1st and especially 2nd generation Thai migrants become more similar to Americans (*Bacteroides* dominated). In the HELIUS cohort, a similar shift is observed by looking at the *Bacteroides*/*Prevotella* (B/P) ratio in the *Prevotella*-rich Turkish and Moroccan ethnicities when comparing the 1st with the 2nd generation (Fig. 1B and C, FDR-corrected *p* values of 8.27·10^–3^ and 1.33·10^–2^, respectively). Interestingly, a similar shift towards *Bacteroides* is also seen in the Dutch population (FDR-corrected *p* value of 8.27·10^–3^) but is not observed in the already *Bacteroides*-rich Surinamese populations.

### ASV-level machine learning approach

**Fig. 2:**
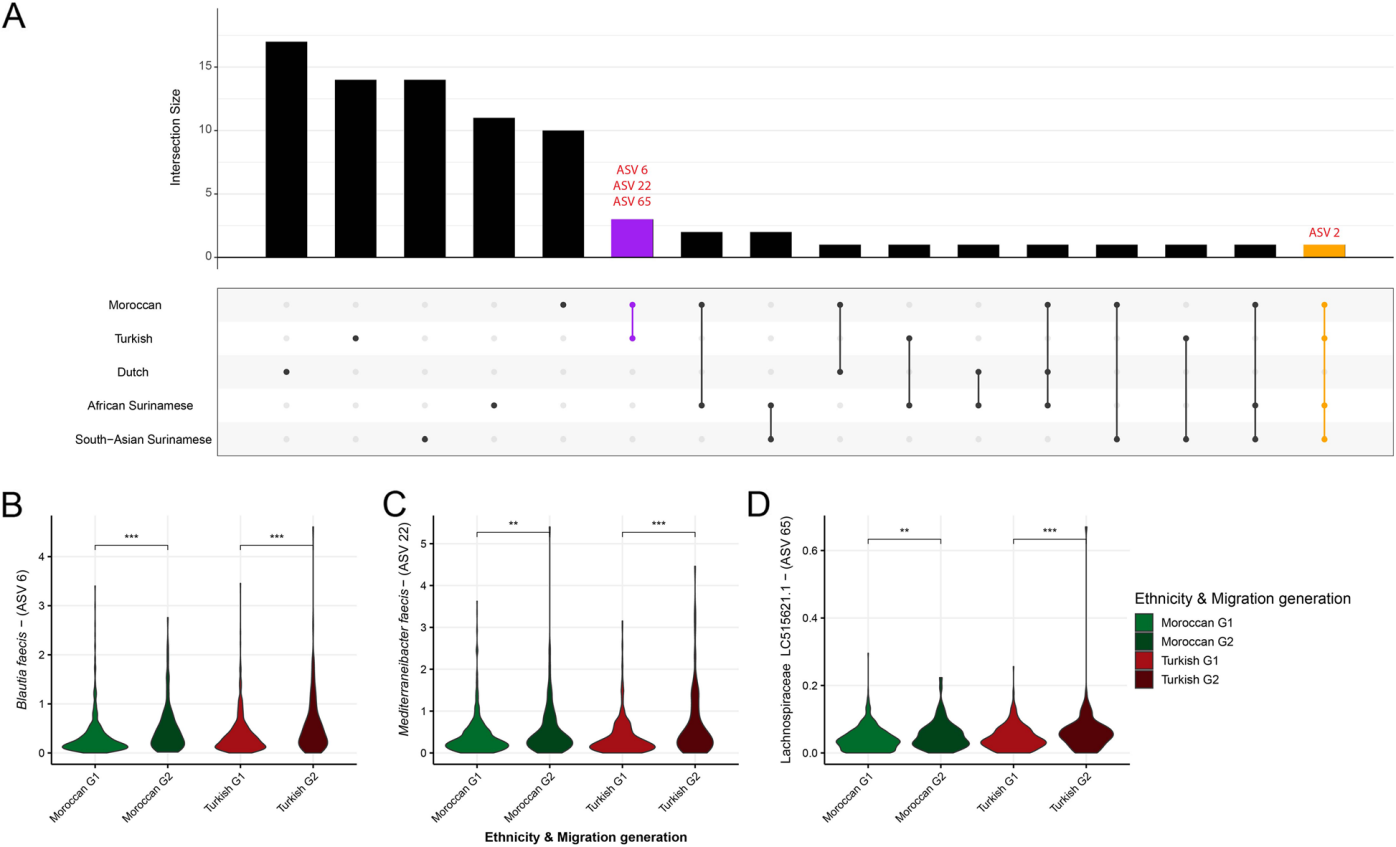
Most discriminative ASVs in each machine learning model distinguishing between the 1st and the 2nd generation. The *y*-axis represents the top 20 most predictive microbial markers. The *x*-axis shows the relative importance of these microbial ASVs normalized between 0 to 100%. Color represents directionality, with blue being higher in the 1st generation and brown/red being higher in the 2nd generation

**Fig. 3:**
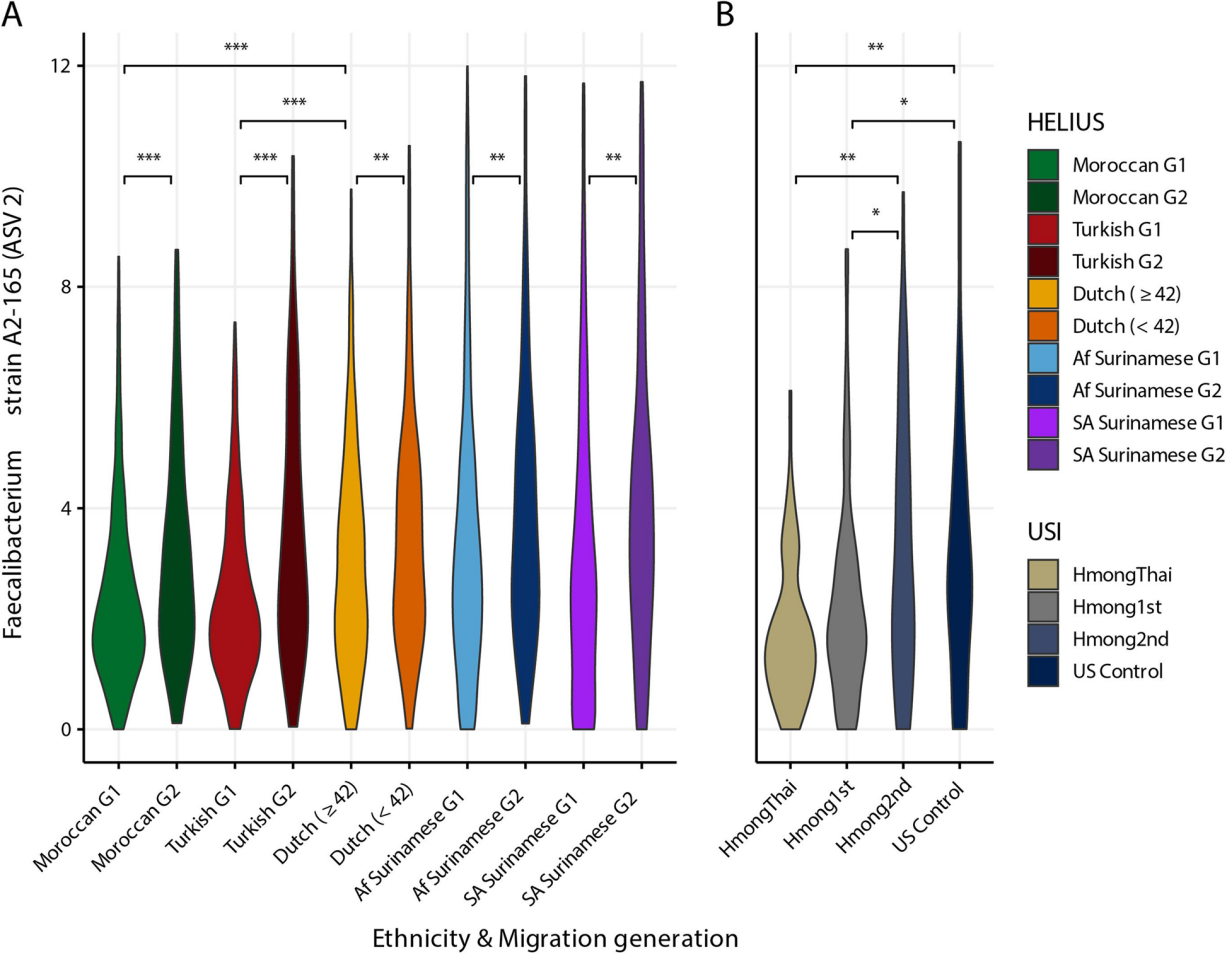
Abundance of the *Faecalibacterium* strain A2-165 (ASV2) in the HELIUS cohort and the USI cohort. Comparison between the 1st and 2nd generation and between the Dutch in the HELIUS cohort (**A**) and comparison between the HmongThai, 1st generation Hmong, 2.nd generation Hmong, and the United States Control group of the USI cohort (**B**). Asterisks denote an FDR-corrected *p* value based on the Mann–Whitney *U* test (*p value ≤ 0.05; **p value ≤ 0.01; ***p value ≤ 0.001)

**Fig. 4:**
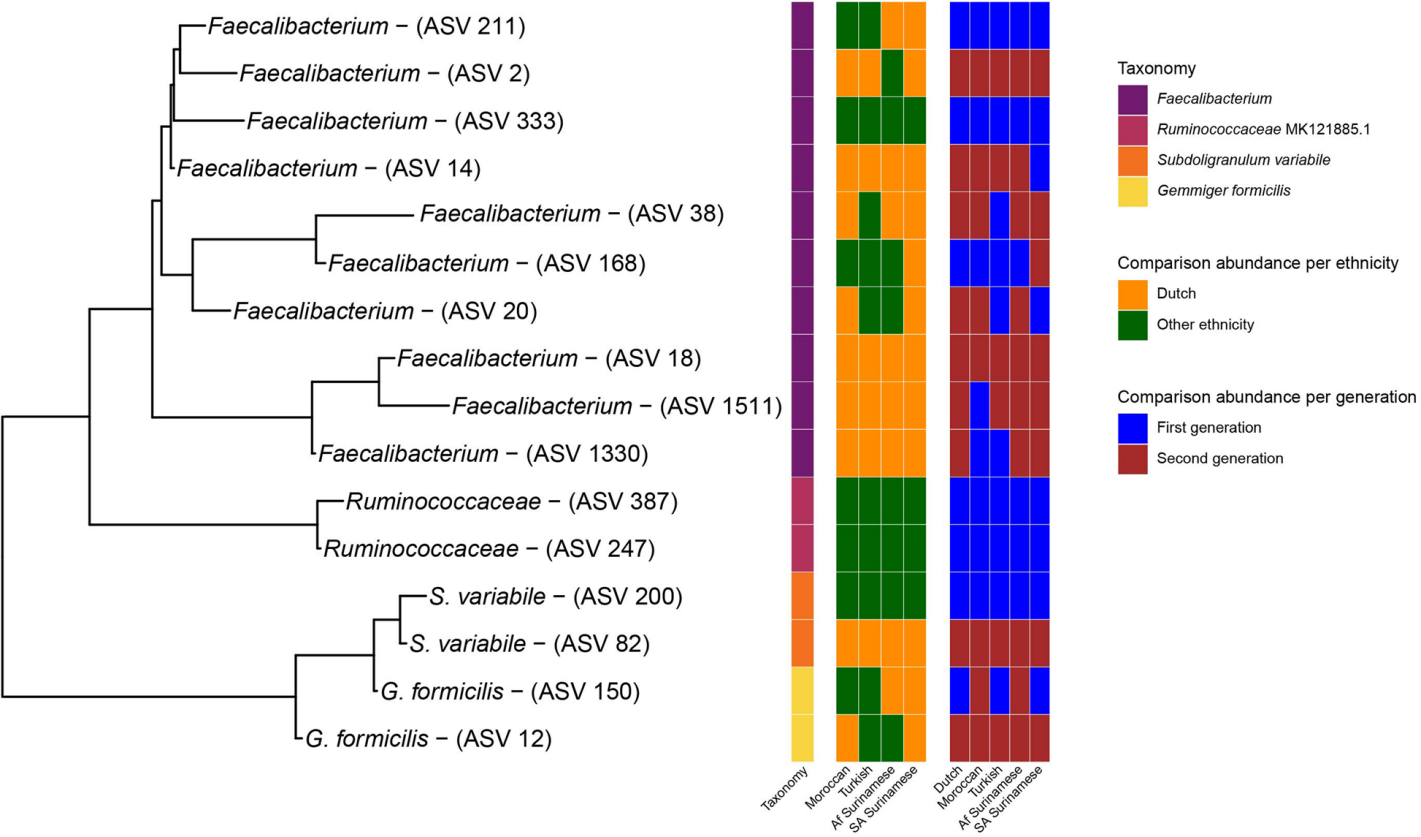
Investigation of different *Faecalibacterium* ASVs and closely related ASVs. Three colored sidebar sections correspond to the tips of the phylogenetic tree. The first sidebar section indicates the different species the ASVs belong to according to a Blastn search. The second indicates whether the ASV has a higher median abundance in the Dutch ethnicity or the other ethnicity and the third indicates whether the ASV has a higher median abundance in the 1st or the 2nd generation per ethnicity

A machine learning approach, the extreme gradient boosting (XGBoost) classification model with LeaveOneOut (see the “Methods” section), was used to distinguish the 1st generation from the 2nd generation per ethnicity on the ASV level (Fig. 2, Table S8). This approach generates a list of features which, according to the model, represent the most predictive ASVs (Fig. S5). *Faecalibacterium* ASV2 (100% identity with strain A2-165) is one of the top machine learning hits in all ethnicities, except in the Dutch. ASV2 is found to be more prevalent in the 2nd generation but also in younger Dutch (Fig. 3A). However, the prevalence of ASV2 rises more rapidly in the 2nd generation in the Turkish and Moroccan population as the significant difference between the Turkish and Moroccan 1st generation with older Dutch disappears when comparing the 2nd generation with the younger Dutch population. This difference was found not to be dependent on the differences in sample size between 1st and 2nd generation. In the USI cohort, the prevalence of ASV2 also rapidly rises towards American-like levels (Fig. 3B). The increase of ASV2 in Surinamese is however similar to the increase seen in Dutch. The area under the curve (AUC; Fig. S6), a measure for the machine learning model to distinguish between the 1st and 2nd generation is indeed larger for the *Prevotella*-rich Turks and Moroccans (0.7 and 0.72, respectively) than it is for African and Asian Surinamese (0.68 and 0.69, respectively). As *Faecalibacterium* is one of the major butyrate producers and is ubiquitous in all humans with an abundance between 5 and 15% [37], we also investigated other *Faecalibacterium* ASVs, and ASVs of species phylogenetically closely related to *Faecalibacterium* such as *Subdoligranulum variabile* and *Gemmiger formicilis*, many of whom were also found to be predictive features (Fig. 2, Table S8). By looking whether the median abundance was higher in the Dutch or in any of the 4 other ethnicities and whether the ASV increased in abundance when comparing the 1st with the 2nd generation a pattern of convergence emerges (Fig. 4). ASVs that overall had a higher median abundance in the Dutch (ASVs 2, 14, 18, 38, & 82) almost universally had a higher median abundance in the 2nd generation whilst the reverse was true for ASVs that were less prevalent in Dutch (ASVs 168, 200, 247, 333, & 387).

### Cluster level shifts

**Fig. 5:**
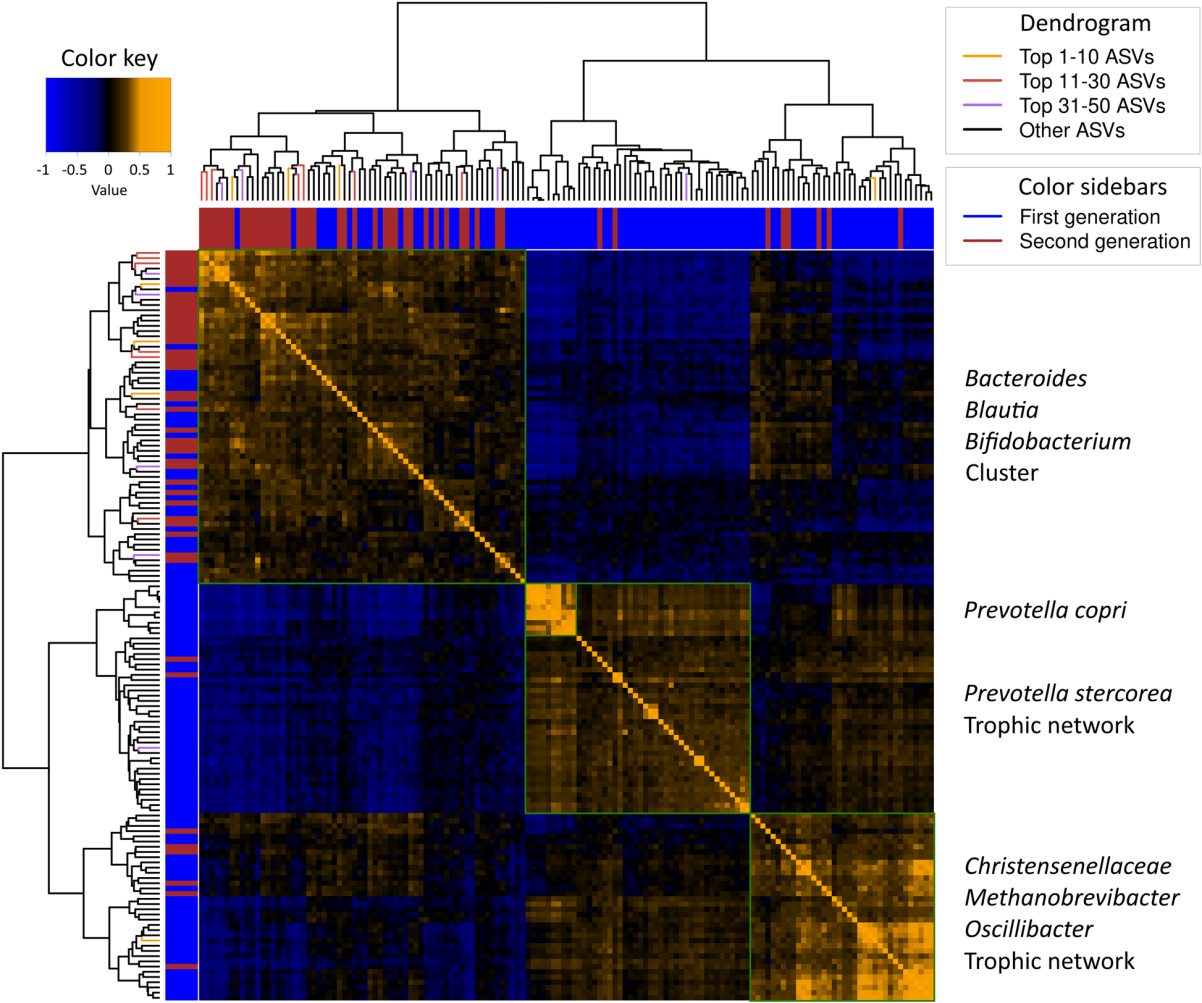
Example heatmap depicting different clusters and/or trophic networks present. Here, the Turkish ethnicity and its machine learning results are included. The colors of the dendrogram include ASVs that were found in the machine learning model distinguishing the 1st from the 2nd migration generation. The colors of the sidebars depict whether the median abundance of the ASV is higher in the 1st or 2.nd generation. The heatmap itself consists of the spearman *ρ* correlation values of the ASVs, which are due to hierarchical clustering split up in different blocks consisting of clusters of ASVs. Based on previous analyses, the central *Prevotella* cluster can be divided into the *P. copri* and *P. stercorea* trophic network [27]. The *Christensenellaceae*, *Methanobrevibacter*, *and Oscillibacter* cluster is also considered a trophic network based on cross-feeding interaction knowledge [28, 38]

Several other predictive features derived from machine learning are also representative of larger patterns of displacement which are however more easily visualized within the context of clusters or trophic networks. Visualizing such clusters using heatmaps in which the correlations of the top 200 most abundant ASVs per ethnicity per generation are sorted using hierarchical clustering allows one to define the most reproducible core set of ASVs which together are representative of each cluster or trophic network (Fig. 5, Fig. S9, and Table S9). Moreover, clustering and heatmap visualization allows us to seamlessly integrate the results of a state-of-the-art machine learning model which was applied to identify a panel of ASVs for each ethnicity to find leads that distinguish the 1st from the 2nd generation. The first cluster is centered around but not limited to *Bacteroides*, *Blautia*, and *Bifidobacterium* (BBB). The second cluster is centered around, but not limited to, the *Prevotella* genus (P) and is accompanied by a list of phylogenetically diverse species. The third cluster is centered around *Christensenellaceae*, *Methanobrevibacter*, and *Oscillibacter* (CMO). Whilst large differences exist between the 5 different ethnicities and between generations these three clusters can be recognized reproducibly. ASVs that were found to be present within a cluster in the majority of heatmaps generated (6/10) were flagged as core ASVs and used for further statistical analyses (Tables S3, S4 and S5). These clusters are coherent with the classical three enterotype division [34] and to a lesser extent to the four enterotype division (Figs. S7C and S8C, respectively). Other ASVs often form their own small clusters such as small intestinal bacteria, metformin sensitive bacteria (diabetes medication), or bacteria commonly associated with dysbiosis such as *Enterobacteriaceae* and *Enterococcus*.

Heatmaps in the Turkish, Moroccan, and Dutch groups show that nearly all ASVs of the *Prevotella* cluster go down in the 2nd generation and that the same is largely true for CMO. Most ASVs of the BBB cluster on the other hand, with which the *Prevotella* and many ASVs of the CMO cluster are both negatively correlated with, increase. Many of the top machine learning features are concentrated in the BBB cluster. Analyses of the sum of all core ASVs of each cluster, as defined above, show that significant shifts occur within the Turkish, Moroccan, and Dutch populations but not in the Surinamese (Table 1).

**Table 1:** Mean abundance of different clusters and trophic networks per ethnicity and their Spearman *ρ* correlations with BMI, triglycerides, and α-diversity (Shannon index)

**Cluster/tropic network**	**Ethnicity**	**Mean abundance (%)**		**BMI**	**Triglycerides**	**Shannon index**
		1st gen/ old Dutch	2nd gen/young Dutch			
***P. copri***	Moroccan	14	12.2	0.14(***)	0.067(.)	− 0.4(***)
**Sub-cluster**	Turkish	14.6	13	0.13(**)	0.1(*)	− 0.39(***)
	Dutch	5.7	5.7	0.019	0.045(.)	− 0.045(.)
	African Surinamese	7.2	5.7	0.022	0.013	− 0.053(*)
	South-Asian Surinamese	7.7	11.1	− 0.022	0.0054	− 0.016
***P. stercorea***	Moroccan	11.2(.)	9.6(.)	0.11(**)	0.092(**)	− 0.025
**Trophic network**	Turkish	8.6(**)	6.6(**)	0.12(**)	0.13(**)	0.011
	Dutch	4.4(***)	3.2(***)	0.12(***)	0.028	0.16(***)
	African Surinamese	5.8	4.6	0.05(.)	0.0013	0.14(***)
	South-Asian Surinamese	5.9	5.5	− 0.00031	0.00079	0.26(***)
**CMO**	Moroccan	7.3	7.3	− 0.067(.)	− 0.12(***)	0.72(***)
**Trophic network**	Turkish	6.0(*)	5.0(*)	− 0.13(**)	− 0.087(*)	0.72(***)
	Dutch	8.7(*)	7.8(*)	− 0.16(***)	− 0.11(***)	0.7(***)
	African Surinamese	5.9	6.5	− 0.028	− 0.11(***)	0.75(***)
	South-Asian Surinamese	3.2	3.2	− 0.033	− 0.11(**)	0.75(***)
**BBB**	Moroccan	10.5(***)	12.8(***)	− 0.14(***)	− 0.012	− 0.091(**)
**Cluster**	Turkish	13.4(***)	18.0(***)	− 0.11(**)	− 0.081(.)	− 0.075(.)
	Dutch	14.9(***)	17.5(***)	0.067(**)	0.03	− 0.37(***)
	African Surinamese	19	19.8	− 0.021	0.017	− 0.31(***)
	South-Asian Surinamese	22.5	21.4	− 0.022	0.024	− 0.28(***)

Asterisks denote the following significant *p* values: (.) ≤ 0.1; (*) ≤ 0.05; (**) ≤ 0.01; (***) ≤ 0.001

### Trophic networks, α-diversity, and health

**Fig. 6:**
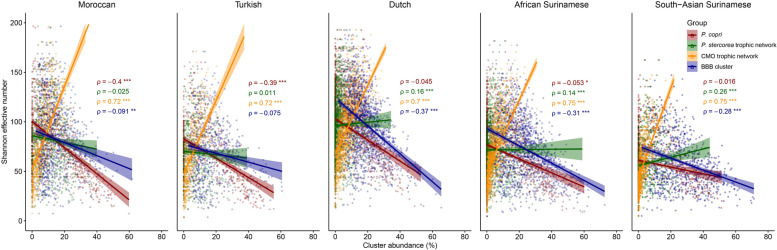
Analysis of Spearman *ρ* correlations between the abundance of different clusters and α-diversity (Shannon effective number). The *Prevotella* cluster is split up into the *P. copri* cluster and the *P. stercorea* trophic network (*p value ≤ 0.05, **p value ≤ 0.01, ***p value ≤ 0.001)

Higher α-diversity values are often used as a proxy for good health. A high α-diversity is typically an indication of the presence of extended and well-developed trophic networks with high fermentative capacity (Fig. 6). The CMO cluster is representative one of the most visually distinct and coherent yet still underappreciated trophic networks in regards to health [39]. This trophic network is most abundant in the Dutch population yet is not found to increase in abundance in any of the ethnicities in the 2nd generation as compared to the 1st generation; it even decreases significantly in the Turkish 2nd generation and younger Dutch as compared to older Dutch. In the Dutch population (and others), age is strongly associated with BMI (*ρ* = 0.31, *p* = 2.2·10^–16^) and while age is positively correlated with the CMO network (*ρ* = 0.066, *p* = 7.56·10^–3^), the abundance of CMO is more strongly negatively associated with BMI (*ρ* =  − 0.16, *p* = 1.00·10^–10^). Similarly, all ASVs from the *Prevotella* cluster, except the ones of *Prevotella copri*, represent a complex trophic previously found to be centered around *P. stercorea* [27] and are similarly positively associated with α-diversity in most ethnicities, unlike the BBB cluster or *P. copri* for which the reverse is true (Fig. 6 and Table 1). As a logical consequence, α-diversity analyses show a slight decrease in the 2nd generation Moroccans compared to the 1st generation, but a significant decrease in the Turkish and Dutch population (Fig. S10). This is concomitant with the significant decrease and increase of the CMO and BBB clusters, respectively, in these two populations. Both of the 2nd generation Surinamese populations do not show a decrease in α-diversity.

The HELIUS cohort unfortunately lacks data on transit times and stool consistency. Nonetheless, in accordance with Vandeputte et al. (2016) and Falony et al. (2016), who observed an association between looser stool samples and the *Prevotella* enterotype, and with the fact that women more commonly suffer from constipation, have longer transit times and score slightly lower on the Bristol stool scale score (harder stool) [40, 41], we observe that the BBB cluster and CMO trophic networks tend to on average have higher abundances in females and that abundances of *P. copri* and the *P. stercorea* trophic network are higher in males (Fig. S11).

## Discussion

The gut microbiome shift associated with a transition from a hunter-gatherer lifestyle towards one associated with agriculture has occurred in most populations around the world. Yet, a (continued) loss of functional microbial diversity and a convergence towards an atrophied composition associated with diseases of affluence is an ongoing process worldwide as people undergo additional physical and/or cultural shifts towards a more industrialized and urbanized settled setting [1, 3, 9]. We investigated this process making use of the large multi-ethnic HELIUS cohort comparing gut microbiota shifts between 1st and 2nd generation migrants from different ethnic minorities recently settled in Amsterdam whilst at the same time comparing these shifts with developments observed in the Dutch of similar age. Our initial hypothesis of a simple convergence within these ethnic minorities towards a more Dutch-like composition was found to be only mostly valid on a strain/ASV-based level yet was partially flawed on a higher more compositional level as the Dutch themselves as a whole are found to still be in transition.

On an ASV-level, both β-diversity analyses and machine learning showed a convergence towards a more Dutch-like composition. Different ASVs of *Faecalibacterium*, a ubiquitous hominid commensal [37, 42], were found to be important discriminative features in our machine learning approach and similarly shows a preference for strains in the 2nd generation that are more common in the Dutch population (Fig. 4, Table S8A). The B/P ratio, a common metric for looking at the gut microbiota at a more compositional level in regards to westernization of the gut microbiome [9, 43], however gives a mixed yet logical result. The B/P ratio increases strongly in both 2nd generation Turks and Moroccans who both start off with a low B/P ratio (1st generation), similarly to the Thai immigrants in the USI cohort. A smaller yet still significant increase of this ratio is similarly seen when comparing younger (< 42 years) with older Dutch yet no shift is seen in this ratio in both Surinamese groups. Older Dutch but especially the Surinamese have high B/P ratios to begin with. This could perhaps partially be explained by the traditional Surinamese diet, as Surinamese individuals tend to adhere to a dietary pattern that is characterized by traditional Surinamese foods, importantly including sugar-sweetened beverages [44]. Saccharolysis is strongly linked with the *Bacteroides* (2) enterotype [45]. *Bacteroides* and *Prevotella* compete for the same niche in the gastrointestinal tract [46] and are some of the most discriminating genera for describing the gut microbiota composition, as the enterotype discussion attests to [36]. Higher *Bacteroides* abundances are commonly associated with diabetes while the inverse is true for *Prevotella* [47–49] which is found to have a protective function against *Bacteroides*-induced glucose intolerance [46]. In the HELIUS cohort, diabetes and metabolic syndrome prevalence are indeed by far the highest in Surinamese, particularly South-Asian Surinamese (Table S7) [50]. This is highlighted even further using a 4-enterotype gut microbiota constellation based on the Dirichlet Multinomial Mixture model, as a large part of the South-Asian Surinamese are of the *Bacteroides*2 enterotype composition, which is commonly associated with a low-cell count, low α-diversity, dysbiosis, and the aforementioned diseases [51–53].

In this study, the large number of subjects however enabled us to employ the concept of clustering ASVs in order to visualize complex trophic networks as an alternative to showing large ecological shifts in the microbial composition [27, 54]. Heatmaps readily visualize the shift from *Prevotella* towards *Bacteroides* in Moroccans, Turks, and Dutch as most ASVs in the BBB cluster increase in the 2nd generation whilst most ASVs which are part of the *Prevotella* cluster decrease. In addition to this, a significant shift away from the CMO network in the younger groups is similarly observed in Turks and Dutch and to a limited degree in Moroccans.

In contrast, no significant directional shifts are observed in the Surinamese ethnicities, whose microbiota are already BBB rich in the 1st generation. Machine learning results do however suggest that some of these rearrangements could be associated with an increase of species associated with increased obesity. ASVs from the *Dorea* genus for example, which has been associated with obesity [55–58], increased in the 2nd generation Surinamese (Fig. 2).

A common observation when distinguishing healthy from unhealthy subjects within cohorts is a higher α-diversity [9, 59–62]. The CMO network is strongly positively correlated with α-diversity and leanness (Fig. 6 and Table 1). The syntrophic relationship between *Christensenellaceae* and *Methanobrevibacter*, indicator species of this cluster, is well described as the Archaea *Methanobrevibacter* consumes the hydrogen produced by the *Christensenellaceae* bacteria converting it into methane [28]. Both species have been reported to be associated with reduced obesity and BMI [39, 63], and the positive association between both is a robust feature of microbiome studies worldwide [39]. Other ASVs however, of often poorly characterized species, show a similarly strong correlation with one another in this network as the correlation between *Christensenellaceae* and *Methanobrevibacter*, suggesting that they are of equal biological importance and are part of a larger coherent cross-feeding network. Age is often found to be associated with this trophic network [39], but this could also indicate a slowly (partially) replacement by the BBB cluster in an industrialized urban setting. The study by Keohane et al. in Ireland with Traveler communities who were forced to end their nomadic lifestyle seems to indicate that the decline of the CMO network is not due to age but due to lifestyle factors [3]. Given the robust association of this network as a whole with BMI (and other diseases of affluence), investigation of the less well characterized members of the CMO network is of prime importance to human health.

Contrary to the CMO network, the BBB cluster has a strong inverse correlation with α-diversity. Most subjects who have a high abundance of this cluster are positioned in the *Bacteroides* enterotype; CMO rich subjects are typically classified as having a Firmicutes-enriched enterotype. The *Bacteroides* enterotype is considered a risk factor for diabetes [52, 53], one of the many diseases of affluence in the Western population. Interestingly, the BBB cluster is negatively associated with BMI in the Moroccan and Turkish ethnicities, whereas the *Prevotella* network is positively correlated with BMI in these ethnicities. A similar observation was also made by Kaplan et al. investigating the Hispanic community living in the USA [43]. Here, a higher B/P ratio was inversely correlated to obesity. Whilst Kaplan et al. found a negative association of the *Prevotella* enterotype with diversity, we observed a partial positive correlation between the *Prevotella* cluster and α-diversity; the *Prevotella* cluster at low abundances is positively correlated with α-diversity whilst this association becomes negative at high abundance (Fig. 6). The reason for this is that the *Prevotella* cluster, or the *Prevotella* enterotype for that matter, should not be seen as a monolithic entity but as a combination of (1) a complex trophic network of various species (*P. stercorea*, *Catenibacterium mitsuokai*, *Holdemanella biformis*, *Phascolarctobacterium succinatutens*, *Mitsuokella*, and various others) that builds up relatively slowly over time; as shown in a cohort of children aged ½–3½ from The Gambia [27] and (2) *P. copri* which becomes dominant rapidly after weaning within the first year of life, independent of any other strains. *P. copri*, a bacterium which does not appear to rely on others, is strongly inversely correlated with α-diversity in *Prevotella*-rich populations such as the Moroccans and Turks (Fig. 6). In the study by Kaplan et al., Hispanics mainly had high *P. copri* numbers explaining the negative correlation with α-diversity [43]. In the South-Asian Surinamese population, we saw a decrease of all ASVs of the *Prevotella* cluster in the 2nd generation except for an enrichment of *P. copri* (ASV46) which was also observed to be an important discriminatory feature in the machine learning model. As *P. copri* is independent of other strains, it is relatively straightforward to obtain and maintain this bacterium in the gastrointestinal tract. Typically, the acquisition of *Prevotella* is linked with an increased intake of dietary fibers and complex carbohydrates and is hence more frequently found dominant in vegans [64]. Interestingly, whether *P. copri* should be considered beneficial or disadvantageous remains unclear or is situational. There is an association with improved glucose tolerance [46], but also with obesity [43, 65]. The complex *Prevotella* trophic network component described above is however likely more difficult to obtain and maintain. It was shown for example in mice that the progressive loss of microbial species over several generations due to lack of such complex carbohydrates in their diet was not recoverable by reintroducing these carbohydrates, but required fecal microbial transplantation [66].

Limitations of this study include that accounting for the effects of diet remains a challenge as some shifts are affected by diet but also by age, sex, and/or the overall dominant microbiota composition, such as the *Bifidobacterium* genus, or are indirectly affected due to higher medication use (metformin especially) in the 1st generation such as *Romboutsia ilealis*, other *Peptostreptococcaceae* and *Clostridium celatum*. There is furthermore undoubtedly a tendency in part of the younger generation to eat a rather unhealthy and unbalanced diet; this is an ongoing socioeconomic/cultural shift that is however not linked with age directly. Furthermore, HELIUS is multi-generational multiethnic cohort with the aim to represent the typical population of Amsterdam, which entails that a multitude of subjects have various ailments. No HELIUS subjects were however excluded in this analysis for medical reasons instead relying on the power of large numbers of subjects to visualize overarching population-wide transitions within each of the different ethnicities.

## Conclusions

The main transition observed within the Moroccan and Turkish but also in the Dutch population is one towards a composition with a higher abundance of the more Western-associated BBB cluster. The non-Western *Prevotella* cluster declines in the 2nd generation, and the same is largely true for the CMO network. Surinamese, in whom rates of diseases of affluence are highest and whose gut microbiota composition generally have a low α-diversity and are already dominated by BBB, similar to Americans, mainly show ASV-level shifts. It is also known that α-diversity decreases at old age but this decline is not observed in elderly reaching extremely high ages where *Christensenellaceae* and *Methanobrevibacter* are found to be enriched compared to all other groups [67–69]. The disappearance of complex trophic networks associated with the *Prevotella* and the CMO network, which can be directly linked to a reduction in α-diversity in the younger generation, does not bode well from a health perspective for both immigrants and locals living in an urbanized environment.

## Supplementary Information

**Additional file 1: Table S1.** Parameter grid used for tuning of the XGBoost model. **Table S2.***Prevotella*, *Bacteroides* and *Phocaeicola* ASVs based on BLAST search. Note that *Phocaeicola *is included as it was previously categorized as *Bacteroides*. **Table S3.** Identification of the core ASVs in the CMO trophic network. The names for the ASVs were based on a BLAST search. **Table S4.** Identification of the core ASVs in the *Prevotella *cluster. The names for the ASVs were based on a BLAST search. **Table S5.** Identification of the core ASVs in the BBB cluster. The names for the ASVs were based on a BLAST search. **Table S6.** Clustering of ASV sequences based on phylogeny at a height of 0.1. **Table S7.** Summary of demographics of HELIUS subjects included in study. **Table S8.** Most discriminative ASVs found in machine learning that distinguish the 1^st^ from the 2^nd^ generation per ethnicity including the Dutch with the ranking of the importance (A), an age-only model with the overlap to the ethnicities (B) and the train-, cross-validation-, and test ROC-AUC scores with standard deviation for each model (C). **Table S9.** ASVs that were presented in the heatmap of the Turkish ethnicity (Fig. 5; Fig. S9). The ASV names were based on a BLAST search.

**Additional file 2: Fig. S1. ***Bacteroides*,* Phocaeicola* (formerly also classified as *Bacteroides*) and *Prevotella* ASV selection based on phylogeny.

**Additional file 3: Fig. S2.** PCoA plot representing the interindividual differences in the gut microbiota of the different ethnicities. PERMANOVA is based on the Bray-Curtis distance on each ethnicity. Formula used: Bray-curtis ~ Migration generation + age. The results for (A) Moroccan, migration generation *R2* = 0.00561; *p*≤0.001, age *R2* = 0.00332, *p*=0.007; (B) Turkish, migration generation *R2* = 0. 00964; *p*≤0.001, age *R2* = 0.00332, *p*=0.046; (C) Dutch artificial migration generation *R2* = 0. 006; *p*≤0.001, age *R2* = 0.00245, *p*≤0.001; (D) African Surinamese, migration generation *R2* = 0. 00221; *p*=0.003, age *R2* = 0.00269, *p*≤0.001; (E) South-Asian Surinamese, migration generation *R2* = 0.00352; *p*≤0.010, age *R2* = 0.00743, *p*≤0.001.

**Additional file 4: Fig. S3.** PcoA based on the Generalized UniFrac for the HELIUS (A) and USI (B) cohorts.

**Additional file 5: Fig. S4.** Mean Bray-Curtis dissimilarity compared to the Dutch. Here, the 1^st^ generation of each ethnicity was compared to the older aged Dutch (mean age of 57.2) and the 2^nd^ generation to the younger aged Dutch (mean age of 32.4) to correct for age confounding effects. Significance is based on the Mann-Whitney U test (asterisks *p-value ≤0.05; **pvalue ≤0.01; ***p-value ≤0.001).

**Additional file 6: Fig. S5.** Boxplots of the top 20 ASVs found in the different machine learning models of the Moroccan (A), Turkish (B), Dutch (C), African Surinamese (D), South-Asian Surinamese (E), and an age model with all ethnicities (F). Significance is based on the FDR-corrected Mann-Whitney U test (asterisks * *p*-value ≤0.05; **pvalue ≤0.01; ***p-value ≤0.001).

**Additional file 7: Fig. S6.** ROC-AUC scores of the different machine learning simulations. The different ethnicities are Moroccan (A), Turkish (B), Dutch (C) African Surinamese (D), South-Asian Surinamese (E). Lastly, an age-only model based on all ethnicities at a cut-off of 42 years old was applied (F).

**Additional file 8: Fig. S7.** Classical three enterotype division described by Arumugam et al. [34] of the HELIUS cohort including a PCoA plot based on the Bray-Curtis dissimilarity (A), a stacked bar chart stratified by ethnicity and migration generation (B), and the relative abundance of the different clusters stratified by the classical three enterotyping (C). *P* = Prevotella, CMO = *Christensenellaceae/Methanobrevibacter/Oscillibacter* and BBB = *Bacteroides/Blautia/Bifidobacterium*.

**Additional file 9: Fig. S8.** Four enterotype division based on the Dirichlet Multinomial Mixture model described by Holmes et al. [35] of the HELIUS cohort including a PCoA plot based on the Bray-Curtis dissimilarity (A), a stacked bar chart stratified by ethnicity and migration generation (B), and the relative abundance of the different clusters stratified by the four enterotypes (C). *P* = *Prevotella*, CMO = *Christensenellaceae/Methanobrevibacter/Oscillibacter* and BBB = *Bacteroides/Blautia/ Bifidobacterium*.

**Additional file 10: Fig. S9.** Heatmaps depicting different clusters / trophic networks present. The colors of the dendrogram include ASVs that were found in the machine learning model distinguishing the 1^st^ from the 2^nd^ migration generation. The color sidebars depict if the median abundance of the ASV is larger in the 1^st^ or 2^nd^ generation. The heatmap itself consist of Spearman ρ correlation coefficients of ASVs, which are hierarchically clustered and visually split up in different clusters of ASVs, of which the reproducibility is summarized in Tables S6, S7 and S8. The first heatmap is a replica of Fig. 5 which now includes ASV names (A). The other ten heatmaps (B-K) include the 1^st^ and the 2^nd^ generation of each of the five ethnicities.

**Additional file 11: Fig. S10.** Overview of α-diversity measures of the 1^st^ and the 2^nd^ generation of each of the different ethnicities. (A) Shannon effective number, (B) richness, and (C) Faith’s phylogenetic diversity (PD). Asterisks denote an FDR-corrected *p*-value based on the Mann-Whitney U test (*pvalue ≤0.05; **pvalue ≤0.01; ***p-value ≤0.001).

**Additional file 12: Fig. S11.** Comparison of the different clusters observed in all ethnicities between males and females. Asterisks denote an FDR-corrected *p*-value based on the Mann-Whitney U test (*pvalue ≤0.05; **pvalue ≤0.01; ***p-value ≤0.001).

**Additional file 13. **Example form of the microbiota data access agreement of the HELIUS board.

**Additional file 14: **Code describing the visualizations with statistical analyses performed in this work.

## Data Availability

The HELIUS data are owned by the Amsterdam University Medical Centers, location AMC in Amsterdam, The Netherlands. To allow sharing of microbiome data collected in HELIUS with (inter)national researchers, 16 s rRNA sequence analysis has been stored at the European genome-phenome archive (EGA; accession code EGAD00001004106). This requires that access needs to be granted, also because the HELIUS data are stored with relevant phenotypical variables. Access is granted to all researchers affiliated with an internationally recognized research institution who request to use the HELIUS data within the EGA context, after having signed the data transfer agreement (Supplementary file 1). Any researcher can request the data by submitting a proposal to the HELIUS Executive Board as outlined at http://www.heliusstudy.nl/en/researchers/collaboration, by email: heliuscoordinator@amsterdamumc.nl. The HELIUS Executive Board will check proposals if they do not conflict with ethical approvals and informed consent forms of the HELIUS study. The reads from the validation cohort are available from NCBI under the NCBI BioProject accession number PRJEB28687. All code used for generating the main figures and performing the statistical analyses are shown in Supplementary file 2. The custom-made Python script including the machine learning analysis is available at GitHub (https://github.com/EvdVossen/HELIUS_Generations).

## Abbreviations

ASVs: Amplicon sequence variants
BMI: Body mass index
B/P ratio: *Bacteroides* To *Prevotella* ratio
BBB: *Bacteroides*/*Blautia*/*Bifidobacterium*
CMO: *Christensenellaceae*/*Methanobrevibacter*/*Oscillibacter*
HELIUS: Healthy Life in an Urban Setting
PCoA: Principal Coordinate analysis
rRNA: Ribosomal RNA
SCFA: Short-chain fatty acids
USI: USA immigration
XGBoost: Extreme gradient boosting
AUC: Area under the curve
