# Supplementary figures and images for: Gut microbiome transitions across generations in different ethnicities in an urban setting—the HELIUS study
Source: Microbiome. 2023 May 8;11:99. doi: 10.1186/s40168-023-01488-z (PMC10165778; doi:10.1186/s40168-023-01488-z)

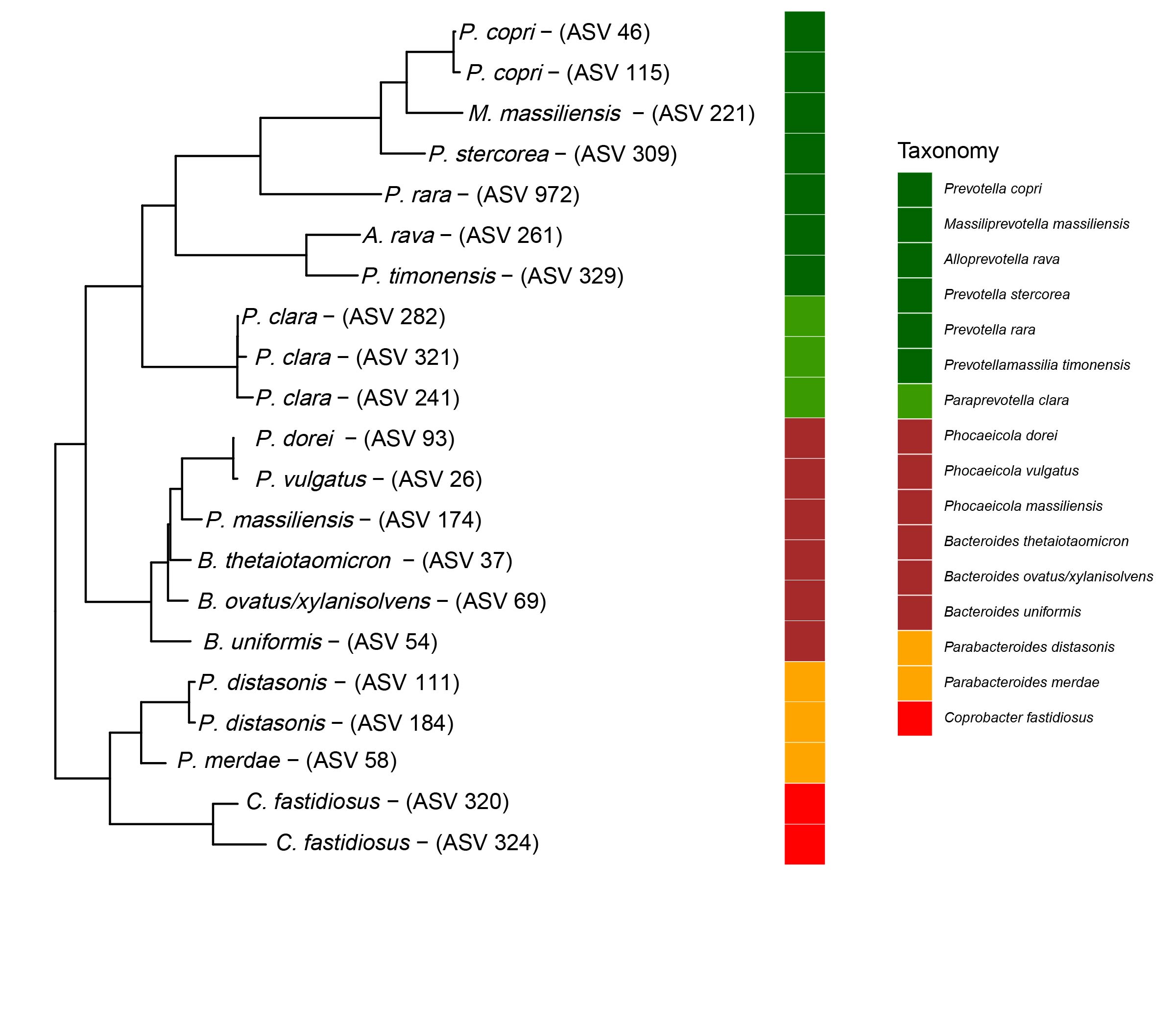

Supplement: Supplementary file 3 — Additional file 2: Fig. S1. Bacteroides, Phocaeicola (formerly also classified as Bacteroides) and Prevotella ASV selection based on phylogeny. [file 40168_2023_1488_MOESM2_ESM.jpg]

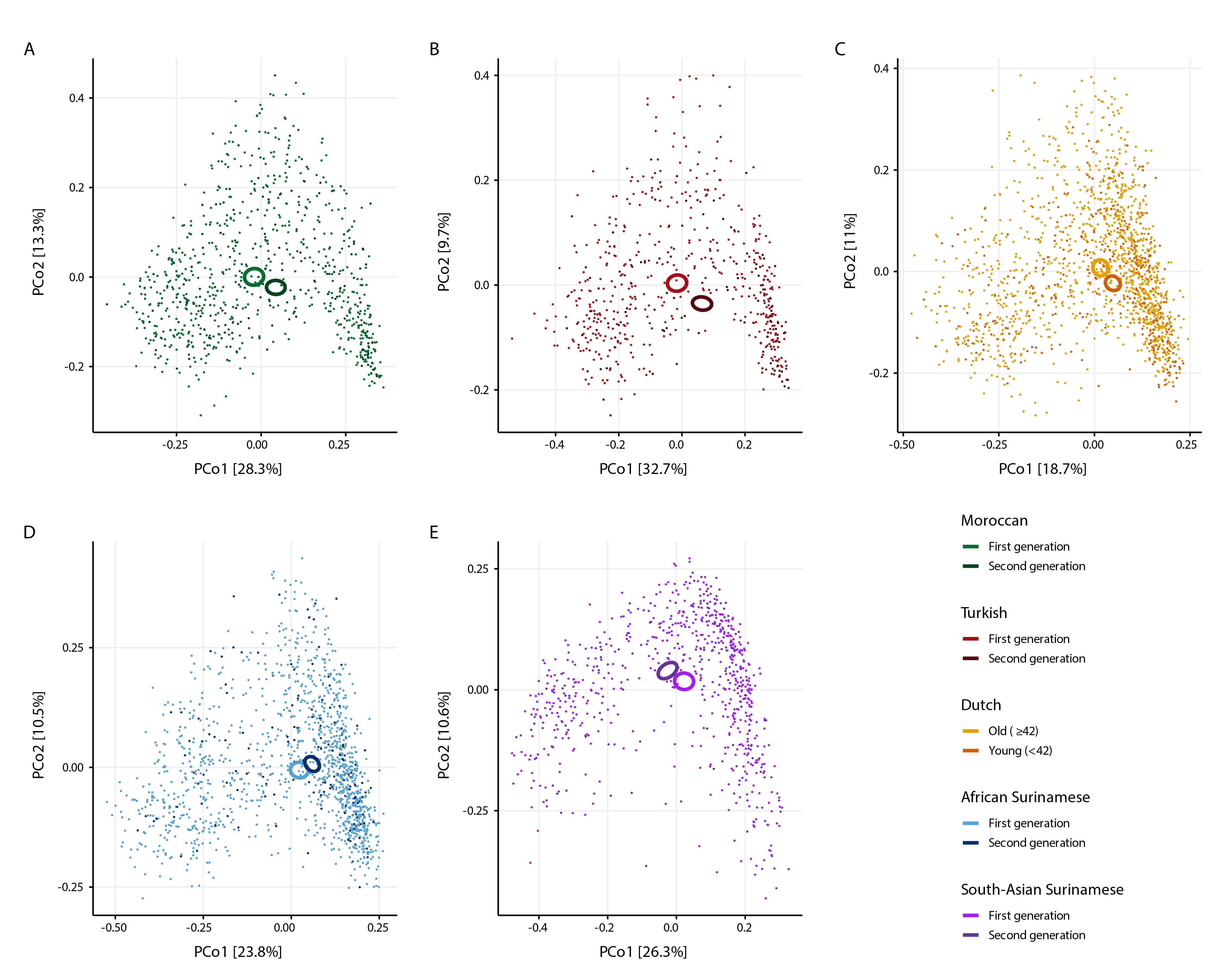

Supplement: Supplementary file 4 — Additional file 3: Fig. S2. PCoA plot representing the interindividual differences in the gut microbiota of the different ethnicities. PERMANOVA is based on the Bray-Curtis distance on each ethnicity. Formula used: Bray-curtis ~ Migration generation + age. The results for (A) Moroccan, migration generation R2 = 0.00561; p≤0.001, age R2 = 0.00332, p=0.007; (B) Turkish, migration generation R2 = 0. 00964; p≤0.001, age R2 = 0.00332, p=0.046; (C) Dutch artificial migration generation R2 = 0. 006; p≤0.001, age R2 = 0.00245, p≤0.001; (D) African Surinamese, migration generation R2 = 0. 00221; p=0.003, age R2 = 0.00269, p≤0.001; (E) South-Asian Surinamese, migration generation R2 = 0.00352; p≤0.010, age R2 = 0.00743, p≤0.001. [file 40168_2023_1488_MOESM3_ESM.jpg]

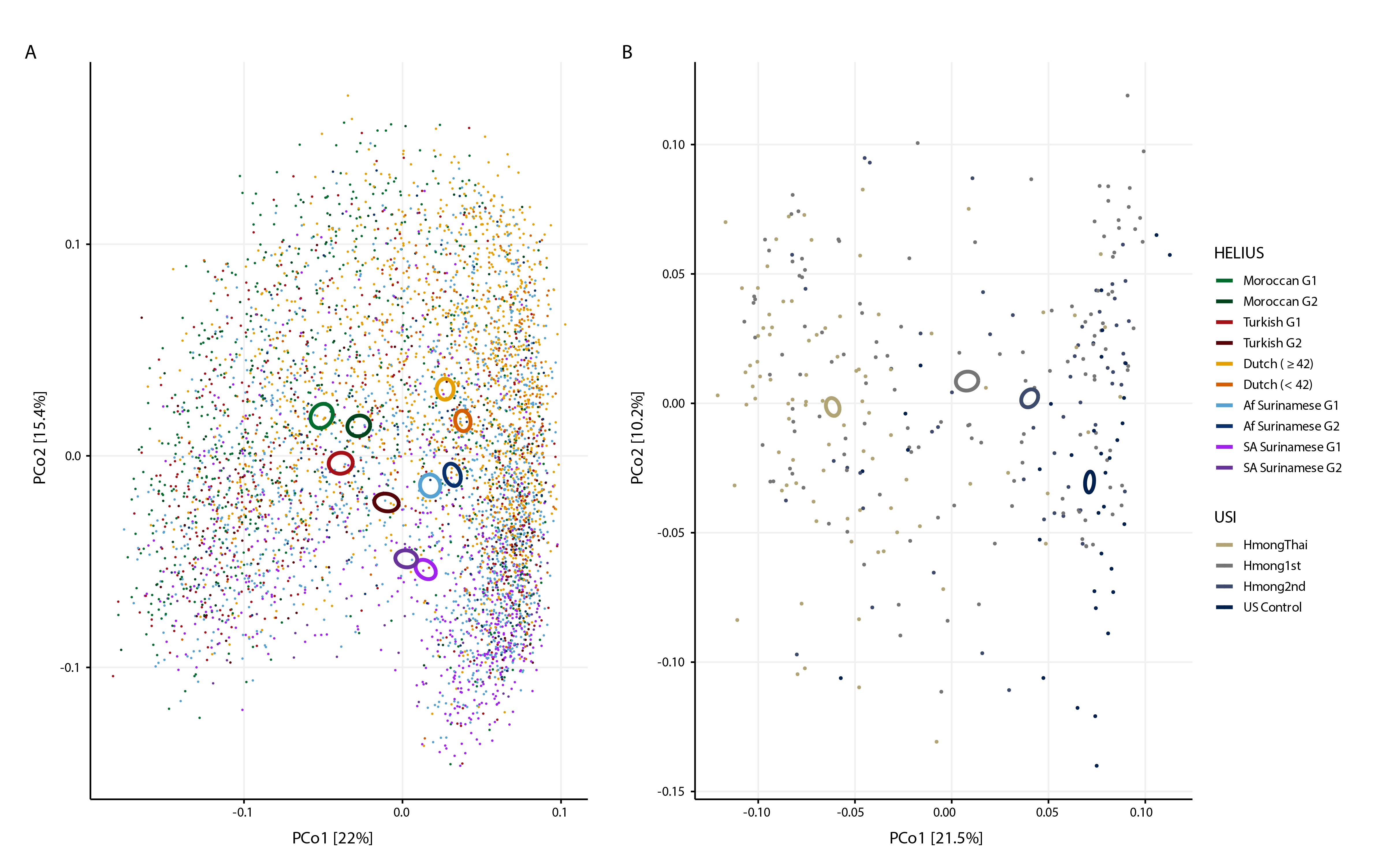

Supplement: Supplementary file 5 — Additional file 4: Fig. S3. PcoA based on the Generalized UniFrac for the HELIUS (A) and USI (B) cohorts. [file 40168_2023_1488_MOESM4_ESM.jpg]

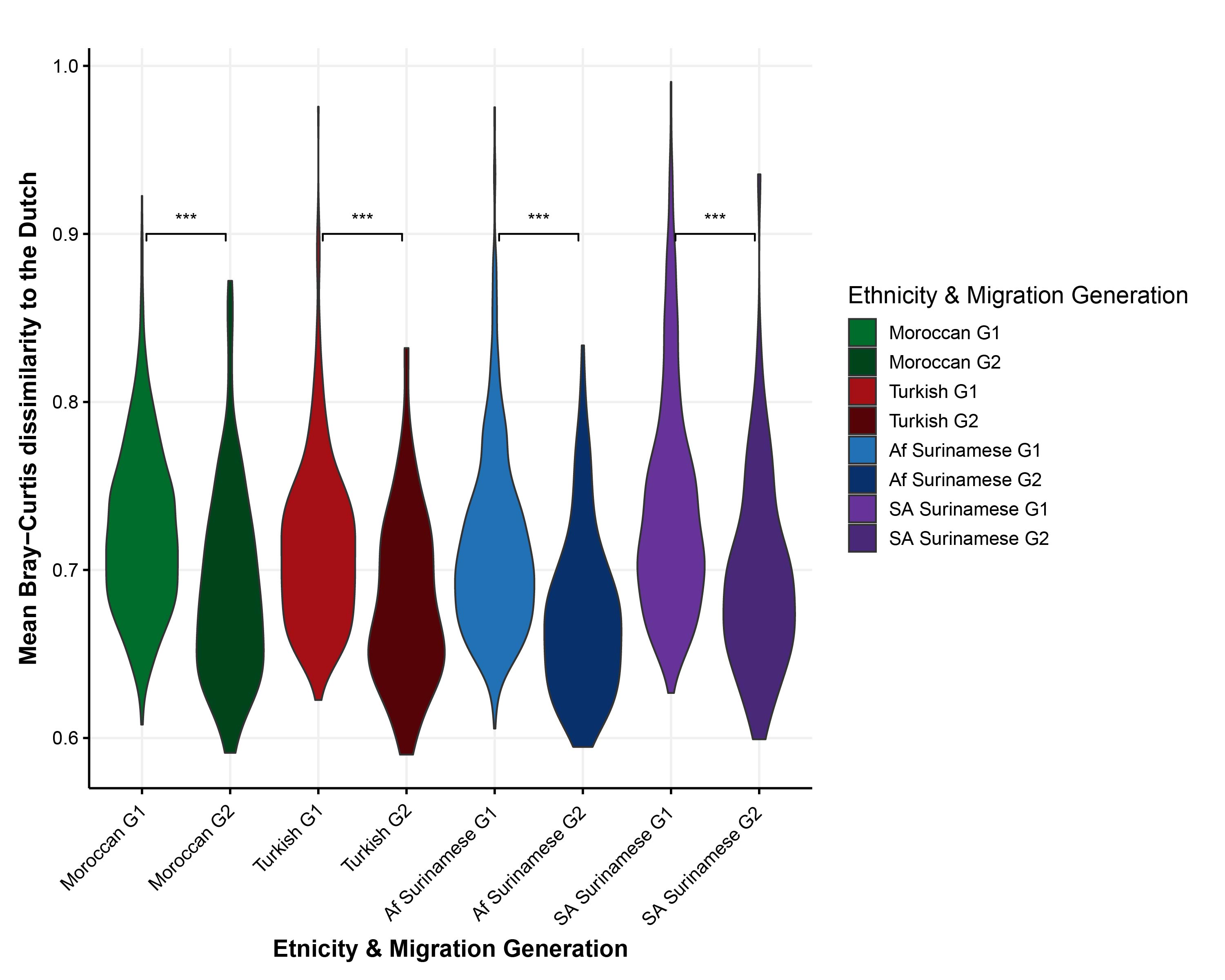

Supplement: Supplementary file 6 — Additional file 5: Fig. S4. Mean Bray-Curtis dissimilarity compared to the Dutch. Here, the 1st generation of each ethnicity was compared to the older aged Dutch (mean age of 57.2) and the 2nd generation to the younger aged Dutch (mean age of 32.4) to correct for age confounding effects. Significance is based on the Mann-Whitney U test (asterisks *p-value ≤0.05; **pvalue ≤0.01; ***p-value ≤0.001). [file 40168_2023_1488_MOESM5_ESM.jpg]

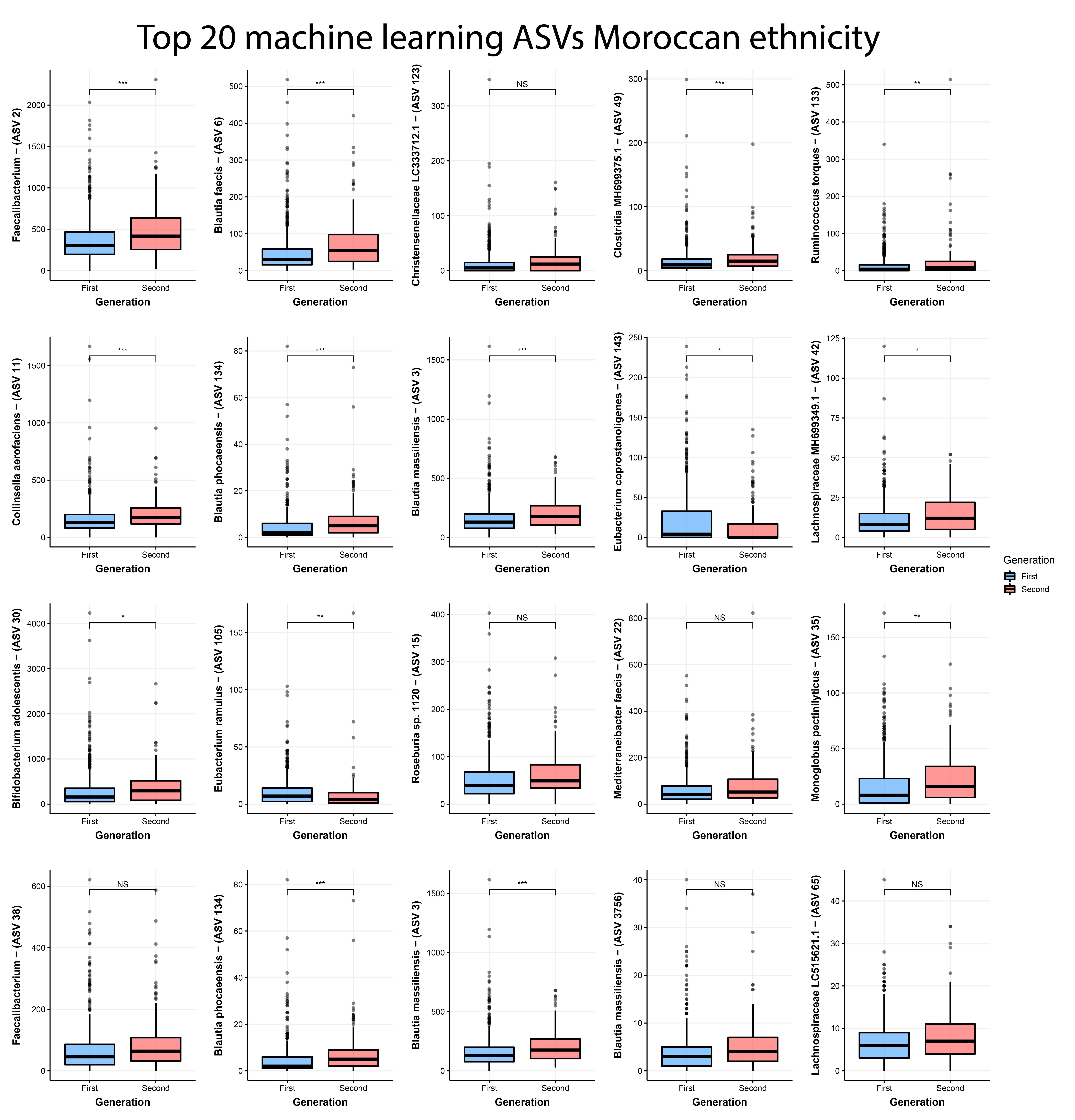

Supplement: Supplementary file 7 — Additional file 6: Fig. S5. Boxplots of the top 20 ASVs found in the different machine learning models of the Moroccan (A), Turkish (B), Dutch (C), African Surinamese (D), South-Asian Surinamese (E), and an age model with all ethnicities (F). Significance is based on the FDR-corrected Mann-Whitney U test (asterisks * p-value ≤0.05; **pvalue ≤0.01; ***p-value ≤0.001). [file 40168_2023_1488_MOESM6_ESM.zip › Figure_S5A.jpg]

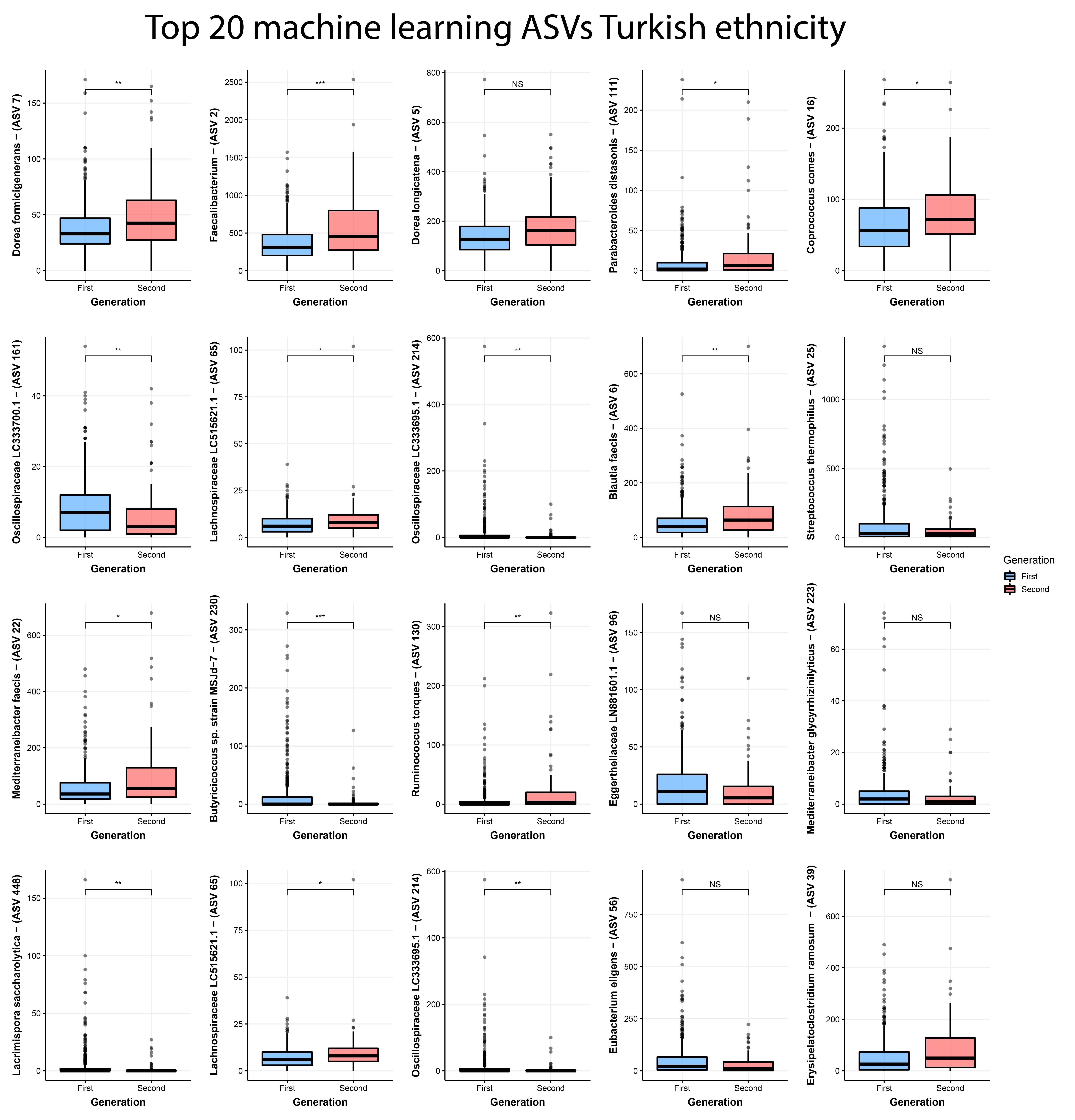

Supplement: Supplementary file 7 — Additional file 6: Fig. S5. Boxplots of the top 20 ASVs found in the different machine learning models of the Moroccan (A), Turkish (B), Dutch (C), African Surinamese (D), South-Asian Surinamese (E), and an age model with all ethnicities (F). Significance is based on the FDR-corrected Mann-Whitney U test (asterisks * p-value ≤0.05; **pvalue ≤0.01; ***p-value ≤0.001). [file 40168_2023_1488_MOESM6_ESM.zip › Figure_S5B.jpg]

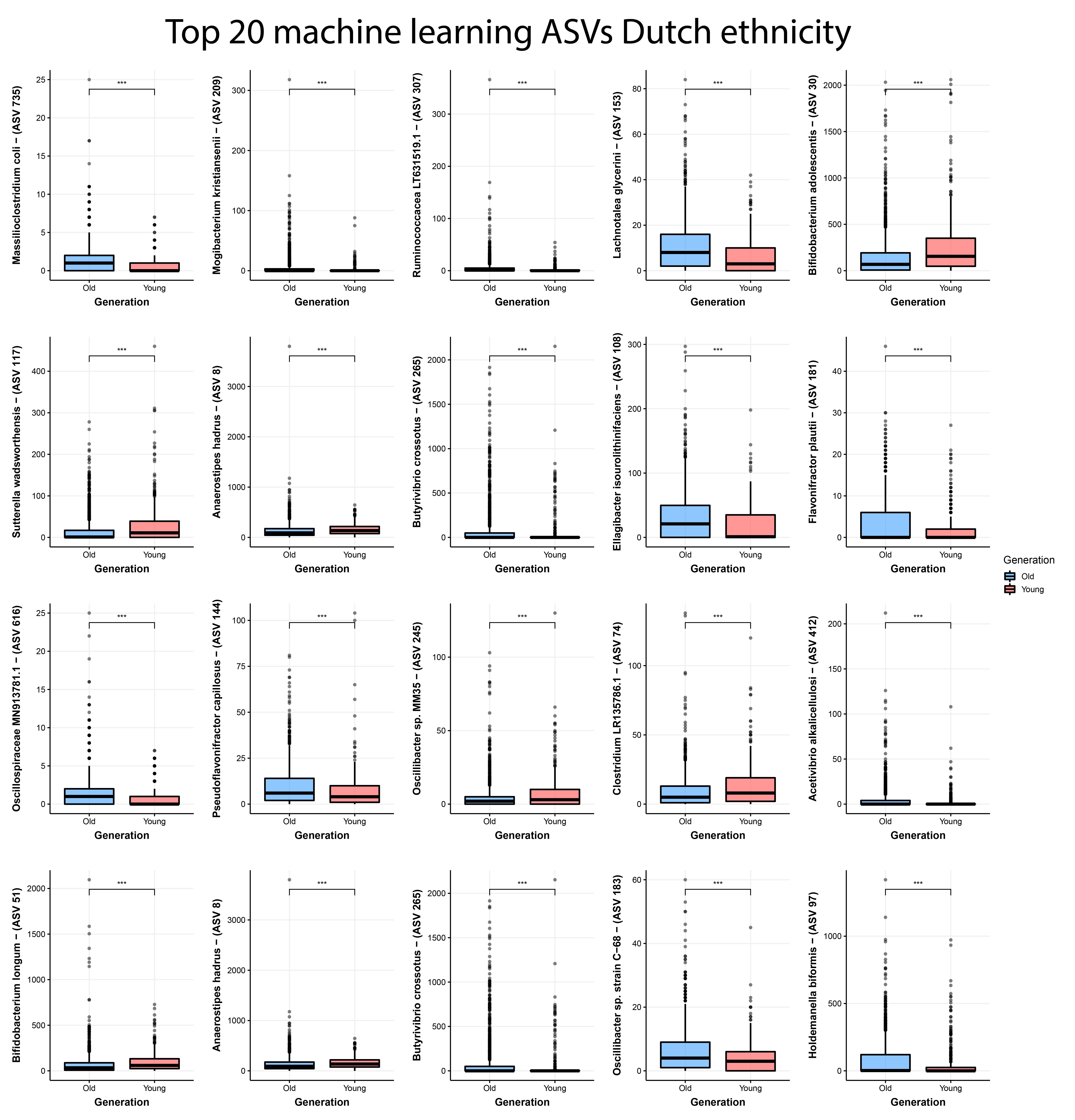

Supplement: Supplementary file 7 — Additional file 6: Fig. S5. Boxplots of the top 20 ASVs found in the different machine learning models of the Moroccan (A), Turkish (B), Dutch (C), African Surinamese (D), South-Asian Surinamese (E), and an age model with all ethnicities (F). Significance is based on the FDR-corrected Mann-Whitney U test (asterisks * p-value ≤0.05; **pvalue ≤0.01; ***p-value ≤0.001). [file 40168_2023_1488_MOESM6_ESM.zip › Figure_S5C.jpg]

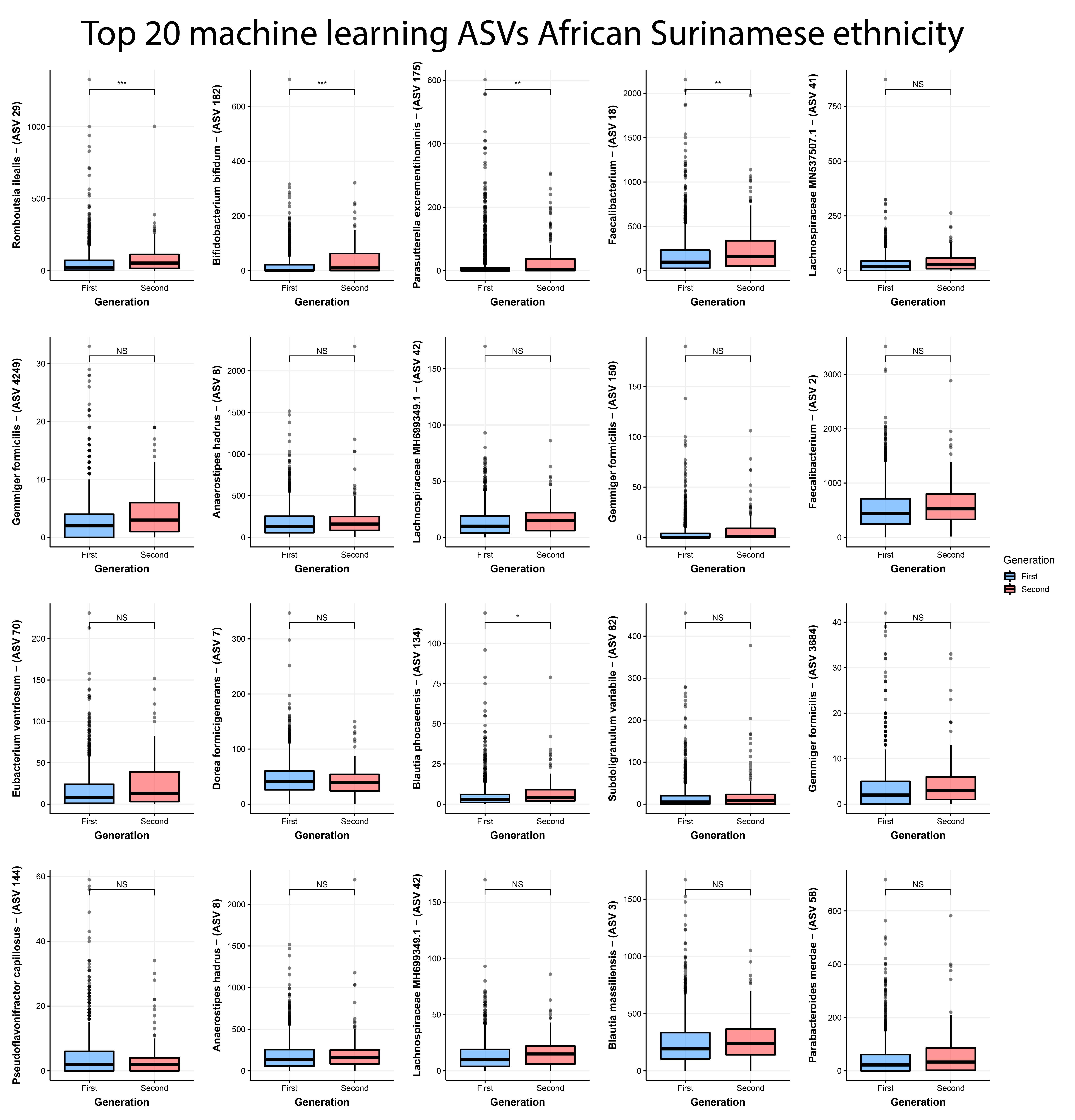

Supplement: Supplementary file 7 — Additional file 6: Fig. S5. Boxplots of the top 20 ASVs found in the different machine learning models of the Moroccan (A), Turkish (B), Dutch (C), African Surinamese (D), South-Asian Surinamese (E), and an age model with all ethnicities (F). Significance is based on the FDR-corrected Mann-Whitney U test (asterisks * p-value ≤0.05; **pvalue ≤0.01; ***p-value ≤0.001). [file 40168_2023_1488_MOESM6_ESM.zip › Figure_S5D.jpg]

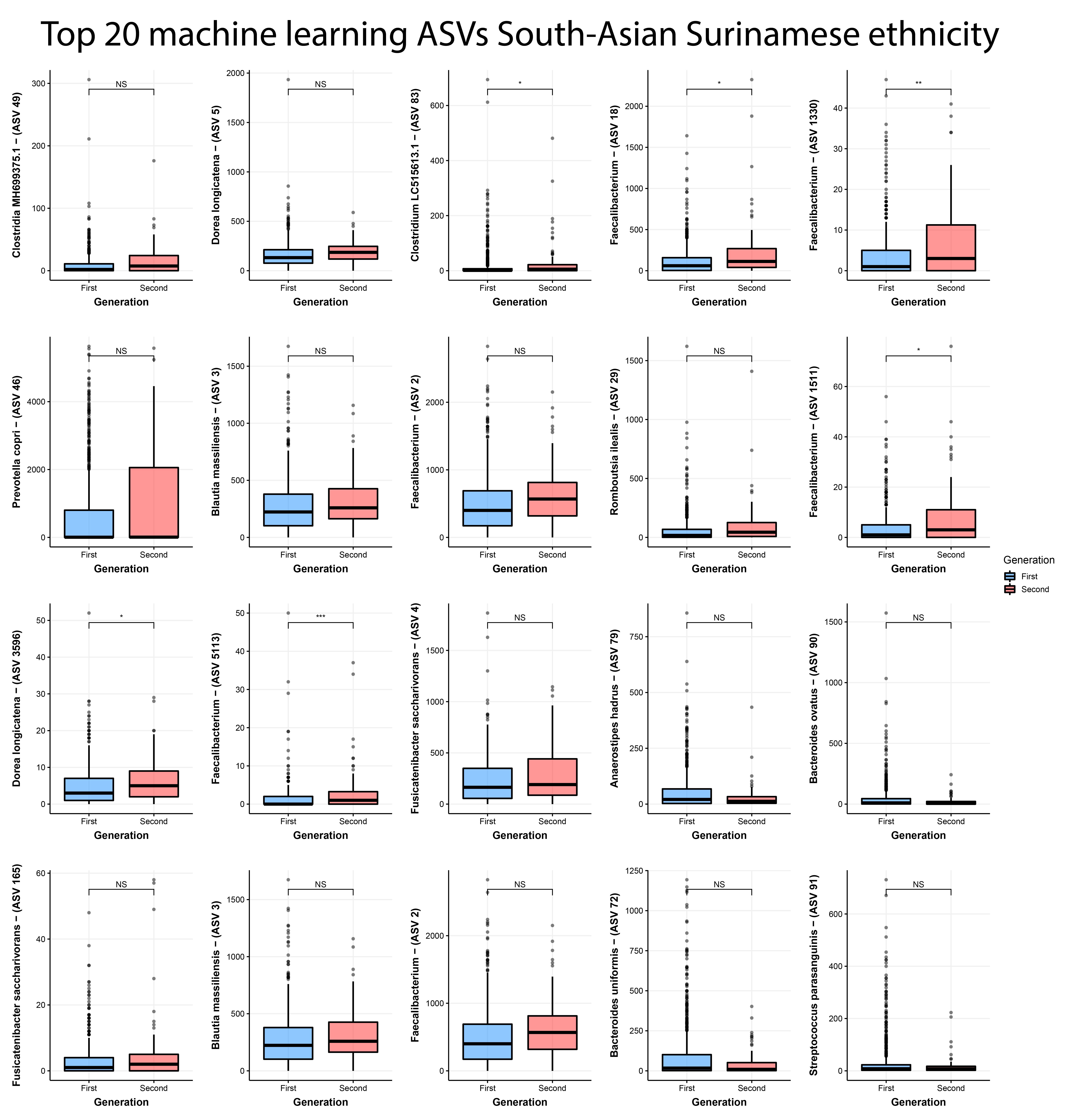

Supplement: Supplementary file 7 — Additional file 6: Fig. S5. Boxplots of the top 20 ASVs found in the different machine learning models of the Moroccan (A), Turkish (B), Dutch (C), African Surinamese (D), South-Asian Surinamese (E), and an age model with all ethnicities (F). Significance is based on the FDR-corrected Mann-Whitney U test (asterisks * p-value ≤0.05; **pvalue ≤0.01; ***p-value ≤0.001). [file 40168_2023_1488_MOESM6_ESM.zip › Figure_S5E.jpg]

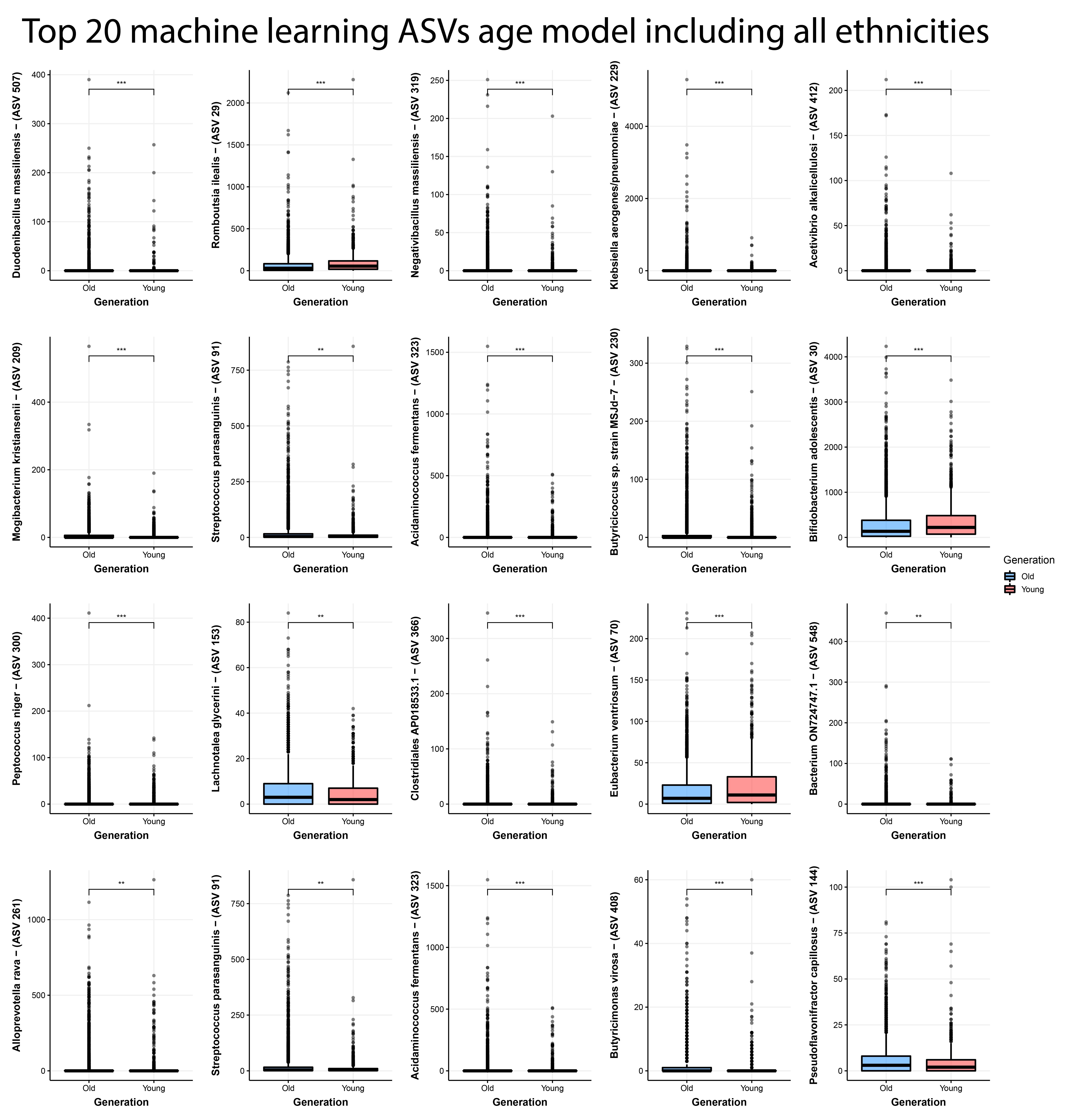

Supplement: Supplementary file 7 — Additional file 6: Fig. S5. Boxplots of the top 20 ASVs found in the different machine learning models of the Moroccan (A), Turkish (B), Dutch (C), African Surinamese (D), South-Asian Surinamese (E), and an age model with all ethnicities (F). Significance is based on the FDR-corrected Mann-Whitney U test (asterisks * p-value ≤0.05; **pvalue ≤0.01; ***p-value ≤0.001). [file 40168_2023_1488_MOESM6_ESM.zip › Figure_S5F.jpg]

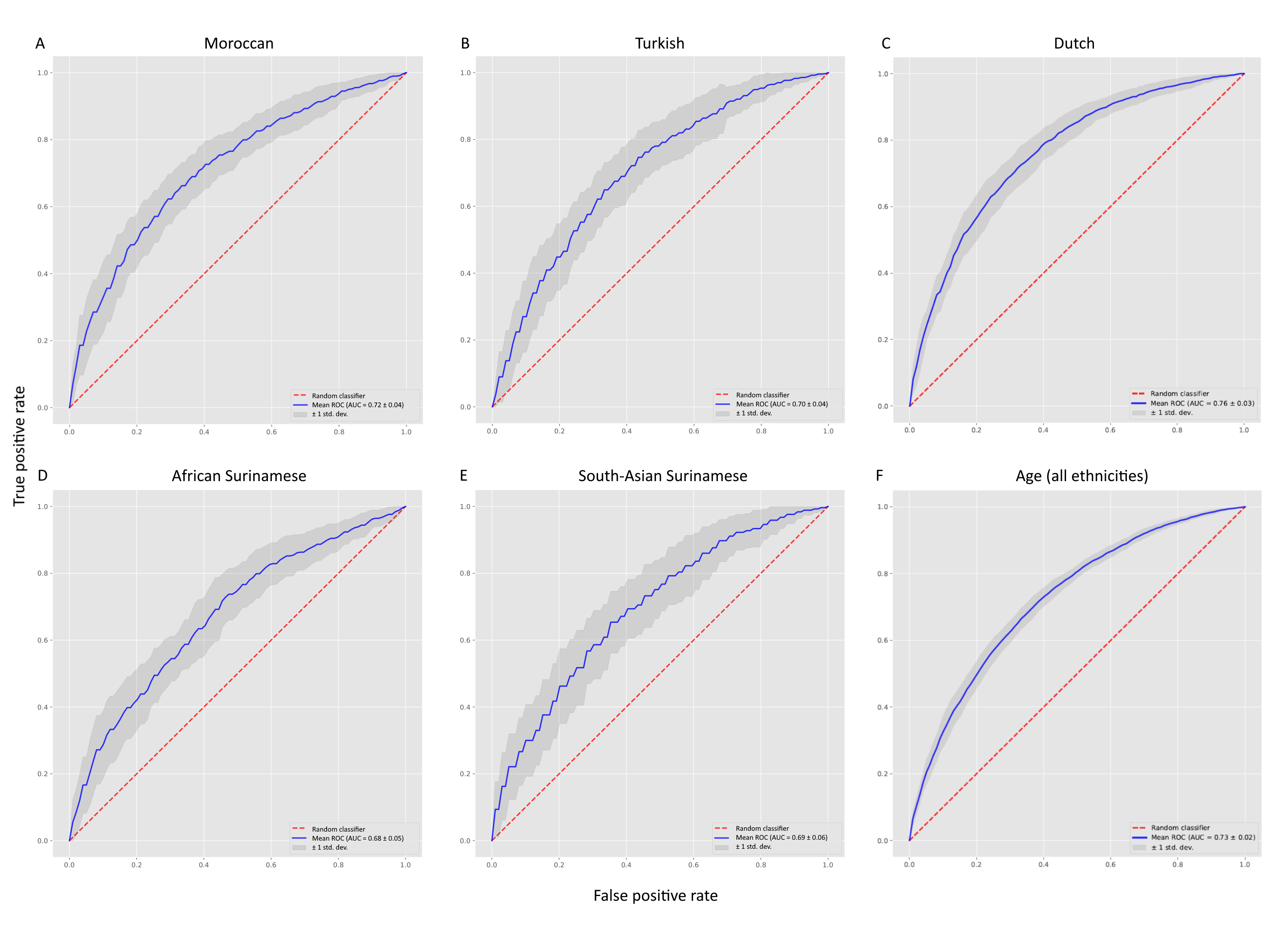

Supplement: Supplementary file 8 — Additional file 7: Fig. S6. ROC-AUC scores of the different machine learning simulations. The different ethnicities are Moroccan (A), Turkish (B), Dutch (C) African Surinamese (D), South-Asian Surinamese (E). Lastly, an age-only model based on all ethnicities at a cut-off of 42 years old was applied (F). [file 40168_2023_1488_MOESM7_ESM.jpg]

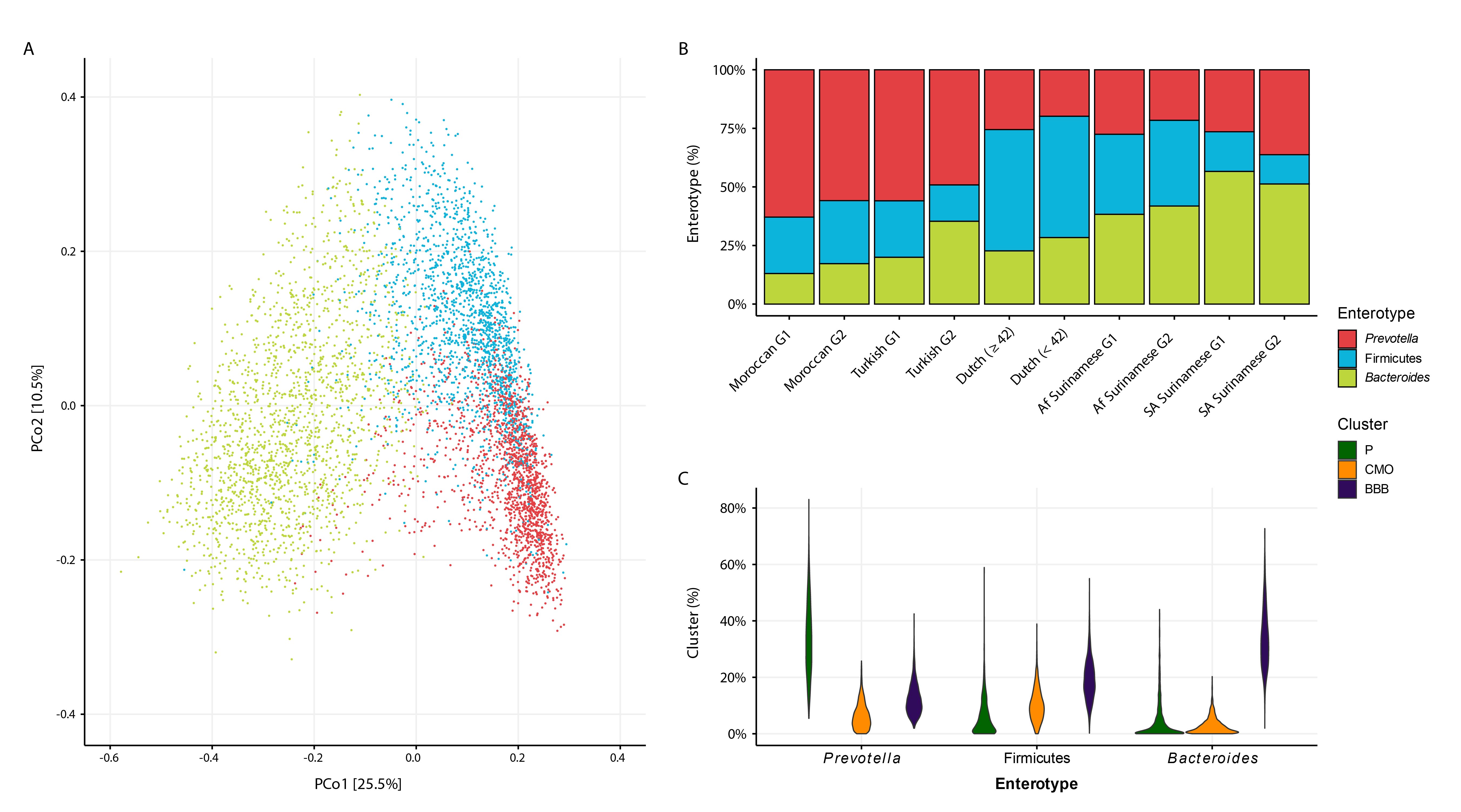

Supplement: Supplementary file 9 — Additional file 8: Fig. S7. Classical three enterotype division described by Arumugam et al. [34] of the HELIUS cohort including a PCoA plot based on the Bray-Curtis dissimilarity (A), a stacked bar chart stratified by ethnicity and migration generation (B), and the relative abundance of the different clusters stratified by the classical three enterotyping (C). P = Prevotella, CMO = Christensenellaceae/Methanobrevibacter/Oscillibacter and BBB = Bacteroides/Blautia/Bifidobacterium. [file 40168_2023_1488_MOESM8_ESM.jpg]

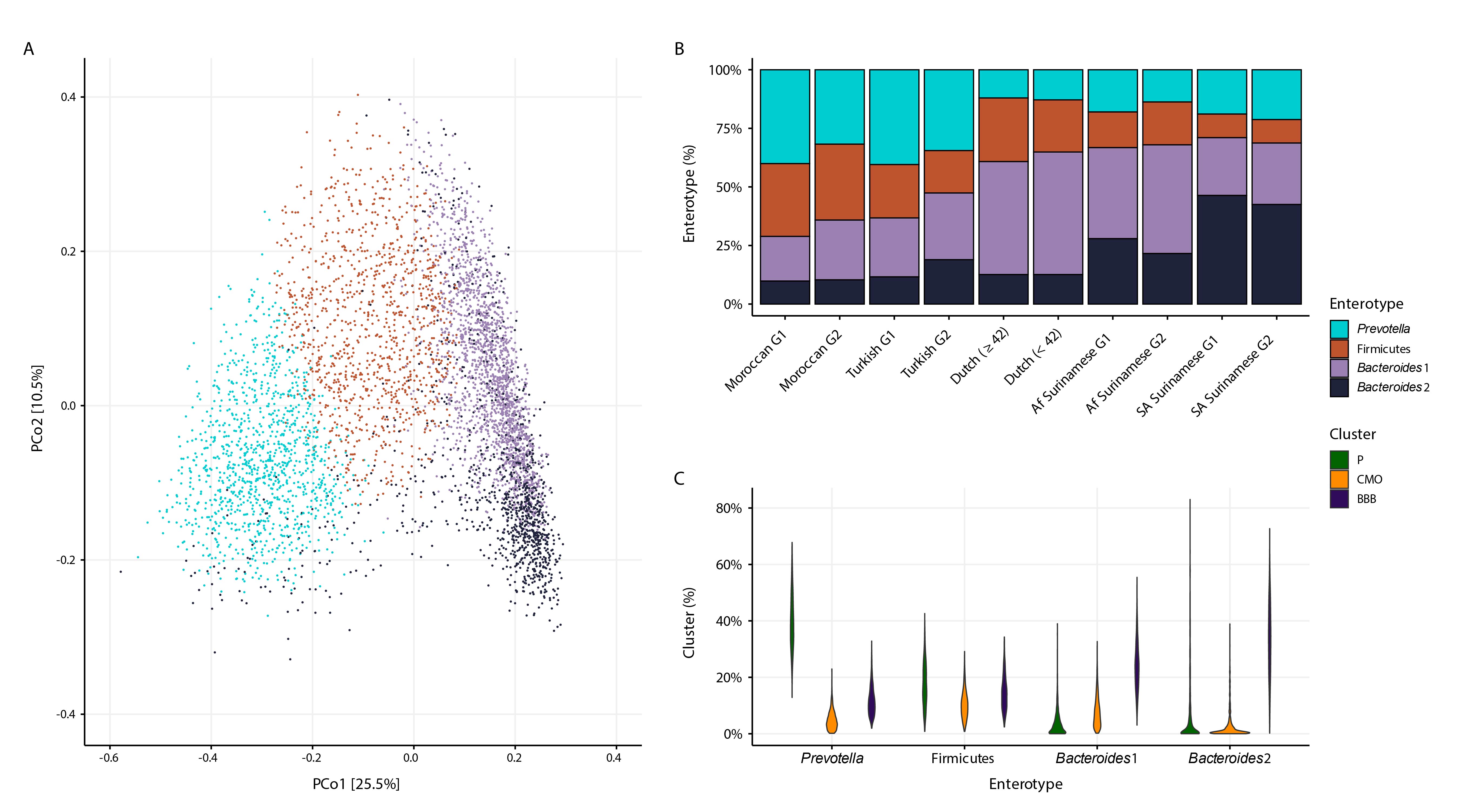

Supplement: Supplementary file 10 — Additional file 9: Fig. S8. Four enterotype division based on the Dirichlet Multinomial Mixture model described by Holmes et al. [35] of the HELIUS cohort including a PCoA plot based on the Bray-Curtis dissimilarity (A), a stacked bar chart stratified by ethnicity and migration generation (B), and the relative abundance of the different clusters stratified by the four enterotypes (C). P = Prevotella, CMO = Christensenellaceae/Methanobrevibacter/Oscillibacter and BBB = Bacteroides/Blautia/ Bifidobacterium. [file 40168_2023_1488_MOESM9_ESM.jpg]

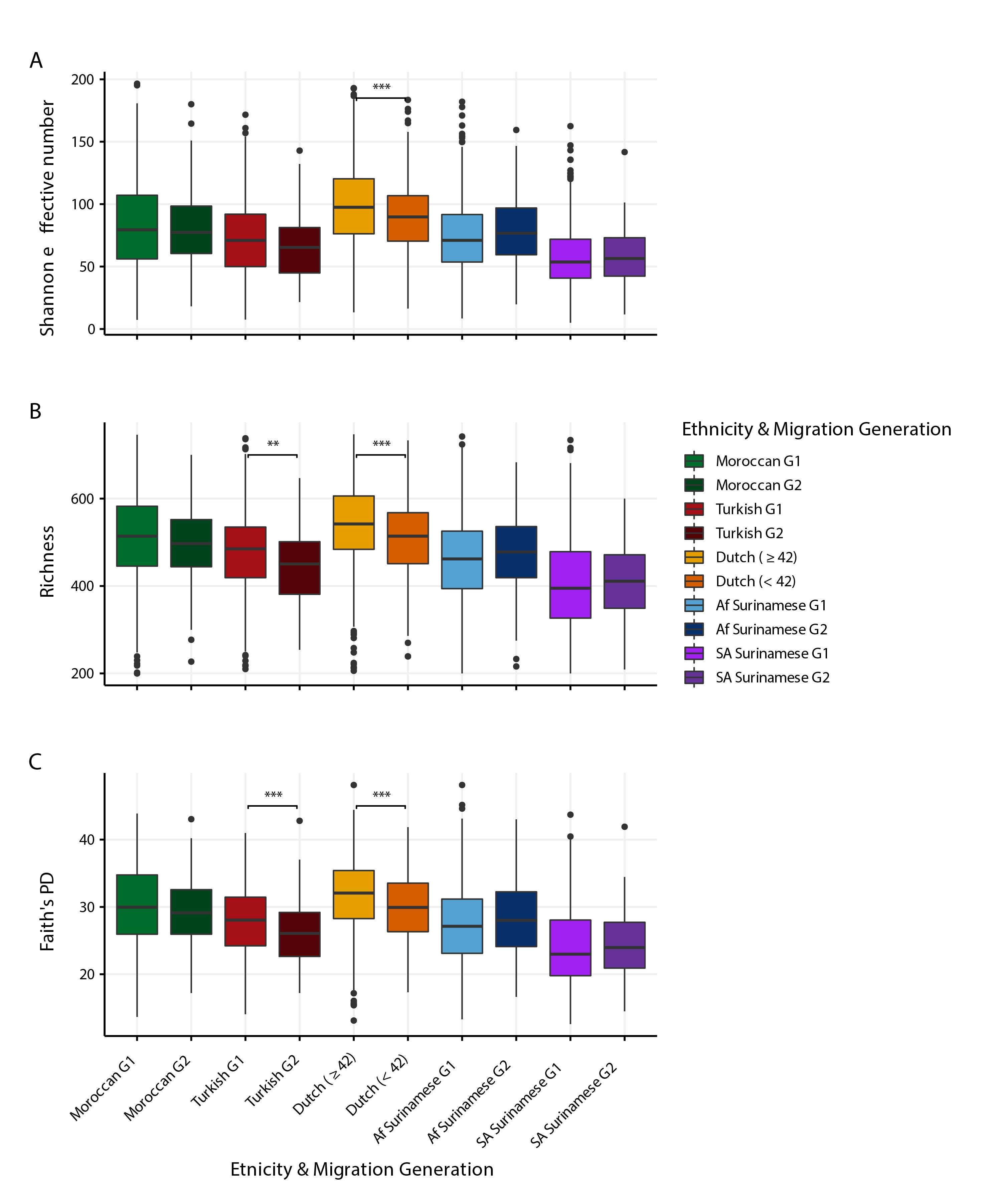

Supplement: Supplementary file 12 — Additional file 11: Fig. S10. Overview of α-diversity measures of the 1st and the 2nd generation of each of the different ethnicities. (A) Shannon effective number, (B) richness, and (C) Faith’s phylogenetic diversity (PD). Asterisks denote an FDR-corrected p-value based on the Mann-Whitney U test (*pvalue ≤0.05; **pvalue ≤0.01; ***p-value ≤0.001). [file 40168_2023_1488_MOESM11_ESM.jpg]

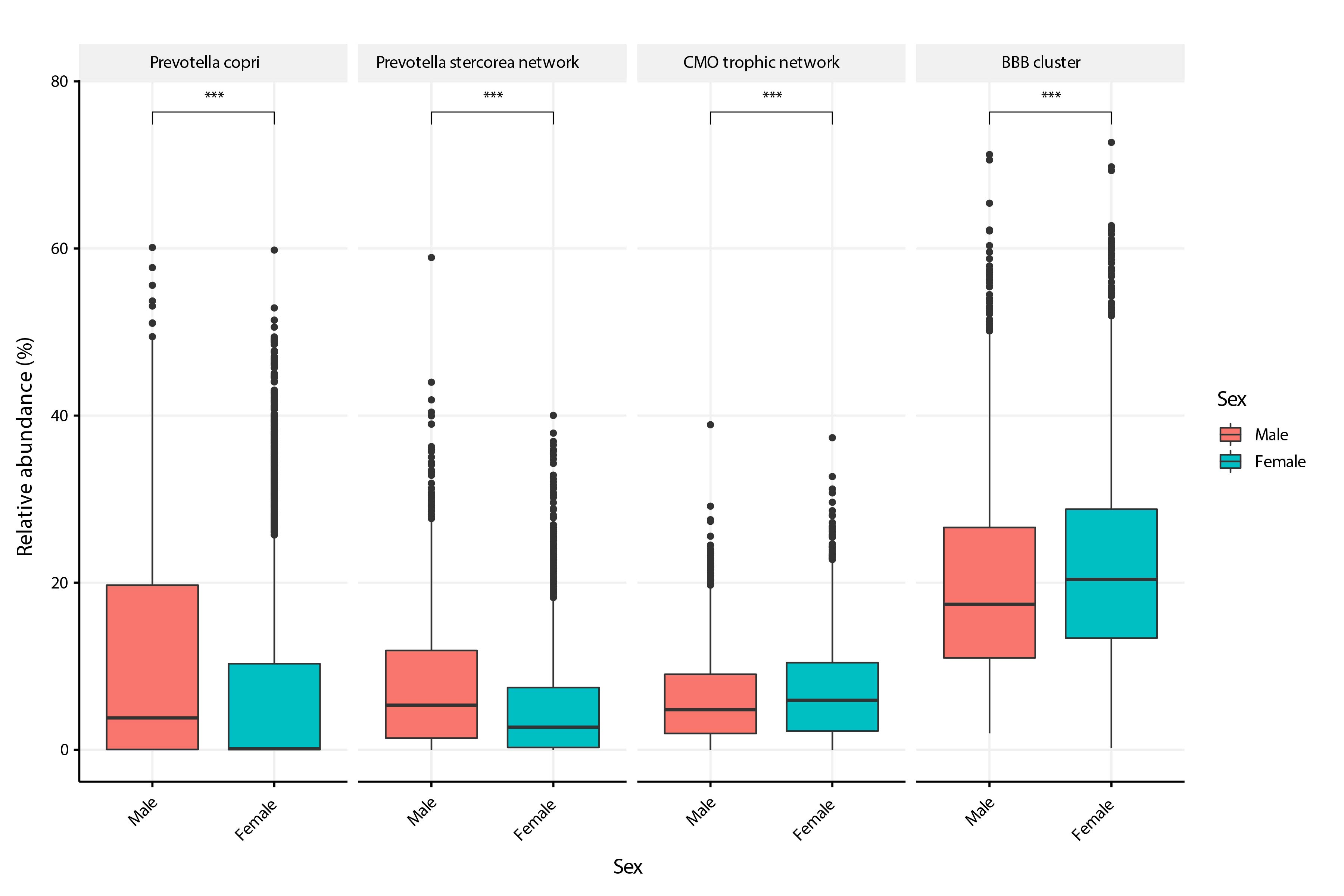

Supplement: Supplementary file 13 — Additional file 12: Fig. S11. Comparison of the different clusters observed in all ethnicities between males and females. Asterisks denote an FDR-corrected p-value based on the Mann-Whitney U test (*pvalue ≤0.05; **pvalue ≤0.01; ***p-value ≤0.001). [file 40168_2023_1488_MOESM12_ESM.jpg]
