# Supplementary material for: Gut microbiome transitions across generations in different ethnicities in an urban setting—the HELIUS study
Source: Microbiome. 2023 May 8;11:99. doi: 10.1186/s40168-023-01488-z (PMC10165778; doi:10.1186/s40168-023-01488-z)
Supplement: Supplementary file 14 — Additional file 13. Example form of the microbiota data access agreement of the HELIUS board. [file 40168_2023_1488_MOESM13_ESM.pdf]

Microbiota Data Access Agreement #……

This agreement is made **……..(day) - ……..(month) - ……..(year)** between:

The Academic Medical Center (“**AMC**”) acting on behalf of the HELIUS Study ("HELIUS"), Meibergdreef 9, 1105 AZ, Amsterdam, The Netherlands, legally represented by the Chair of the Executive Board of HELIUS, **Prof. K Stronks**,

And

…………………………………(institute) (hereafter referred to as “the Recipient”)

Hereafter referred to collectively as “Parties” and individually as “Party”.

BACKGROUND

The AMC, through HELIUS, has collected and owns certain data sets derived from information provided by participants in the HELIUS Study (“the Study Participants”). An employee of the Recipient (“Investigator”), wishes to use the Pseudonymised Microbiota Data held by HELIUS (hereafter named “Research Data”) as set out in the Data Access Application set out in Appendix 1 (“the Research”) as approved by the HELIUS Executive Board. HELIUS is willing to supply the Recipient with a copy of the requested Data for a period of **12 months** (1 year) to conduct the Research under the terms and conditions of this Agreement.

DEFINITIONS

a) “Controller”, “Data subject”, “Personal data”, “Processing” and “Processor” shall have the meaning as in the General Data Protection Regulation (EU) 2016/679 (hereinafter: “GDPR”) where these terms appear in non-capitalized words.

b) “Pseudonymised data” means Personal data which can no longer be attributed to a specific Data subject without the use of additional information, provided that such additional information is kept separately and is subject to technical and organisational measures to ensure that the Personal Data are not attributed to an identified or identifiable natural person. This additional information will be stored by HELIUS, to which the Recipient will not have access.

NOW IT IS AGREED by Parties as follows:

1. This agreement does not affect the ownership of any Material and/or Research Data. No license to use any intellectual property is granted or implied by this Agreement except the rights expressly granted in this Agreement.
2. AMC will provide the Recipient with a copy of the Research Data in the form of Pseudonymised data that the Recipient needs for the Research. Research Data shall be made available of the type and in the quantities as outlined in the Appendix 2 (“Research Data”). Parties acknowledge that the Research Data are Personal data. The Research Data shall be provided for the sole purpose of its use in the Research and in accordance with the terms of this Agreement.
3. AMC and the Recipient are considered joint Controller with regard to the Research Data to the extent processed by virtue of the present Agreement and for the period the Research Data are under the control of the Recipient. In addition, each Party may be considered an independent Controller in relation to its own processing of the Research Data. Therefore, in Processing the Research Data each Party will be bound to the provisions in the GDPR related to the Controller and abide to the additional data protection laws of the Netherlands. In addition, the Recipient warrants to Process the Research Data in accordance with its applicable national law, provided that in case of inconsistencies between the GDPR and national law, the provisions of the GDPR shall prevail.
4. AMC warrants and undertakes:

   a. the Research Data have been collected, processed and transferred in accordance with the GDPR and additional data protection laws in the Netherlands.

   b. it has obtained any regulatory or ethics approvals necessary to collect the Data (including the Research Data) and to Process them for the purpose of HELIUS;

   c. it has full authority to transfer the Research Data to the Recipient; and

   d. informed consent of the Data subject has been obtained in accordance with applicable law.
5. All future correspondence pertaining to the Research Data and the Research should be addressed to HELIUS (HELIUS coordinator, HELIUScoordinator@amsterdamumc.nl).
6. The Recipient will use the Research Data only to carry out the Research described in the Appendix 1 to this Agreement. The Recipient will not use the Research Data or any parts thereof for any commercial purpose or any purpose that is subject to consulting or licensing obligations to third parties. Recipient shall ensure that only those employees of Recipient shall have access to the Research Data, who are involved in the Research under direct supervision of the Investigator.
7. The Recipient will not try identify any Data subject nor link the Research Data to other HELIUS Data held by different recipients or by the same Recipient for different projects.

8. The Recipient will treat all Research Data strictly confidential and not transfer the Research Data in whole or in part to third parties. Consequently, Recipient shall not without first obtaining the consent of Provider:

   a. engage a (sub)Processor to process Research Data on behalf of the Recipient.

   b. transfer any (portion of) the Research Data to any affiliate of Recipient or third-party located outside the European Economic Area.

   In case the Provider approves to any of the above, Recipient shall have in place procedures ensuring that any such (sub)Processors, affiliates and/or third-parties will respect and maintain the confidentiality and security of the Research Data and shall Process the Research Data in accordance with the requirements of the GDPR and be bound to provisions similar to those of this Agreement. Recipient shall be responsible to Provider for any acts of the such (sub)Processors, affiliates and/or third-parties as if Recipient had performed the Processing itself.

9. Subject to Section 8, any person or organisation acting under the authority of the Recipient shall be obligated to Process the Research Data only on instructions from the Recipient and in accordance with the permitted use under this Agreement.
10. Recipient shall not be prevented to provide access to the Research Data to persons authorised or required by law or regulation to have access to the Research Data, provided it will without undue delay inform HELIUS of any projected or actual inspection.
11. Recipient shall be responsible to have an agreement in place with each of its employees who have access to the Research Data, which agreement binds them to provisions of confidentiality and to process any Research Data in accordance with the GDPR. The Investigator and other relevant employees of the Recipient involved in the Research have read and will abide by the "HELIUS Collaboration Policy".
12. The Investigator will retain the Research Data in a secure location on its premises and warrants that it will have for the full duration the Research Data are in its custody, appropriate technical and organisational measures in place to protect the Research Data against accidental or unlawful destruction or accidental loss, alteration, unauthorised disclosure or access, and which provide a level of security appropriate to the risk represented by the processing and the nature of the Research Data to be protected.
13. The Recipient will use all reasonable endeavors to ensure that the Research Data in its possession, or under the control of the Recipient shall as soon as possible be returned or destroyed upon (i) the reasonable request of HELIUS; or (ii) on termination of this Agreement; or (iii) in the event that the Recipient is in breach of any of the conditions of this Agreement. If the Recipient is required to destroy

the Research Data then it will ensure that this is done in compliance with all applicable laws and regulations and confirm in writing to HELIUS that the Research Data has been destroyed.

14. If the Recipient becomes aware of a (potential) personal data breach affecting the Data, or receives a request of a Data subject to exercise its rights under the GDPR, the Recipient shall promptly notify the AMC. In case of a personal data breach, Parties will fully cooperate with each other to remedy the personal data breach, fulfil their respective (statutory) notification obligations timely and cure the damages. A personal data breach shall have the meaning under articles 33 and 34 of the GDPR. In case of a request of a Data subject to exercise its rights under the GDPR, the Parties agree that HELIUS is most suited to handle such requests. However, the Recipient shall fully cooperate with HELIUS in handling such requests, if so requested by HELIUS.
15. The Recipient will keep HELIUS informed of the results of the Research ("Results"). All information (including the results of cleaned or derived variables) relating directly to Study Participants will be made available to AMC for the purposes of incorporation into HELIUS. All other results generated by the Research shall be the property of the Recipient save that the Recipient grants to the AMC royalty-free, irrevocable, perpetual non-exclusive right to use such Results for internal non-commercial research and teaching. The Recipient will provide HELIUS with a fully documented electronic copy of the full Results before publication in any form or within 6 months of the completion of the Research whichever is the sooner. In addition, for archival purposes, the Recipient will provide HELIUS with the complete dataset (including derived variables) and syntaxes which document how the Results of the publication were obtained.
16. This Agreement does not affect the ownership of the Research Data. No license to use any intellectual property is granted or implied by this Agreement except the rights expressly granted in this Agreement.
17. HELIUS accepts no liability in connection with the Recipients use of the Research Data. HELIUS does not represent that (i) the Research Data is of satisfactory quality or fit for any particular purpose; or (ii) use of the Research Data is free from infringement of third party rights, including intellectual property rights. To the extent permissible by law the Recipient will indemnify and hold HELIUS harmless for any damages howsoever arising from Recipient's use of the Research Data.
18. The rights and obligations as determined in the Agreement may not be assigned by a Party without the prior written consent of the other Party.
19. This Agreement shall be interpreted and governed by the laws of The Netherlands in any action. Any dispute relating to the interpretation or implementation of this Agreement which the Parties hereto have failed to settle amicably shall be exclusively referred to the competent courts of The Netherlands for settlement.
20. Modifications and changes to this Agreement are only binding after these have been agreed upon in writing between the Parties.

Each person signing this Agreement represents and warrants that he or she is duly authorized and has legal capacity to execute and deliver this Agreement.

AGREED BY THE PARTIES through their authorized signatories

SIGNED for and on behalf of AMC:

Signature: ……………………………………………….

Print Name: **K. Stronks** Date: ……………………………..

SIGNED for and on behalf of RECIPIENT:

Signatory of Recipient …………………………. …

Printed Name: ……………………………… Date:……………………………

(1) Signature of Investigator ……………………………

Printed Name: ………………………………. Date:……………………………

(2) Signature of Investigator ……………………………

Printed Name: ………………………………. Date:……………………………

APPENDIX 1: **Data access application**

Please note: A project specific appendix to be added here which must be approved by HELIUS.

APPENDIX 2: **Categories of Data subjects and categories of Research Data**

Supplier shall make certain Research Data available to Recipient, including data that relates to human tissue samples.

I. The Research Data includes the following categories of Data subjects:

Participants of the HELIUS study

In the amount of [number] Data subjects

II. The Research Data includes the following Personal Data:
Microbiota data

III. Research Data is Pseudonymised data, to ensure data minimalisation under the GDPR.
