# Supplementary material for: Gut microbiome transitions across generations in different ethnicities in an urban setting—the HELIUS study
Source: Microbiome. 2023 May 8;11:99. doi: 10.1186/s40168-023-01488-z (PMC10165778; doi:10.1186/s40168-023-01488-z)
Supplement: Supplementary file 15 — Additional file 14: Code describing the visualizations with statistical analyses performed in this work. [file 40168_2023_1488_MOESM14_ESM.pdf]

Gut microbiome transitions across generations in different ethnicities in an urban setting – The HELIUS study

Eduard W. J. van der Vossen, Mark Davids, Lucas R.F. Bresser, Henrike Galenkamp, Bert Jan van den Born, Koos Zwinderman, Evgeni Levin, Max Nieuwdorp, Marcus C. de Goffau

Last compiled on: 9/12/2022

Preparatory work:

Loading necessary packages

```
source("scripts/R_startup.R")
```

```
#PS HELIUS (cluster level and ASV-level)
ps_h_c <- readRDS("ps_clusters_HELIUS_rarefied15221.RDS")
ps_h_c
```

```
## phyloseq-class experiment-level object
## otu_table()   OTU Table:         [ 561 taxa and 5193 samples ]
## sample_data() Sample Data:       [ 5193 samples by 346 sample variables ]
## tax_table()   Taxonomy Table:    [ 561 taxa by 11 taxonomic ranks ]
## phy_tree()    Phylogenetic Tree: [ 561 tips and 560 internal nodes ]
```

```
ps_h <- readRDS("ps_HELIUS_rarefied15221.RDS")
ps_h
```

```
## phyloseq-class experiment-level object
## otu_table()   OTU Table:         [ 3930 taxa and 5193 samples ]
## sample_data() Sample Data:       [ 5193 samples by 342 sample variables ]
## tax_table()   Taxonomy Table:    [ 3930 taxa by 6 taxonomic ranks ]
## phy_tree()    Phylogenetic Tree: [ 3930 tips and 3929 internal nodes ]
## refseq()      DNAStringSet:      [ 3930 reference sequences ]
```

```
#PS USI
ps_usi <- readRDS("ps_USI_rarefied1163.RDS")
ps_usi
```

```
## phyloseq-class experiment-level object
## otu_table()   OTU Table:         [ 3933 taxa and 635 samples ]
## sample_data() Sample Data:       [ 635 samples by 342 sample variables ]
## tax_table()   Taxonomy Table:    [ 3933 taxa by 6 taxonomic ranks ]
## refseq()      DNAStringSet:      [ 3933 reference sequences ]
```

```
#All Blasted ASVs
stables <- rio::import("Intermediate_files/All_Blasted_ASVs.xlsx")
```

```
dat_h <- data.frame(sample_data(ps_h_c))
clust_h <- as.data.frame(t(otu_table(ps_h_c)))
```

```
colors_h <- c("#006D2C","#00441B","#A50F15","#550307","#E69F00", "#D55E00",
                              "#54a2d2","#08306B", "purple", "#663399")
names_h <- c("Moroccan G1", "Moroccan G2", "Turkish G1", "Turkish G2",
                              "Dutch (Mean age = 57.2)", "Dutch (Mean age = 32.4)",
                              "Af Surinamese G1", "Af Surinamese G2",
                              "SA Surinamese G1", "SA Surinamese G2")
plotnames_h <- c("Moroccan G1", "Moroccan G2", "Turkish G1","Turkish G2",
                                "Dutch (\u2265 42)", "Dutch (\u003c 42)",
                                "Af Surinamese G1","Af Surinamese G2",
                                "SA Surinamese G1", "SA Surinamese G2")
```

Bray-curtis and multidimensional scaling

Large files including Multidimensional scaling data of the Bray-Curtis dissimilarity matrix were saved to avoid additional computation time creating the pdf.

```
#Bray-curtis dissimilarity
#bray <- vegan::vegdist(clust_h, method = 'bray')
# mds <- cmdscale(bray, eig = TRUE, x.ret = TRUE)
mds <- readRDS("Intermediate_files/mds_helius.RDS")
mds.var.per <- round(mds$eig/sum(mds$eig)*100,1)
mds.values <- mds$points
mds.data <- data.frame(Sample=rownames(mds.values),
                       X=mds.values[,1],
                       Y=mds.values[,2])

mds.data$Ethnicity <- dat_h$etn_migrgen[match(mds.data$Sample, dat_h$Sample_ID)]
```

Adding ridegline density plots to the main PCoA

```
order_groups_rdp <- c("SA Surinamese G2", "SA Surinamese G1", "Af Surinamese G2",
                      "Af Surinamese G1", "Dutch (Mean age = 32.4)",
                      "Dutch (Mean age = 57.2)","Turkish G2", "Turkish G1",
                      "Moroccan G2", "Moroccan G1")
rdp <- mds.data
rdp$Ethnicity <- as.factor(rdp$Ethnicity)
rdp$Ethnicity <- factor(rdp$Ethnicity,levels= c(order_groups_rdp))
library(ggridges)
p1 <-  ggplot(rdp, aes(x = X, y = Ethnicity)) +
  geom_density_ridges_gradient(scale = 3, rel_min_height = 0,
                               aes(fill=Ethnicity)) +
  scale_fill_manual(values = rev(colors_h)) +
  xlim(-0.6, 0.4) +
  theme_Publication() +
  theme_void() +
  theme(legend.position = "none")

p2 <- ggplot(rdp, aes(x = Y, y = Ethnicity)) +
  geom_density_ridges_gradient(scale = 3, rel_min_height = 0,
                               aes(fill=Ethnicity)) +
  scale_fill_manual(values = rev(colors_h)) +
  xlim(-0.4, 0.41) +
  coord_flip() +
```

```
  theme_void() +
  theme(legend.position = "none")
```

Adding specific loadings to the PCoA

```
envdat <-
  clust_h[,names(clust_h)=="Cluster_477"|names(clust_h)=="Cluster_486"|
            names(clust_h)=="Cluster_504"|names(clust_h)=="Cluster_503"|
            names(clust_h)=="Cluster_142"|names(clust_h)=="Cluster_15"|
            names(clust_h)=="Cluster_390"|names(clust_h)=="Cluster_218"|
            names(clust_h)=="Cluster_555"|names(clust_h)=="Cluster_352"|
            names(clust_h)=="Cluster_538"|names(clust_h)=="Cluster_532"|
            names(clust_h)=="Cluster_88"|names(clust_h)=="Cluster_361"|
            names(clust_h)=="Cluster_31"]

cluster_to_species <-
  data.frame(Cluster = c("Cluster_477","Cluster_486","Cluster_504","Cluster_503",
                        "Cluster_142","Cluster_15","Cluster_390","Cluster_218",
                        "Cluster_88", "Cluster_31"),
             Species_name = c("P. copri","P. stercorea","P. vulgatus",
                             "B. thetaiotaomicron","Faecalibacterium",
                             "B. wexlerae","Bifidobacterium",
                             "Christensenellaceae","Oscillibacter", "C. catus"))

envdat <- envdat[,names(envdat) %in% cluster_to_species$Cluster]
names(envdat) <- cluster_to_species$Species_name[match(
  names(envdat), cluster_to_species$Cluster)]

clusters <- as.data.frame(names(clust_h)) %>%
  setNames("Cluster") %>%
  mutate(Species_name = .$Cluster) %>%
  mutate(Species_name = cluster_to_species$Species_name[match(
    .$Cluster, cluster_to_species$Cluster)]) %>%
  mutate(Species_name = ifelse(is.na(.$Species_name),
                              .$Cluster, .$Species_name))

names(clust_h) <- clusters$Species_name[match(names(clust_h), clusters$Cluster)]

library(vegan)
en <- vegan::envfit(mds, envdat, permutations = 999, na.rm = T)
en_coord_cont = as.data.frame(vegan::scores(en, "vectors")) *
  vegan::ordiArrowMul(en, fill = .38)

en_coord_cont_fig <- cont_coord_fix(en_coord_cont)

p <- ggplot(mds.data, aes(x=X, y=Y)) +
  geom_point(aes(color=Ethnicity), size=.1) +
  stat_ellipse(aes(color=Ethnicity), level = 0.004, size=1.5) +
  scale_color_manual(breaks=names_h,
                     values=colors_h,
                     labels = plotnames_h) +
  geom_segment(aes(x = 0, y = 0, xend = Dim1, yend = Dim2),
               data = en_coord_cont_fig, size =.8, alpha = 0.5,
```

```
                colour = "grey20", arrow = arrow(length = unit(0.2, "cm"))) +
  geom_text(data = en_coord_cont_fig, aes(x = xnew, y = ynew), colour = "black",
            fontface = "italic", label = row.names(en_coord_cont_fig),
            size = 4.3) +
  xlab(paste0('PCo1 [',mds.var.per[1],'%]')) +
  ylab(paste0('PCo2 [',mds.var.per[2],'%]')) +
  guides(fill = guide_legend(override.aes=list(shape=21))) +
  xlim(-0.6, 0.4) +
  scale_y_continuous(limits=c(-0.4, 0.42), breaks = seq(-0.4, 0.4, by = 0.2)) +
  coord_cartesian(ylim = c(-0.4,0.4), clip = 'on') +
  theme_Publication()+
  labs(color='Ethnicity & Migration generation')

p_noleg <- p_noleg <- p + theme(legend.position = "none")

layout <- '
A#
BC'

p_rdp <- wrap_plots(A = p1, B = p_noleg, C = p2, design = layout) +
  plot_layout(heights = c(1,5)) +
  plot_layout(widths = c(5,1)) +
  theme(plot.margin = unit(c(0,0,0.1,0.1), "cm"))
p_rdp
```

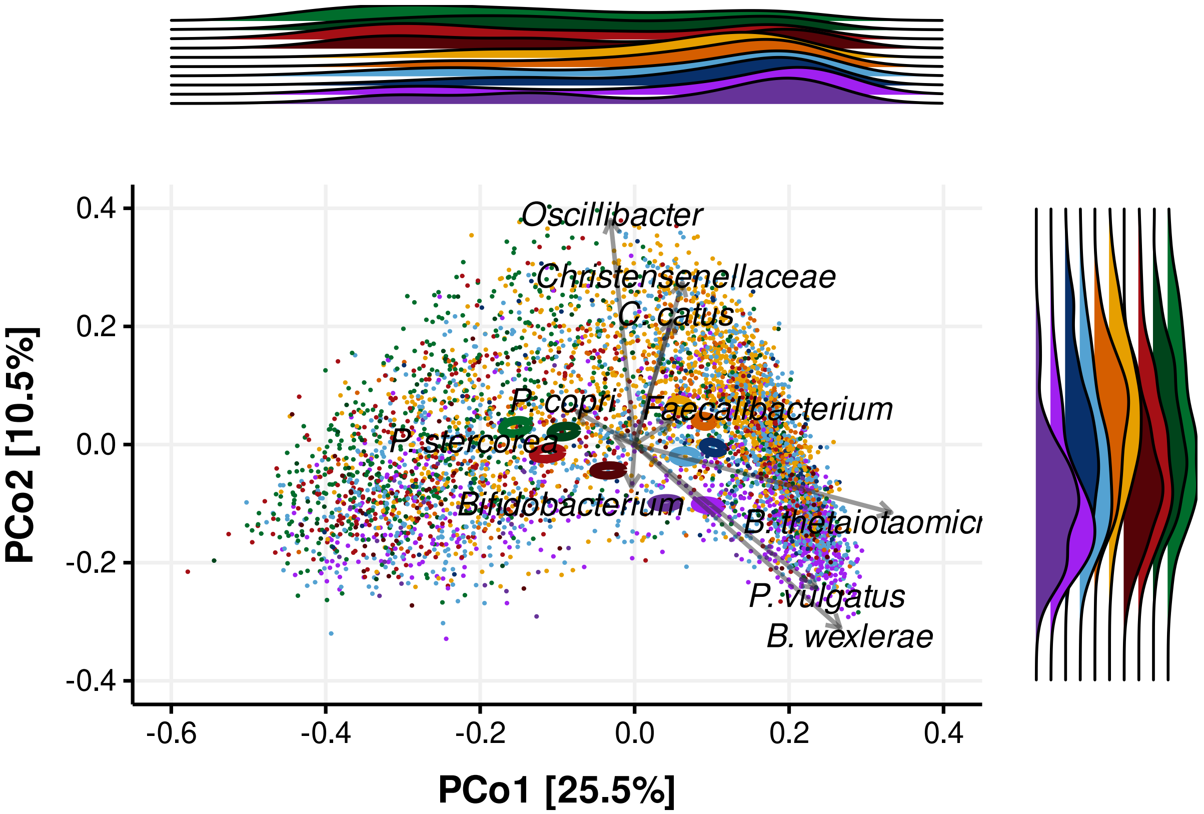


```
as_ggplot(get_legend(p))
```

Ethnicity & Migration generation

- Moroccan G1
- Moroccan G2
- Turkish G1
- Turkish G2
- Dutch (≥ 42)
- Dutch (< 42)
- Af Surinamese G1
- Af Surinamese G2
- SA Surinamese G1
- SA Surinamese G2

Bacteroides / Prevotella ratio

```
asv_h <- as.data.frame(otu_table(ps_h))
taxx <- rio::import("Intermediate_files/taxx_treechecked_BP.csv") %>%
  column_to_rownames("asv_number") %>%
  fix_tax(.) %>%
  mutate(genus_species_asv = sub("0+","", .$genus_species_asv))

names(asv_h) <- taxx$genus_species_asv[match(names(asv_h), rownames(taxx))]
norm_asv_h <- (asv_h / rowSums(asv_h)) *100

taxx_prev <- taxx[taxx$Genus %in% c("Marseilla", "Prevotellamassilia",
                                   "Massiliprevotella", "Metaprevotella",
                                   "Prevotella", "Alloprevotella"),]
asv_h_prev <- norm_asv_h[,names(norm_asv_h) %in%
                           taxx_prev$genus_species_asv] %>%
  rowSums(.)

taxx_bac <- taxx[taxx$Genus %in% c("Bacteroides","Phocaeicola"),]
asv_h_bac <- norm_asv_h[,names(norm_asv_h) %in% taxx_bac$genus_species_asv] %>%
  rowSums(.)

BP_r_h <- as.data.frame(log10(asv_h_bac / asv_h_prev))
names(BP_r_h) <- "BP_ratio"
BP_r_h$group <- dat_h$etn_migrgen[match(rownames(BP_r_h), dat_h$Heliusnr)]
BP_r_h <- BP_r_h[!is.infinite(BP_r_h$BP_ratio),]

BP_r_h_mo <- BP_r_h[BP_r_h$group=="Moroccan G1"|BP_r_h$group=="Moroccan G2",]
BP_r_h_tu <- BP_r_h[BP_r_h$group=="Turkish G1"|BP_r_h$group=="Turkish G2",]
BP_r_h_sasu <- BP_r_h[BP_r_h$group=="SA Surinamese G1"|
                        BP_r_h$group=="SA Surinamese G2",]
BP_r_h_as <- BP_r_h[BP_r_h$group=="Af Surinamese G1"|
                      BP_r_h$group=="Af Surinamese G2",]
BP_r_h_mo1 <- BP_r_h[BP_r_h$group=="Moroccan G1"|
                       BP_r_h$group=="Dutch (Mean age = 57.2)",]
BP_r_h_mo2 <- BP_r_h[BP_r_h$group=="Moroccan G2"|
```

```r
                       BP_r_h$group=="Dutch (Mean age = 32.4)",]
BP_r_h_tu1 <- BP_r_h[BP_r_h$group=="Turkish G1"|
                       BP_r_h$group=="Dutch (Mean age = 57.2)",]
BP_r_h_tu2 <- BP_r_h[BP_r_h$group=="Turkish G2"|
                       BP_r_h$group=="Dutch (Mean age = 32.4)",]
BP_r_h_as1 <- BP_r_h[BP_r_h$group=="Af Surinamese G1"|
                       BP_r_h$group=="Dutch (Mean age = 57.2)",]
BP_r_h_as2 <- BP_r_h[BP_r_h$group=="Af Surinamese G2"|
                       BP_r_h$group=="Dutch (Mean age = 32.4)",]
BP_r_h_sasu1 <- BP_r_h[BP_r_h$group=="SA Surinamese G1"|
                         BP_r_h$group=="Dutch (Mean age = 57.2)",]
BP_r_h_sasu2 <- BP_r_h[BP_r_h$group=="SA Surinamese G2"|
                         BP_r_h$group=="Dutch (Mean age = 32.4)",]
BP_r_h_dutch <- BP_r_h[BP_r_h$group=="Dutch (Mean age = 57.2)"|
                         BP_r_h$group=="Dutch (Mean age = 32.4)",]
fae_sig_list <- list(BP_r_h_as, BP_r_h_as1, BP_r_h_as2,
                     BP_r_h_mo, BP_r_h_mo1, BP_r_h_mo2,
                     BP_r_h_sasu, BP_r_h_sasu1, BP_r_h_sasu2,
                     BP_r_h_tu, BP_r_h_tu1, BP_r_h_tu2,
                     BP_r_h_dutch)

sig_list <- vector(mode = "list", length = length(fae_sig_list))
sig_list_star <- sig_list
for (i in 1:length(fae_sig_list)){
  sig_list[[i]] <- apply(fae_sig_list[[i]][1:(ncol(fae_sig_list[[i]])-1)], 2,
                         function(x) wilcox.test(x ~ fae_sig_list[[i]]$group,
                                                 paired = FALSE)$p.value)
  sig_list_star[[i]] <- ifelse(sig_list[[i]]<=0.001, "***",
                               ifelse(sig_list[[i]]<=0.01, "**",
                                      ifelse(sig_list[[i]]<=0.05, "*", "NS")))
}
sig_list1 <- as.data.frame(sig_list)
sig_list_star[[5]]
```

```
## BP_ratio
##    "***"
```

```r
sig_df <- do.call(rbind.data.frame, sig_list)
names(sig_df) <- "pvalue"

sig_df <- apply(sig_df,2, as.numeric)
sig_df <- as.data.frame(sig_df)
sig_df$Group <- c("BP_r_h_as", "BP_r_h_as1", "BP_r_h_as2",
                  "BP_r_h_mo", "BP_r_h_mo1", "BP_r_h_mo2",
                  "BP_r_h_sasu", "BP_r_h_sasu1", "BP_r_h_sasu2",
                  "BP_r_h_tu", "BP_r_h_tu1", "BP_r_h_tu2",
                  "BP_r_h_dutch")

sig_df$pval.adj <- p.adjust(sig_df$pvalue, method = "fdr")
sig_df$pval.adj.star <- ifelse(sig_df$pval.adj<=0.001, "***",
                               ifelse(sig_df$pval.adj<=0.01, "**",
                                      ifelse(sig_df$pval.adj<=0.05, "*", "NS")))

sig_df
```

```
##          pvalue        Group     pval.adj pval.adj.star
## 1  4.687642e-01    BP_r_h_as 5.078279e-01            NS
## 2  5.452028e-02   BP_r_h_as1 7.875151e-02            NS
## 3  9.244329e-01   BP_r_h_as2 9.244329e-01            NS
## 4  4.144163e-03    BP_r_h_mo 8.269239e-03            **
## 5  3.371833e-64   BP_r_h_mo1 4.383383e-63           ***
## 6  1.049925e-11   BP_r_h_mo2 4.549676e-11           ***
## 7  1.063307e-01  BP_r_h_sasu 1.256636e-01            NS
## 8  3.707730e-04 BP_r_h_sasu1 9.640098e-04           ***
## 9  8.349350e-02 BP_r_h_sasu2 1.085416e-01            NS
## 10 8.200000e-03    BP_r_h_tu 1.332500e-02             *
## 11 4.099661e-28   BP_r_h_tu1 2.664780e-27           ***
## 12 6.875080e-05   BP_r_h_tu2 2.234401e-04           ***
## 13 4.452667e-03 BP_r_h_dutch 8.269239e-03            **
```

```
BP_r_h$group <- factor(BP_r_h$group, levels = c(names_h))

maxpos = 3.2
p1a <- ggplot(BP_r_h, aes(fill=group, y=BP_ratio, x=group)) +
  geom_boxplot(aes(fill=group)) +
  theme_Publication() +
  theme(axis.text.x = element_text(angle = 45, vjust = 1.0, hjust =1.0)) +
  scale_y_continuous(limits = c(-3.2, 5.5),breaks = seq(-2, 4, by = 2))+
  xlab(" ")+
  ylab(expression(~bold("log10(B/P)")))+
  guides(fill = guide_legend(reverse = F)) +
  scale_fill_manual(values = c(colors_h),
                    breaks = c(names_h),
                    labels = c(plotnames_h)) +
  labs(fill="HELIUS") +
  scale_x_discrete(breaks = c(names_h),
                   labels = c(plotnames_h)) +
  geom_signif(
    y_position = c(maxpos, maxpos, maxpos, maxpos+1,maxpos+0.5, maxpos+1.5,
                   maxpos+1, maxpos+2),
    xmin = c(1.1, 3.1, 5.1, 1.1, 3.1, 2.1, 8.9, 4.1),
    xmax = c(1.9, 3.9, 5.9, 4.9, 4.9, 5.9, 5.1, 5.9),
    annotation = c(sig_df[4,4], sig_df[10,4], sig_df[13,4], sig_df[5,4],
                   sig_df[11,4],sig_df[6,4], sig_df[8,4], sig_df[12,4]),
    tip_length = 0.01)
```

BP ratio USI cohort

```
dat_usi <- rio::import("USI_metadata/metadata_US_Immigrants.tsv")
filenames_usi <- rio::import(
  "USI_metadata/filereport_read_run_PRJEB28687_tsv.txt") %>%
  dplyr::select(run_accession, sample_alias)
dat_usi <- merge(filenames_usi, dat_usi, by.x = "sample_alias",
                 by.y = "#SampleID")
asv_usi <- data.frame(otu_table(ps_usi))

dat <- data.frame(sample_data(ps_usi))
USI <- rownames(dat[grep("ERR", rownames(dat)), ])
ps <- prune_samples(sample_names(ps_usi) %in% USI, ps_usi)
```

```
dat_usi <- dat_usi[dat_usi$run_accession %in% rownames(dat), ]
asv_usi_notinmet <- asv_usi[!rownames(asv_usi) %in% dat_usi$run_accession,]
asv_usi <- asv_usi[!rownames(asv_usi) %in% rownames(asv_usi_notinmet),]

dat_usi_omitted <- dat_usi[!dat_usi$sample_alias %in% dat_usi$sample_alias,]
dat_usi_karen <- dat_usi[dat_usi$Sample.Group=="Karen1st"|
                           dat_usi$Sample.Group=="KarenThai",]
dat_usi <- dat_usi[!dat_usi$sample_alias %in% dat_usi_karen$sample_alias,]
dat_usi$Sample.Group[dat_usi$Sample.Group=="Control"] <- "US Control"

names(asv_usi) <- taxx$genus_species_asv[match(names(asv_usi), rownames(taxx))]
asv_usi <- asv_usi[rownames(asv_usi) %in% dat_usi$run_accession,]

dat_usi_control <- dat_usi[dat_usi$Sample.Group=="US Control",]
dat_usi_HT <- dat_usi[dat_usi$Sample.Group=="HmongThai",]
dat_usi_H1 <- dat_usi[dat_usi$Sample.Group=="Hmong1st",]
dat_usi_H2 <- dat_usi[dat_usi$Sample.Group=="Hmong2nd",]

norm_asv_usi <- asv_usi

asv_prev_usi <- norm_asv_usi[,names(norm_asv_usi) %in%
                               taxx_prev$genus_species_asv]
asv_prev_usi <- rowSums(asv_prev_usi)

asv_bac_usi <- asv_usi[,names(asv_usi) %in% taxx_bac$genus_species_asv]
asv_bac_usi <- rowSums(asv_bac_usi)

BP_r_u <- as.data.frame(log10(asv_bac_usi / asv_prev_usi))
names(BP_r_u) <- "BP_ratio"
BP_r_u$group <- dat_usi$Sample.Group[match(rownames(BP_r_u),
                                          dat_usi$run_accession)]
BP_r_u <- BP_r_u[!is.infinite(BP_r_u$BP_ratio),]

BP_r_u_c2 <- BP_r_u[BP_r_u$group=="US Control"|BP_r_u$group=="Hmong2nd",]
BP_r_u_c1 <- BP_r_u[BP_r_u$group=="US Control"|BP_r_u$group=="Hmong1st",]
BP_r_u_ct <- BP_r_u[BP_r_u$group=="US Control"|BP_r_u$group=="HmongThai",]
BP_r_u_21 <- BP_r_u[BP_r_u$group=="Hmong2nd"|BP_r_u$group=="Hmong1st",]
BP_r_u_2t <- BP_r_u[BP_r_u$group=="Hmong2nd"|BP_r_u$group=="HmongThai",]
BP_r_u_1t <- BP_r_u[BP_r_u$group=="Hmong1st"|BP_r_u$group=="HmongThai",]

fae_sig_list_usi <- list(BP_r_u_c2,BP_r_u_c1,BP_r_u_ct,
                         BP_r_u_21,BP_r_u_2t,BP_r_u_1t)

sig_list_usi <- vector(mode = "list", length = length(fae_sig_list))
sig_list_star_usi <- sig_list_usi
for (i in 1:length(fae_sig_list_usi)){
  sig_list_usi[[i]] <- apply(
    fae_sig_list_usi[[i]][1:(ncol(fae_sig_list_usi[[i]])-1)], 2,
    function(x) wilcox.test(x ~ fae_sig_list_usi[[i]]$group, paired = FALSE,
                            exact = F)$p.value)
  sig_list_star_usi[[i]] <- ifelse(sig_list_usi[[i]]<=0.001, "***",
                                   ifelse(sig_list_usi[[i]]<=0.01, "**",
                                          ifelse(sig_list_usi[[i]]<=0.05, "*",
```

```
                                                 "NS")))
}

sig_df_usi <- do.call(rbind.data.frame, sig_list_usi)
names(sig_df_usi) <- "pvalue"

sig_df_usi <- apply(sig_df_usi,2, as.numeric)
sig_df_usi <- as.data.frame(sig_df_usi)
sig_df_usi$Group <- c("BP_r_u_c2","BP_r_u_c1","BP_r_u_ct",
                      "BP_r_u_21","BP_r_u_2t","BP_r_u_1t")

sig_df_usi$pval.adj <- p.adjust(sig_df_usi$pvalue, method = "fdr")
sig_df_usi$pval.adj.star <- ifelse(sig_df_usi$pval.adj<=0.001, "***",
                                   ifelse(sig_df_usi$pval.adj<=0.01, "**",
                                          ifelse(sig_df_usi$pval.adj<=0.05, "*",
                                                 "NS")))

BP_r_u$group <- factor(BP_r_u$group, levels = c("HmongThai", "Hmong1st",
                                                "Hmong2nd","US Control"))
maxpos = 3.5
p2a <- ggplot(BP_r_u, aes(fill=group, y=BP_ratio, x=group)) +
  geom_boxplot(aes(fill=group)) +
  theme_Publication() +
  theme(axis.text.x = element_text(angle = 45, vjust = 1.0, hjust =1.0)) +
  scale_y_continuous(limits = c(-3.2, 5.5),breaks = seq(-2, 4, by = 2))+
  xlab(" ")+
  ylab(expression(~bold("log10(B/P)")))+
  guides(fill = guide_legend(reverse = F)) +
  scale_fill_viridis(option = "E", discrete = T, begin = 0.7, end = 0.0) +
  labs(fill="USI") +
  geom_signif(
    y_position = c(maxpos+0.5, maxpos+0.5, maxpos+1.5, maxpos+1, maxpos+2),
    xmin = c(1.05, 3.05, 1.05, 2.05, 1.05),
    xmax = c(1.95, 3.95, 2.95,3.95, 3.95),
    annotation = c(sig_df_usi[6,4],sig_df_usi[1,4],sig_df_usi[5,4],
                   sig_df_usi[2,4],sig_df_usi[3,4]),
    tip_length = 0.01)

p_obj <- p1a + p2a + plot_layout(widths = c(2.5, 1)) +
  xlab(label= "Ethnicity & Migration generation
                       ") +
  plot_layout(guides = "collect")
p_obj[[2]] = p_obj[[2]] + theme(axis.text.y = element_blank(),
                                axis.ticks.y = element_blank(),
                                axis.title.y = element_blank())
p_obj
```

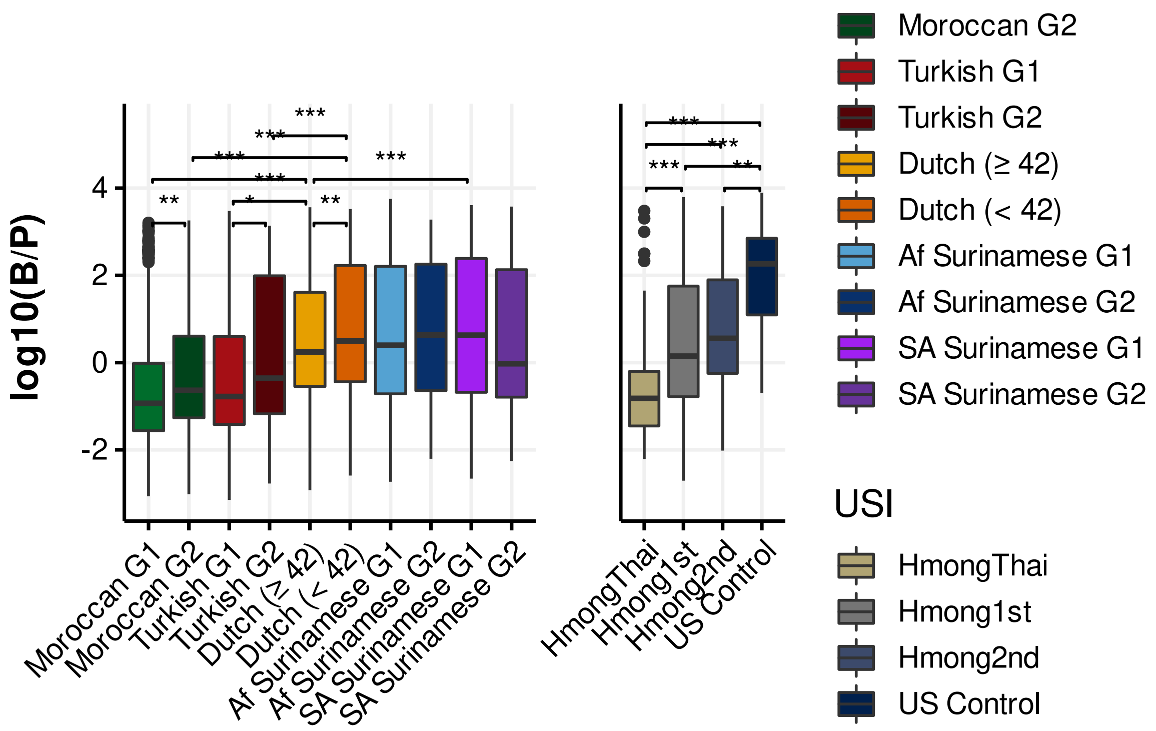

Ethnicity & Migration generation

Machine learning results visualisation

```
as <- rio::import("ML_results/as/feat_imp_gini.txt")
xtrain_as <- rio::import("ML_results/as/X_train_AS.xlsx")
sasu <- rio::import("ML_results/sasu/feat_imp_gini.txt")
xtrain_sasu <- rio::import("ML_results/sasu/X_train_SASU.xlsx")
tu <- rio::import("ML_results/tu/feat_imp_gini.txt")
xtrain_tu <- rio::import("ML_results/tu/X_train_TU.xlsx")
mo <- rio::import("ML_results/mo/feat_imp_gini.txt")
xtrain_mo <- rio::import("ML_results/mo/X_train_MO.xlsx")
du <- rio::import("ML_results/du/feat_imp_gini.txt")
xtrain_du <- rio::import("ML_results/du/X_train_Du.xlsx")

my_list <- list(as, sasu, tu, mo, du)
for (i in 1:length(my_list)){
  my_list[[i]] <- my_list[[i]][order(as.numeric(-my_list[[i]]$RelFeatImp)),]
  my_list[[i]]$names_full <- taxx$genus_species_asv[match(my_list[[i]]$FeatName,
                                                          rownames(taxx))]
}

for (i in 1:length(my_list)){
  my_list[[i]]$ASV_ID <- gsub("_"," ", my_list[[i]]$FeatName)
  my_list[[i]]$ASV_ID <- sub("0+","", my_list[[i]]$ASV_ID)
  my_list[[i]]$blastname <- stables$ASV_name[match(my_list[[i]]$ASV_ID,
                                                   stables$ASV_ID)]
  my_list[[i]]$ASV_ID_bracket <- paste0("(",my_list[[i]]$ASV_ID ,")")
  my_list[[i]]$ASVname <- my_list[[i]]$FeatName
```

```
  my_list[[i]]$FeatName = paste0(my_list[[i]]$blastname, " - ",
                                 my_list[[i]]$ASV_ID_bracket)
}

xtrain_l = list(xtrain_as, xtrain_sasu, xtrain_tu, xtrain_mo, xtrain_du)

for (i in 1:length(xtrain_l)){
  rownames(xtrain_l[[i]]) <- xtrain_l[[i]]$SAMPLE_ID
  xtrain_l[[i]]$SAMPLE_ID <- NULL
  xtrain_l[[i]] <- xtrain_l[[i]][,names(xtrain_l[[i]]) %in%
                                   my_list[[i]]$ASVname]
  names(xtrain_l[[i]]) <- my_list[[i]]$FeatName[match(names(xtrain_l[[i]]),
                                                     my_list[[i]]$ASVname)]
  xtrain_l[[i]]$SAMPLE_ID <- rownames(xtrain_l[[i]])
}

mo <- my_list[[4]] %>% head(20) %>%  select(FeatName, ASV_ID)
tu <- my_list[[3]] %>% head(20) %>%  select(FeatName, ASV_ID)
as <- my_list[[1]] %>% head(20) %>%  select(FeatName, ASV_ID)
sasu <- my_list[[2]] %>% head(20) %>%  select(FeatName, ASV_ID)
du <- my_list[[5]] %>% head(20) %>%  select(FeatName, ASV_ID)

df <- rbind(as, mo, tu, du, sasu) %>%
  distinct(.) %>%
  mutate(Moroccan = ifelse(.$FeatName %in% mo$FeatName, 1, 0),
         Turkish = ifelse(.$FeatName %in% tu$FeatName, 1, 0),
         Dutch = ifelse(.$FeatName %in% du$FeatName, 1, 0),
         `African Surinamese` =ifelse(.$FeatName %in% as$FeatName, 1, 0),
         `South-Asian Surinamese` =
           ifelse(.$FeatName %in% sasu$FeatName, 1, 0)) %>%
  mutate(SUM = c(Moroccan + Turkish + Dutch + `African Surinamese` +
                   `South-Asian Surinamese`)) %>%
  arrange(-SUM) %>%
  mutate(ASV_sequence = stables$ASV_sequence[match(.$ASV_ID,
                                                   stables$ASV_ID)]) %>%
  mutate(FeatName = stables$ASV_name[match(.$ASV_ID, stables$ASV_ID)]) %>%
  mutate(ASV_name = paste(.$FeatName, .$ASV_ID, sep=" - "))

df[1:8,1:6]
```

```
##                      FeatName  ASV_ID Moroccan Turkish Dutch African Surinamese
## 1             Faecalibacterium   ASV 2        1       1     0                  1
## 2 Lachnospiraceae MH699349.1  ASV 42        1       0     1                  1
## 3       Blautia massiliensis   ASV 3        1       0     0                  1
## 4         Romboutsia ilealis  ASV 29        0       0     0                  1
## 5           Faecalibacterium  ASV 18        0       0     0                  1
## 6        Anaerostipes hadrus   ASV 8        0       0     1                  1
## 7      Dorea formicigenerans   ASV 7        0       1     0                  1
## 8        Blautia phocaeensis ASV 134        1       0     0                  1
```

```
df_g <- df %>% select(-ASV_ID, -ASV_name) %>% gather("data","present", 2:6) %>%
  filter(present>0)
library(UpSetR)
```

```r
x = list(df_g %>% filter(data == "Moroccan") %>%
           dplyr::select(FeatName) %>% unlist(),
         df_g %>% filter(data == "Turkish") %>%
           dplyr::select(FeatName) %>% unlist(),
         df_g %>% filter(data == "Dutch") %>%
           dplyr::select(FeatName) %>% unlist(),
         df_g %>% filter(data == "African Surinamese") %>%
           dplyr::select(FeatName) %>% unlist(),
         df_g %>% filter(data == "South-Asian Surinamese") %>%
           dplyr::select(FeatName) %>% unlist())

names(x) <- c("Moroccan", "Turkish", "Dutch", "African Surinamese",
              "South-Asian Surinamese")

p_up <- upset(df, set_size.show = FALSE, order.by = "freq",show.numbers = F,
              att.pos = "bottom", main.bar.color = "black", queries =
                list(list(query = intersects,
                          params = list("Moroccan", "Turkish"), active = T,
                          color = "purple"),
                     list(query = intersects, params =
                            list("Moroccan", "Turkish", "African Surinamese",
                                 "South-Asian Surinamese"), active = T,
                          color = "orange")),
      text.scale = c(1.5,1.5,1, 1, 1.5, 1))
p_up
```

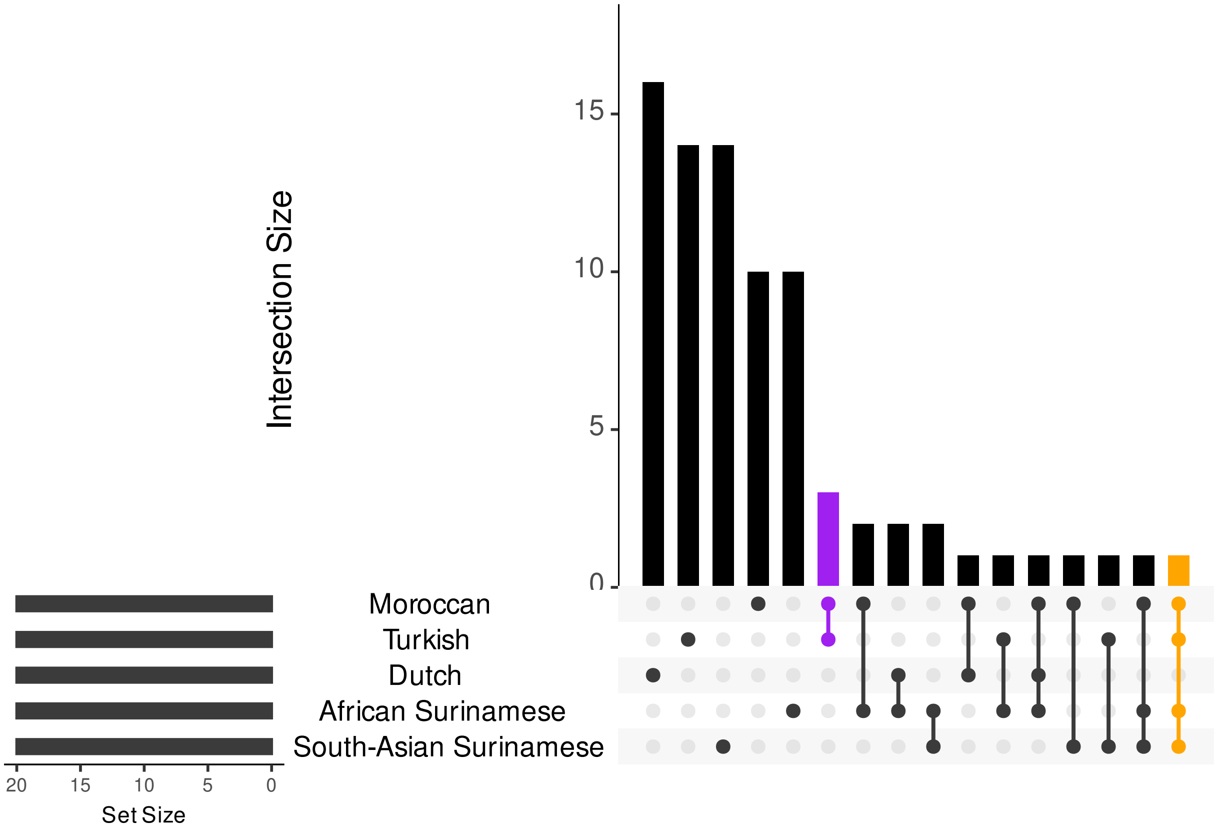

```
df_mo_tu <- df[df$Moroccan==1 & df$Turkish==1 & df$`African Surinamese`==0 &
                 df$`South-Asian Surinamese`==0,]
df_mo_tu$ASV_name <- sub("//).*","",sub(".*\\(","",sub(")","",
                                                       df_mo_tu$FeatName)))
df_mo_tu$name <- paste0(df_mo_tu$FeatName, " - ", df_mo_tu$ASV_ID)
ps_helius_mo_tu <- ps_h %>%
  subset_samples(., !H1_EtnTotaal=="South-Asian Surinamese" &
                   !H1_EtnTotaal=="African Surinamese" & !H1_EtnTotaal=="Dutch")
dat_mo_tu <- ps_helius_mo_tu@sam_data
asv_mo_tu <- as.data.frame(ps_helius_mo_tu@otu_table)
names(asv_mo_tu) <- gsub("_"," ", sub("0+","", names(asv_mo_tu)))
asv_mo_tu <- (asv_mo_tu/rowSums(asv_mo_tu)*100)
asv_mo_tu <- asv_mo_tu[,names(asv_mo_tu) %in% df_mo_tu$ASV_ID]
names(asv_mo_tu) <- df_mo_tu$name[match(names(asv_mo_tu), df_mo_tu$ASV_ID)]

dat_mo_tu1 <- merge(asv_mo_tu, dat_mo_tu, by = "row.names") %>%
  dplyr::select(1,3,4,2, H1_EtnTotaal, H1_MigrGeneratie) %>%
  tibble::column_to_rownames("Row.names") %>%
  dplyr::mutate(etn_migrgen = paste(H1_EtnTotaal,
                                    H1_MigrGeneratie, sep=" G")) %>%
  dplyr::mutate(etn_migrgen = as.factor(etn_migrgen))

comparisons <- list(c("Moroccan G1","Moroccan G2"), c("Turkish G1",
                                                      "Turkish G2"))

ggplot(dat_mo_tu1, aes(fill=etn_migrgen, y=`Blautia faecis - ASV 6`,
                             x=etn_migrgen)) +
  geom_violin(aes(fill=etn_migrgen)) +
  theme_Publication() +
  theme(axis.text.x = element_text(angle = 45, vjust = 1.0, hjust =1.0)) +
  labs(y = expression(paste(italic("Blautia faecis"), " - (ASV 6)")))+

  guides(fill = guide_legend(reverse = F)) +
  scale_fill_manual(values = c("#006D2C","#00441B","#A50F15", "#550307"),
                    labels = c("Moroccan G1", "Moroccan G2",
                               "Turkish G1","Turkish G2")) +
  theme(legend.position = "none") +
  stat_compare_means(comparisons = comparisons,label = "p.signif",
                     method = "wilcox.test",
                     paired=F, tip.length = .02, label.y = 4,
                     symnum.args = list(cutpoints = c(0, 0.001, 0.01, 0.05, 1),
                                        symbols = c("***", "**", "*", "ns"))) +
  theme(axis.text=element_text(size=10),
        axis.title=element_text(size=12,face="bold"),
        axis.title.x = element_blank())
```

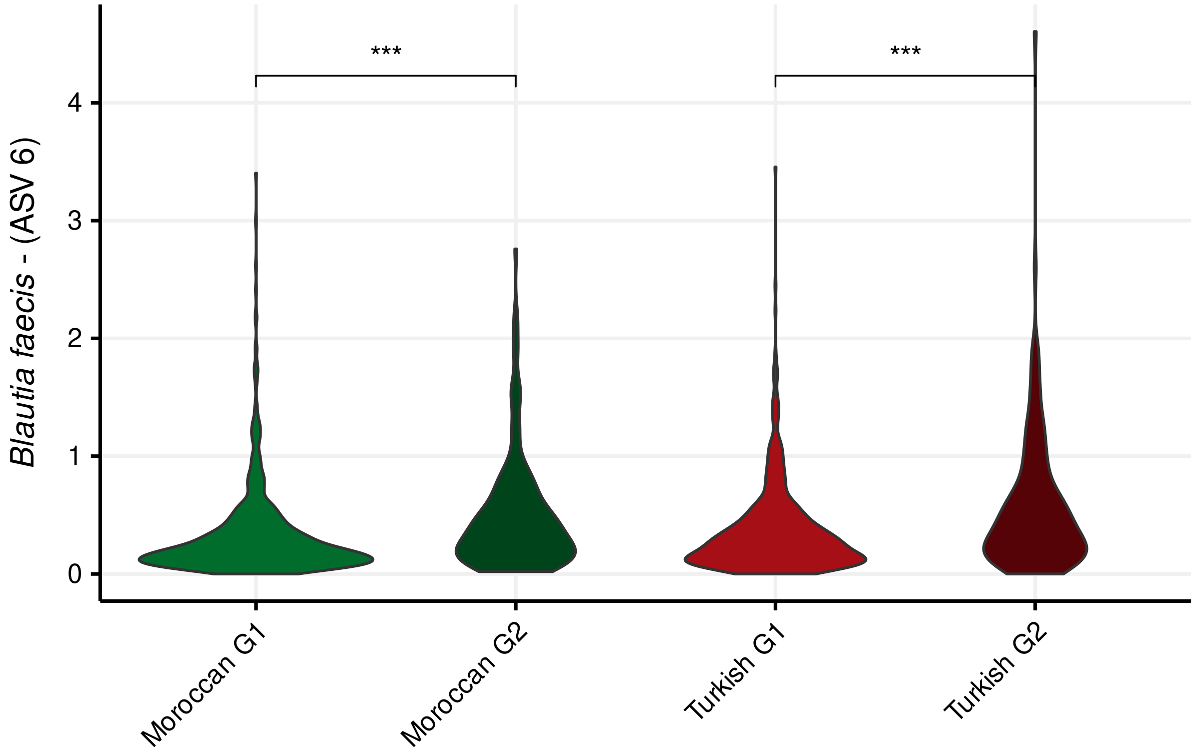


```r
ggplot(dat_mo_tu1, aes(fill=etn_migrgen,
                             y=`Mediterraneibacter faecis - ASV 22`,
                             x=etn_migrgen)) +
  geom_violin(aes(fill=etn_migrgen)) +
  theme_Publication() +
  theme(axis.text.x = element_text(angle = 45, vjust = 1.0, hjust =1.0)) +
  scale_y_continuous(breaks = seq(0, 5, by = 1))+
  labs(x = "Ethnicity & Migration generation",
       y = expression(paste(italic("Ruminococcus faecis"), " - (ASV 22)"))) +
  guides(fill = guide_legend(reverse = F)) +
  scale_fill_manual(values = c("#006D2C","#00441B","#A50F15", "#550307"),
                    labels = c("Moroccan G1", "Moroccan G2",
                               "Turkish G1","Turkish G2")) +
  theme(legend.position = "none") +
  stat_compare_means(comparisons = comparisons,label = "p.signif",
                     method = "wilcox.test",
                     paired=F, tip.length = .02, label.y = 5,
                     symnum.args = list(cutpoints = c(0, 0.001, 0.01, 0.05, 1),
                                        symbols = c("***", "**", "*", "ns"))) +
  theme(axis.text=element_text(size=10),
        axis.title=element_text(size=12,face="bold"))
```

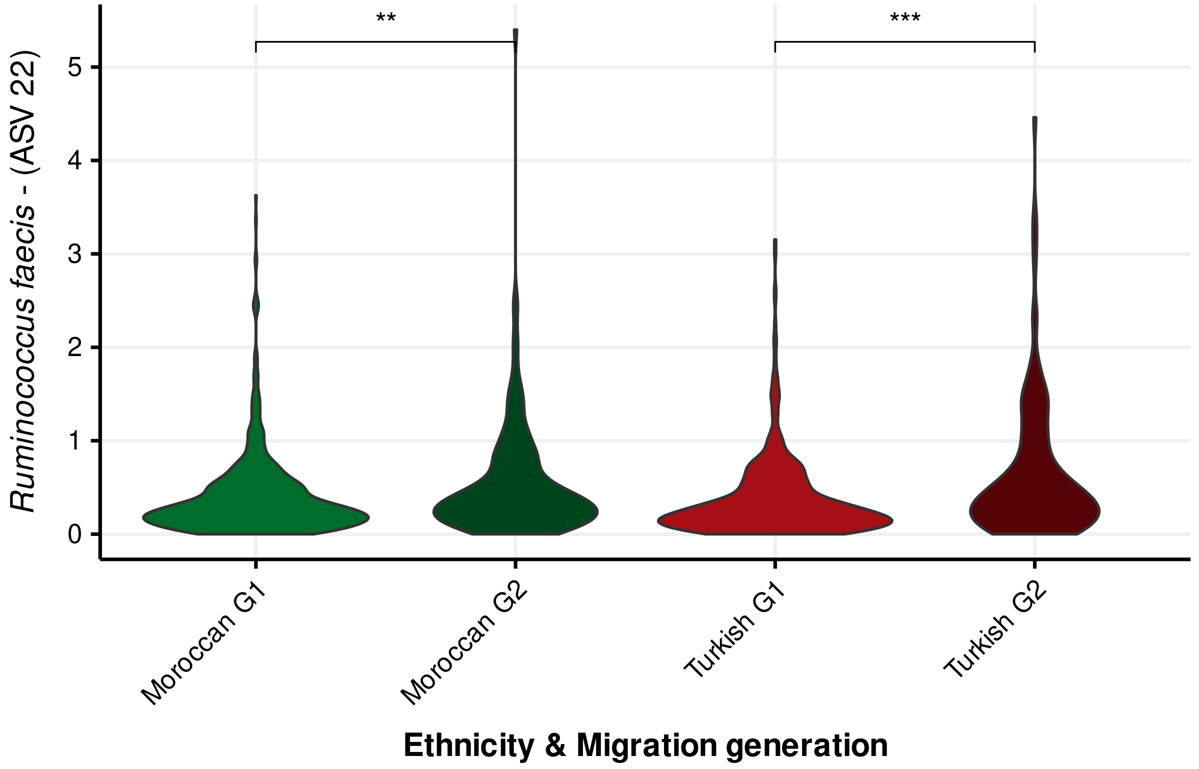


```
ggplot(dat_mo_tu1, aes(fill=etn_migrgen,
                             y=`Lachnospiraceae LC515621.1 - ASV 65`,
                             x=etn_migrgen)) +
  geom_violin(aes(fill=etn_migrgen)) +
  theme_Publication() +
  theme(axis.text.x = element_text(angle = 45, vjust = 1.0, hjust =1.0)) +
  labs(x = "Ethnicity & Migration generation",
       y = expression(paste("Lachnospiraceae", "  LC515621.1 - (ASV 65)")))+
  guides(fill = guide_legend(reverse = F)) +
  scale_fill_manual(values = c("#006D2C","#00441B","#A50F15", "#550307"),
                    labels = c("Moroccan G1", "Moroccan G2",
                               "Turkish G1","Turkish G2")) +
  labs(fill="Ethnicity & Migration generation") +
  stat_compare_means(comparisons = comparisons,label = "p.signif",
                     method = "wilcox.test",
                     paired=F, tip.length = .02, label.y = 0.6,
                     symnum.args = list(cutpoints = c(0, 0.001, 0.01, 0.05, 1),
                                        symbols = c("***", "**", "*", "ns"))) +
  theme(axis.text=element_text(size=10),
        axis.title=element_text(size=12,face="bold"),
        axis.title.x = element_blank())
```

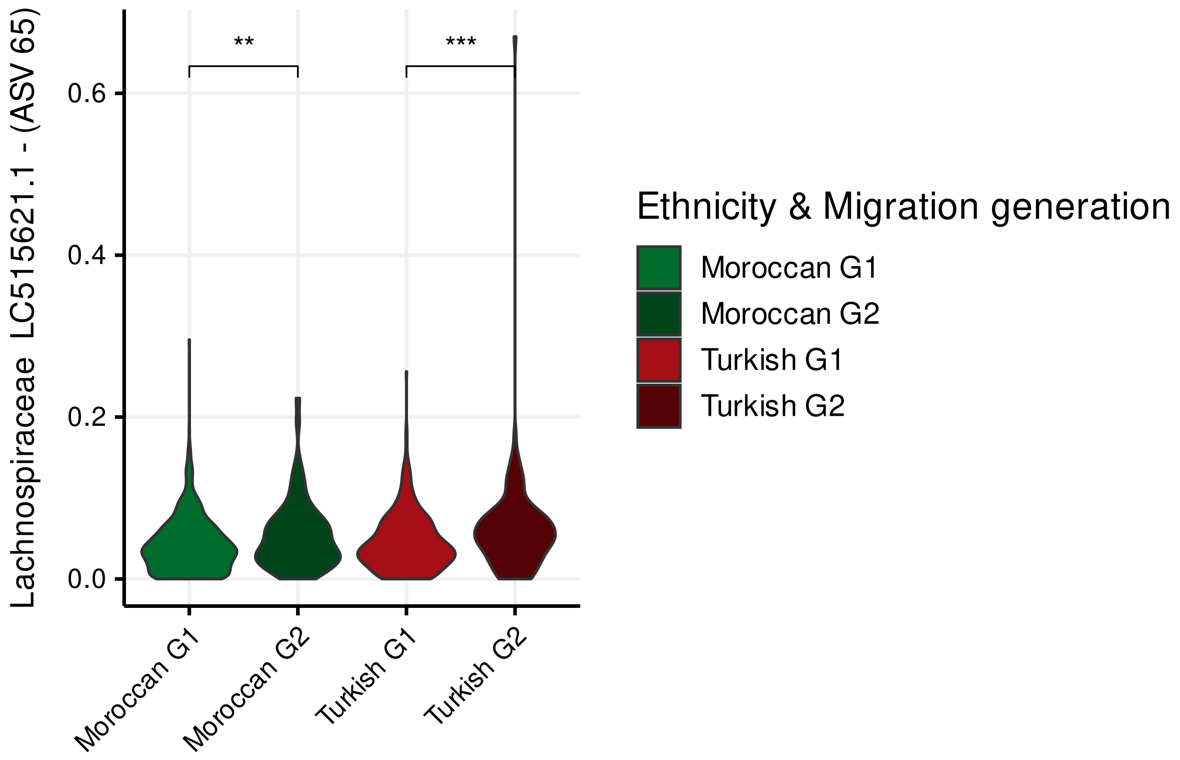

Faecalibacterium ASV 2 visualisation

```
asv_2 <- norm_asv_h %>% select(`Faecalibacterium - (ASV 2)`) %>%
  mutate(group = dat_h$etn_migrgen[match(rownames(.),dat_h$Heliusnr)])

asv_2_mo <-
  asv_2[asv_2$group=="Moroccan G1"|asv_2$group=="Moroccan G2",]
asv_2_tu <-
  asv_2[asv_2$group=="Turkish G1"|asv_2$group=="Turkish G2",]
asv_2_sasu <-
  asv_2[asv_2$group=="SA Surinamese G1"|asv_2$group=="SA Surinamese G2",]
asv_2_as <-
  asv_2[asv_2$group=="Af Surinamese G1"|asv_2$group=="Af Surinamese G2",]
asv_2_mo1 <-
  asv_2[asv_2$group=="Moroccan G1"|asv_2$group=="Dutch (Mean age = 57.2)",]
asv_2_mo2 <-
  asv_2[asv_2$group=="Moroccan G2"|asv_2$group=="Dutch (Mean age = 32.4)",]
asv_2_tu1 <-
  asv_2[asv_2$group=="Turkish G1"|asv_2$group=="Dutch (Mean age = 57.2)",]
asv_2_tu2 <-
  asv_2[asv_2$group=="Turkish G2"|asv_2$group=="Dutch (Mean age = 32.4)",]
asv_2_as1 <-
  asv_2[asv_2$group=="Af Surinamese G1"|asv_2$group=="Dutch (Mean age = 57.2)",]
asv_2_as2 <-
  asv_2[asv_2$group=="Af Surinamese G2"|asv_2$group=="Dutch (Mean age = 32.4)",]
asv_2_sasu1 <-
  asv_2[asv_2$group=="SA Surinamese G1"|asv_2$group=="Dutch (Mean age = 57.2)",]
asv_2_sasu2 <-
  asv_2[asv_2$group=="SA Surinamese G2"|asv_2$group=="Dutch (Mean age = 32.4)",]
```

```r
asv_2_dutch <-
  asv_2[asv_2$group=="Dutch (Mean age = 57.2)"|
          asv_2$group=="Dutch (Mean age = 32.4)",]

fae_sig_list <- list(asv_2_as, asv_2_as1, asv_2_as2, asv_2_mo, asv_2_mo1,
                     asv_2_mo2, asv_2_sasu, asv_2_sasu1, asv_2_sasu2, asv_2_tu,
                     asv_2_tu1, asv_2_tu2, asv_2_dutch)
sig_list <- vector(mode = "list", length = length(fae_sig_list))
sig_list_star <- sig_list
for (i in 1:length(fae_sig_list)){
  sig_list[[i]] <- apply(fae_sig_list[[i]][1:(ncol(fae_sig_list[[i]])-1)], 2,
                         function(x) wilcox.test(x ~ fae_sig_list[[i]]$group,
                                                 paired = FALSE)$p.value)
  sig_list_star[[i]] <- ifelse(sig_list[[i]]<=0.001, "***",
                               ifelse(sig_list[[i]]<=0.01, "**",
                                      ifelse(sig_list[[i]]<=0.05, "*", "NS")))
}

sig_df <- do.call(rbind.data.frame, sig_list)
names(sig_df) <- "pvalue"

sig_df <- apply(sig_df,2, as.numeric)
sig_df <- as.data.frame(sig_df)
sig_df$Group <- c("asv_2_as", "asv_2_as1", "asv_2_as2", "asv_2_mo", "asv_2_mo1",
                  "asv_2_mo2","asv_2_sasu", "asv_2_sasu1", "asv_2_sasu2",
                  "asv_2_tu", "asv_2_tu1", "asv_2_tu2", "asv_2_dutch")

sig_df$pval.adj <- p.adjust(sig_df$pvalue, method = "fdr")
sig_df$pval.adj.star <- ifelse(sig_df$pval.adj<=0.001, "***",
                               ifelse(sig_df$pval.adj<=0.01, "**",
                                      ifelse(sig_df$pval.adj<=0.05, "*", "NS")))


asv_2 <- asv_2 %>% group_by(group) %>%
  filter(between(`Faecalibacterium - (ASV 2)`,
                 quantile(`Faecalibacterium - (ASV 2)`, 0.0),
                 quantile(`Faecalibacterium - (ASV 2)`, 0.99))) %>%
    mutate(group = factor(group, levels = c(names_h)))

maxpos = 10
p1 <- ggplot(asv_2, aes(fill=group, y=`Faecalibacterium - (ASV 2)`, x=group)) +
  geom_violin(aes(fill=group)) +
  theme_Publication() +
  theme(axis.text.x = element_text(angle = 45, vjust = 1.0, hjust =1.0)) +
  scale_y_continuous(limits=c(0, 12), breaks = seq(0, 12, by = 4))+
  xlab(" ")+
  ylab(expression(~bolditalic("Faecalibacterium")~bold(" A2-165 (ASV 2)")))+
  guides(fill = guide_legend(reverse = F)) +
  scale_fill_manual(values = c(colors_h),
                    labels = c(plotnames_h)) +
  labs(fill="HELIUS") +
  scale_x_discrete(breaks=c(names_h),
                   labels=c(plotnames_h)) +
```

```
  geom_signif(
    y_position = c(maxpos, maxpos, maxpos, maxpos, maxpos, maxpos+2,maxpos+1),
    xmin = c(1.1, 3.1, 5.1, 7.1, 9.1, 1.1, 3.1),
    xmax = c(1.9, 3.9, 5.9, 7.9, 9.9, 4.9, 4.9),
    annotation = c(sig_df[4,4], sig_df[10,4], sig_df[13,4], sig_df[1,4],
                   sig_df[7,4], sig_df[5,4], sig_df[11,4]),
    tip_length = 0.01)
```

```
asv_2 <- asv_usi %>% select(`Faecalibacterium - (ASV 2)`) %>%
  mutate(group = dat_usi$Sample.Group[match(rownames(.),dat_usi$run_accession)])

asv_2_usi_c2 <- asv_2[asv_2$group=="US Control"|asv_2$group=="Hmong2nd",]
asv_2_usi_c1 <- asv_2[asv_2$group=="US Control"|asv_2$group=="Hmong1st",]
asv_2_usi_ct <- asv_2[asv_2$group=="US Control"|asv_2$group=="HmongThai",]
asv_2_usi_21 <- asv_2[asv_2$group=="Hmong2nd"|asv_2$group=="Hmong1st",]
asv_2_usi_2t <- asv_2[asv_2$group=="Hmong2nd"|asv_2$group=="HmongThai",]
asv_2_usi_1t <- asv_2[asv_2$group=="Hmong1st"|asv_2$group=="HmongThai",]

fae_sig_list_usi <- list(asv_2_usi_c2,asv_2_usi_c1,asv_2_usi_ct,
                         asv_2_usi_21,asv_2_usi_2t,asv_2_usi_1t)

sig_list_usi <- vector(mode = "list", length = length(fae_sig_list))
sig_list_star_usi <- sig_list_usi
for (i in 1:length(fae_sig_list_usi)){
  sig_list_usi[[i]] <-
    apply(fae_sig_list_usi[[i]][1:(ncol(fae_sig_list_usi[[i]])-1)], 2,
          function(x) wilcox.test(x ~ fae_sig_list_usi[[i]]$group,
                                  paired = FALSE)$p.value)
  sig_list_star_usi[[i]] <- ifelse(sig_list_usi[[i]]<=0.001, "***",
                                   ifelse(sig_list_usi[[i]]<=0.01, "**",
                                          ifelse(sig_list_usi[[i]]<=0.05, "*",
                                                 "NS")))
}

sig_df_usi <- do.call(rbind.data.frame, sig_list_usi)
names(sig_df_usi) <- "pvalue"

sig_df_usi <- apply(sig_df_usi,2, as.numeric)
sig_df_usi <- as.data.frame(sig_df_usi)
sig_df_usi$Group <- c("asv_2_usi_c2","asv_2_usi_c1","asv_2_usi_ct",
                      "asv_2_usi_21","asv_2_usi_2t","asv_2_usi_1t")


sig_df_usi$pval.adj <- p.adjust(sig_df_usi$pvalue, method = "fdr")
sig_df_usi$pval.adj.star <- ifelse(sig_df_usi$pval.adj<=0.001, "***",
                                   ifelse(sig_df_usi$pval.adj<=0.01, "**",
                                          ifelse(sig_df_usi$pval.adj<=0.05, "*",
                                                 "NS")))


asv_2p <- asv_2 %>% group_by(group) %>%
  filter(between(`Faecalibacterium - (ASV 2)`,
                 quantile(`Faecalibacterium - (ASV 2)`, 0.0),
                 quantile(`Faecalibacterium - (ASV 2)`, 0.99))) %>%
```

```
  mutate(`Faecalibacterium - (ASV 2)` = `Faecalibacterium - (ASV 2)` * 100) %>%
  mutate(group = factor(group, levels = c("HmongThai", "Hmong1st",
                                          "Hmong2nd","US Control")))

p2 <- ggplot(asv_2p, aes(fill=group, y=`Faecalibacterium - (ASV 2)`, x=group)) +
  geom_violin(aes(fill=group)) +
  scale_fill_viridis(option = "E", discrete = T, begin = 0.7, end = 0.0) +
  theme_Publication() +
  theme(axis.text.x = element_text(angle = 45, vjust = 1.0, hjust =1.0)) +
  scale_y_continuous(limits=c(0, 12), breaks = seq(0, 12, by = 4))+
  xlab(" ") +
  ylab(" ")+
  guides(fill = guide_legend(reverse = F)) +
  labs(fill="USI") +
  geom_signif(
    y_position = c(maxpos-1, maxpos, maxpos+1, maxpos+2),
    xmin = c(2.1, 1.1, 2.1, 1.1),
    xmax = c(2.9, 2.9, 3.9, 3.9),
    annotation = c(sig_df_usi[4,4],sig_df_usi[5,4],sig_df_usi[2,4],
                   sig_df_usi[3,4]),
    tip_length = 0.01)

p1
```

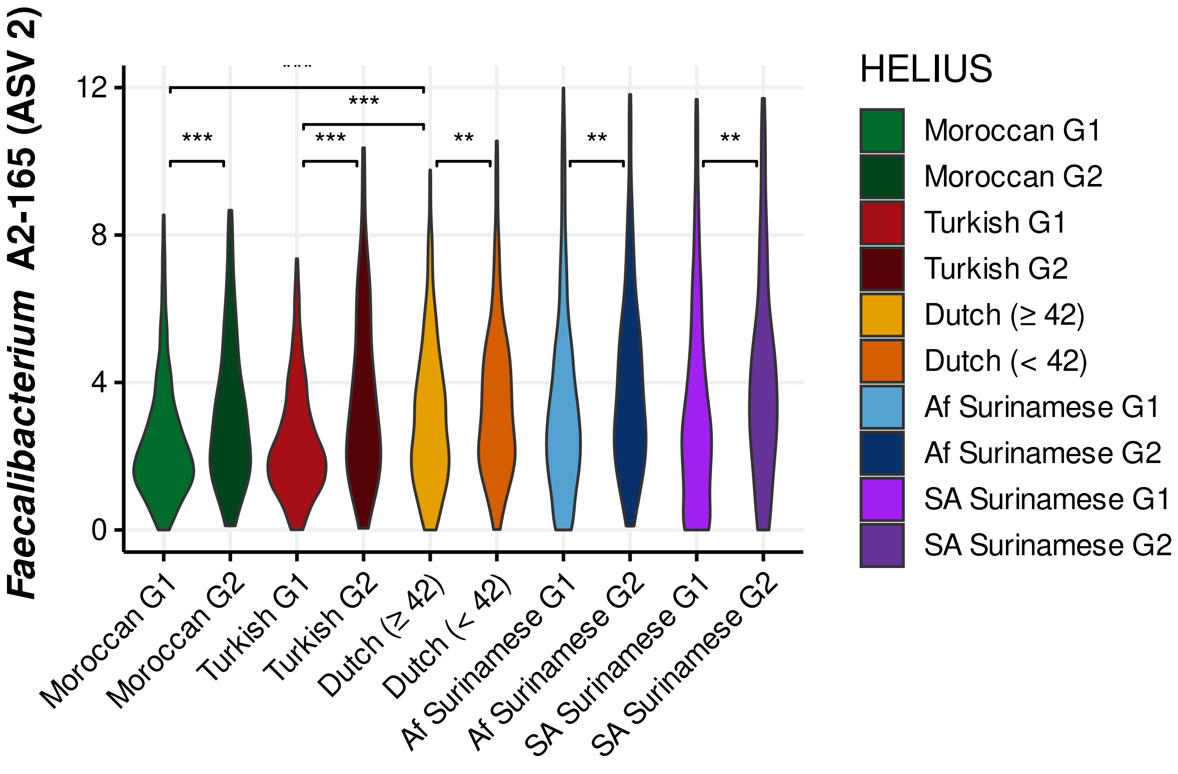


```
p2
```

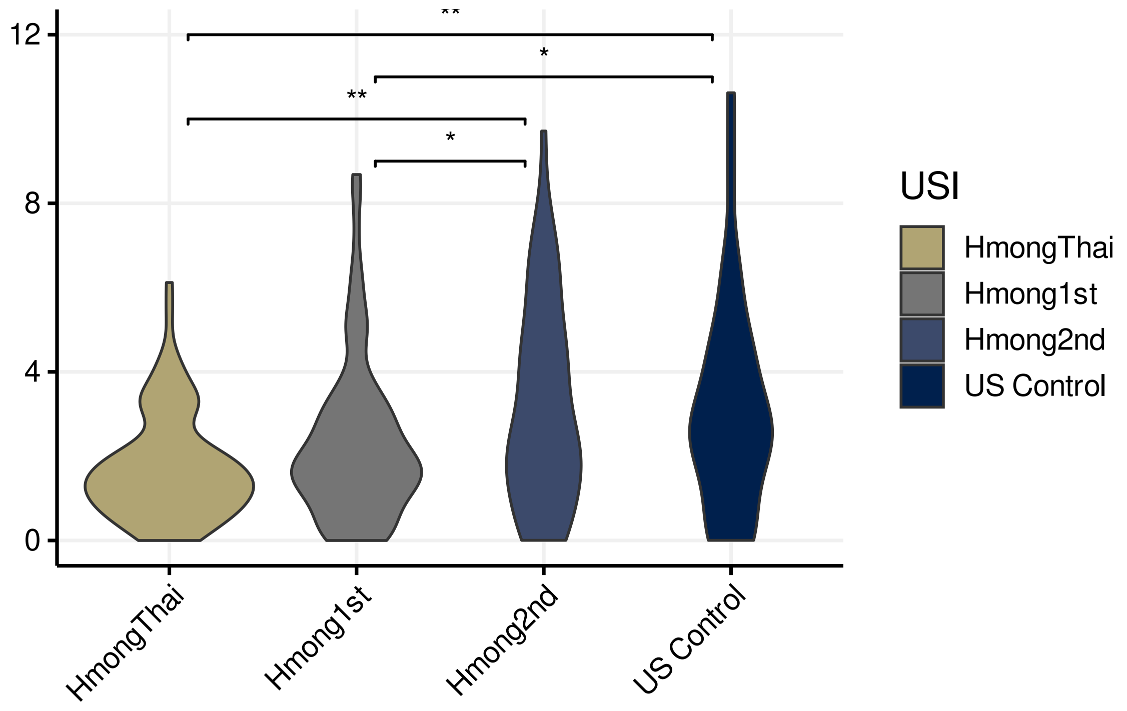

Faecalibacterium phylogenetic tree with heatmap to show if the ASV is higher in the first- or second generation and if the ASV is higher in the Dutch or other ethnicities.

```
clusters <- rio::import("Intermediate_files/clusters_with_tax_h10.csv")
cluster_Faecali_fig <- clusters[
  clusters$Original_ASV=="ASV_00012"|clusters$Original_ASV=="ASV_00150"|
    clusters$Original_ASV=="ASV_00082"|clusters$Original_ASV=="ASV_00200"|
    clusters$Original_ASV=="ASV_00247"|clusters$Original_ASV=="ASV_00387"|
    clusters$Original_ASV=="ASV_00018"|clusters$Original_ASV=="ASV_00020"|
    clusters$Original_ASV=="ASV_00168"|clusters$Original_ASV=="ASV_00038"|
    clusters$Original_ASV=="ASV_00333"|clusters$Original_ASV=="ASV_00014"|
    clusters$Original_ASV=="ASV_00002"|clusters$Original_ASV=="ASV_00211",]
ps_faecali <- subset_taxa(ps_h, rownames(data.frame(tax_table(ps_h))) %in%
                            cluster_Faecali_fig$Original_ASV)

stables$ASV_comb <- paste0(stables$ASV_name, " - (", stables$ASV_ID,")")

ps_helius_tree <- phy_tree(ps_faecali)
ps_helius_tree$tip.label <- stables$ASV_comb[match(
  gsub("_"," ", sub("0+","", ps_helius_tree$tip.label)), stables$ASV_ID)]

taxx_ <- data.frame(tax_table(ps_faecali)) %>%
  mutate(ASV_name_ID = stables$ASV_comb[match(
    gsub("_"," ", sub("0+","", rownames(.))), stables$ASV_ID)]) %>%
  mutate(ASV_ID = rownames(.)) %>%
  mutate(ASV_name = stables$ASV_name[match(
    gsub("_"," ", sub("0+","", rownames(.))), stables$ASV_ID)]) %>%
```

```
  `rownames<-`(.[,7]) %>%
  select(ASV_name, ASV_ID)
taxx_first <- taxx_ %>% select(ASV_name)
asvs <- as.data.frame(ps_faecali@otu_table)

modu <- ASV_labels(asvs = asvs, group1 = dat_h[dat_h$Ethnicity=="Moroccan",],
                   group2 = dat_h[dat_h$Ethnicity=="Dutch",],
                   group_names = c("Moroccan", "Dutch"))
tudu <-ASV_labels(asvs = asvs, group1 = dat_h[dat_h$Ethnicity=="Turkish",],
                  group2 = dat_h[dat_h$Ethnicity=="Dutch",],
                  group_names = c("Turkish", "Dutch"))
asdu <-ASV_labels(asvs = asvs, group1 = dat_h[dat_h$Ethnicity=="Af Surinamese",],
                  group2 = dat_h[dat_h$Ethnicity=="Dutch",],
                  group_names = c("Af Surinamese", "Dutch"))
sasudu <- ASV_labels(asvs = asvs, group1 = dat_h[dat_h$Ethnicity==
                                                  "SA Surinamese",],
                     group2 = dat_h[dat_h$Ethnicity=="Dutch",],
                     group_names = c("SA Surinamese", "Dutch"))

mo12 <- ASV_labels(asvs = asvs,
                   group1 = dat_h[dat_h$etn_migrgen=="Moroccan G1",],
                   group2 = dat_h[dat_h$etn_migrgen=="Moroccan G2",],
                   group_names = c("First generation", "Second generation"))
tu12 <- ASV_labels(asvs = asvs,
                   group1 = dat_h[dat_h$etn_migrgen=="Turkish G1",],
                   group2 = dat_h[dat_h$etn_migrgen=="Turkish G2",],
                   group_names = c("First generation", "Second generation"))
as12 <- ASV_labels(asvs = asvs,
                   group1 = dat_h[dat_h$etn_migrgen=="Af Surinamese G1",],
                   group2 = dat_h[dat_h$etn_migrgen=="Af Surinamese G2",],
                   group_names = c("First generation", "Second generation"))
sasu12 <- ASV_labels(asvs = asvs,
                     group1 = dat_h[dat_h$etn_migrgen=="SA Surinamese G1",],
                     group2 = dat_h[dat_h$etn_migrgen=="SA Surinamese G2",],
                     group_names = c("First generation", "Second generation"))
du12 <- ASV_labels(asvs = asvs,
                   group1 = dat_h[dat_h$etn_migrgen=="Dutch (Mean age = 57.2)",],
                   group2 = dat_h[dat_h$etn_migrgen=="Dutch (Mean age = 32.4)",],
                   group_names = c("First generation", "Second generation"))

taxx_$modu <- modu$label[match(taxx_$ASV_ID, rownames(modu))]
taxx_$tudu <- tudu$label[match(taxx_$ASV_ID, rownames(tudu))]
taxx_$asdu <- asdu$label[match(taxx_$ASV_ID, rownames(asdu))]
taxx_$sasudu <- sasudu$label[match(taxx_$ASV_ID, rownames(sasudu))]
taxx_$du12 <- du12$label[match(taxx_$ASV_ID, rownames(du12))]
taxx_$mo12 <- mo12$label[match(taxx_$ASV_ID, rownames(mo12))]
taxx_$tu12 <- tu12$label[match(taxx_$ASV_ID, rownames(tu12))]
taxx_$as12 <- as12$label[match(taxx_$ASV_ID, rownames(as12))]
taxx_$sasu12 <- sasu12$label[match(taxx_$ASV_ID, rownames(sasu12))]

taxx_1 <- taxx_ %>% select(-ASV_ID)
taxx_1$whitspace <- c(" ")
taxx_1$whitspace2 <- c(" ")
```

```r
taxx_1 %>% select(1,11,2:5,12,6:10) -> taxx_1
taxx_1_c <- taxx_1 %>% select(1)
taxx_2_c <- taxx_1 %>% select(3:6)
taxx_3_c <- taxx_1 %>% select(8:12)

col_lab_2 <- c("Moroccan","Turkish","Af Surinamese","SA Surinamese")
col_lab_3 <- c("Dutch", "Moroccan", "Turkish","Af Surinamese","SA Surinamese")

tax1c <- taxx_1_c
tax2c <- as.data.frame(ifelse(taxx_2_c=="Dutch", "Dutch", "Other ethnicity"))
tax3c <- sub(".*G1.*", "First generation", taxx_3_c, ignore.case = TRUE)
p1 <- ggtree(ps_helius_tree) + geom_tiplab()
p2 <- gheatmap(p1, taxx_1_c, colnames = T, offset=.14, width=.045, font.size=2,
               colnames_angle=45,custom_column_labels = "Taxonomy",
               colnames_offset_x = -0.011, colnames_offset_y = -0.5, hjust=0) +
  scale_fill_manual("Taxonomy",
                    breaks=c("Faecalibacterium", "Ruminococcaceae MK121885.1",
                             "Subdoligranulum variabile", "Gemmiger formicilis",
                             " "),
                    values=c("#711A6EFF", "#B1325AFF","#F17020FF", "#F7D340FF",
                             "white"),
                    labels = c("*Faecalibacterium*",
                               "*Ruminococcaceae* MK121885.1",
                               "*Subdoligranulum variabile*",
                               "*Gemmiger formicilis*", " ")) +
  theme(legend.justification = "top", legend.text.align = 0,
        legend.text = element_markdown(size = 7))

p3 <- p2 + new_scale_fill()
p3 <- gheatmap(p3, tax2c, colnames = T, offset=.16, width=.18, font.size=2,
               colnames_angle=45, custom_column_labels = col_lab_2,
               colnames_offset_x = -.0, colnames_offset_y = 0.4, hjust=1) +
  scale_fill_manual(name = c("Comparison abundance per ethnicity"),
                    breaks=c("Dutch", "Other ethnicity"),
                    values=c("darkorange", "darkgreen")) +
  theme(legend.justification = "top",legend.text = element_markdown(size = 7))

p4 <- p3 + new_scale_fill()

p5 <- gheatmap(p4, taxx_3_c, colnames = T, offset=.19, width=.2, font.size=2,
               colnames_angle=45, custom_column_labels = col_lab_3,
               colnames_offset_x = -.0,colnames_offset_y = 0.4,hjust=1) +
  scale_fill_manual("Comparison abundance per generation     ",
                    breaks=c("First generation", "First generation",
                             "Second generation", "Second generation",
                             "First generation", "Second generation",
                             "First generation", "Second generation"),
                    values=c("blue","blue","brown","brown","blue", "brown",
                             "blue","brown","blue", "brown"))+
  theme(legend.justification = "top",legend.text = element_markdown(size = 7))

p6 <- p5 + theme(legend.position = "none")
```

```
p6 + vexpand(0.05, -1) + theme(legend.justification = c(1,0.8),
                               legend.key.size = unit(0.5, 'cm'),
                               title = element_text(size = 8))
```

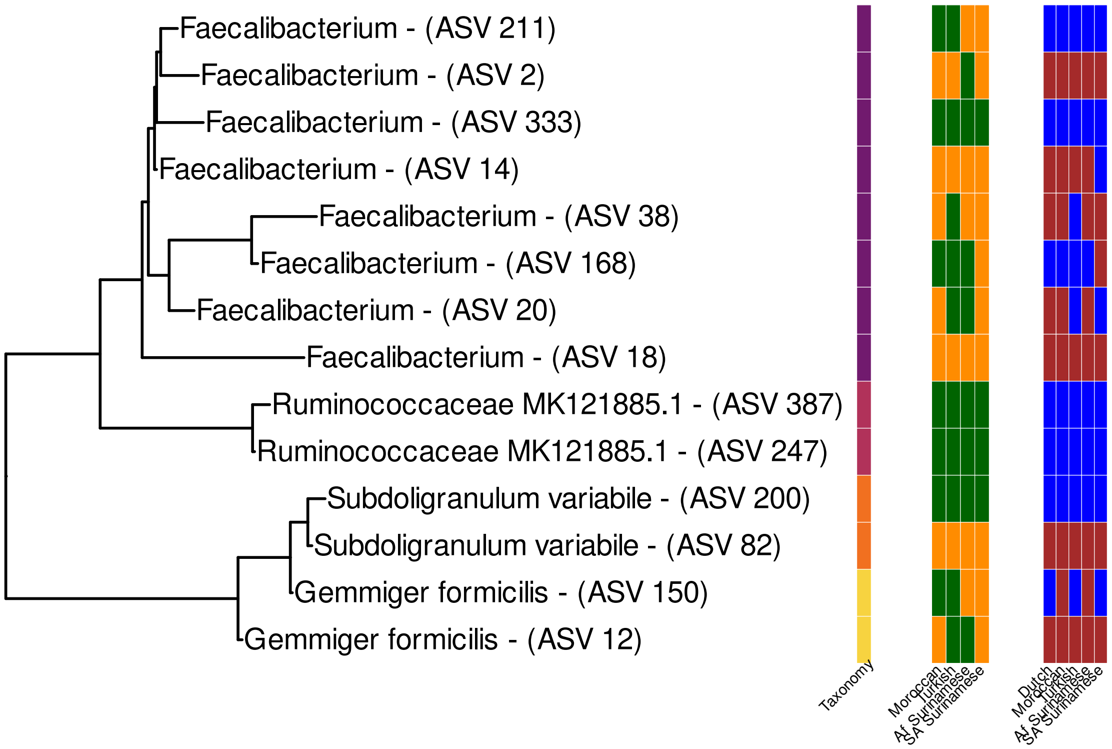


```
as_ggplot(get_legend(p5))
```

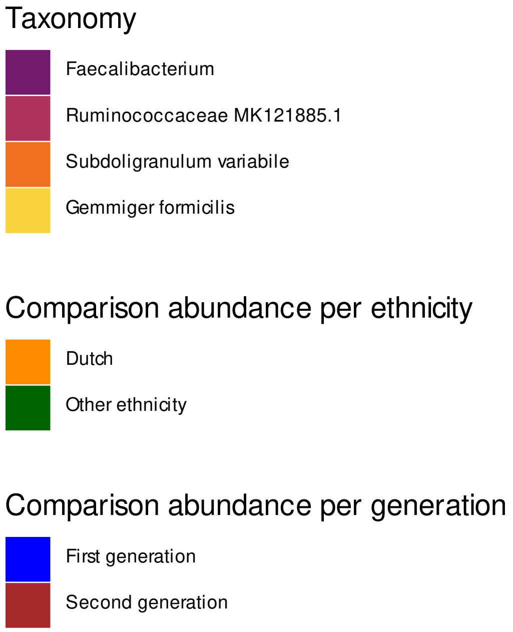

Correlations of clusters and trophic networks to alpha diversity (shannon effectiven number)

```
asv <- as.data.frame(otu_table(ps_h))
asv <- (asv/rowSums(asv))*100

names(asv) <- gsub("_"," ", sub("0+","", names(asv)))
alpha <- rio::import("Intermediate_files/All_alpha_diversity_metrics.xlsx")
pster <-
  rio::import("Intermediate_files/Supplementary_table_pster_ASVs.xlsx") %>%
  filter(Sum >= 6)
bacblau <-
  rio::import("Intermediate_files/Supplementary_table_BBB_ASVs.xlsx") %>%
  filter(Sum >= 6)
rcmo <-
  rio::import("Intermediate_files/Supplementary_table_RCMO_ASVs.xlsx") %>%
  filter(Sum >= 6)
pcopri <-
  rio::import("Intermediate_files/Supplementary_table_pcopri_ASVs.xlsx") %>%
  filter(Sum >= 6)
pster_only <- pster[!pster$ASV_ID %in% pcopri$ASV_ID,]



asv_pcopri <- asv[,names(asv) %in% pcopri$ASV_ID] %>% rowSums(.) %>%
  as.data.frame(.) %>% dplyr::rename("pcopri" = ".")
asv_pster_only <- asv[,names(asv) %in% pster_only$ASV_ID] %>% rowSums(.) %>%
  as.data.frame(.) %>% dplyr::rename("pster" = ".")
asv_prev <- merge(asv_pcopri, asv_pster_only, by="row.names")
rownames(asv_prev) <- asv_prev$Row.names
asv_prev$Row.names <- NULL

asv_pster <- asv[,names(asv) %in% pster$ASV_ID] %>% rowSums(.)%>%
  as.data.frame(.) %>% dplyr::rename("Prevotella" = ".")
asv_pster$clinparam <- alpha$Shannon_effective[match(rownames(asv_pster),
                                                     alpha$Heliusnr)]
asv_pster$etn_migrgen <- dat_h$etn_migrgen[match(rownames(asv_pster),
                                                 dat_h$Heliusnr)]
asv_pster <- merge(asv_pster, asv_prev, by = "row.names")
rownames(asv_pster) <- asv_pster$Row.names
asv_pster$Row.names <- NULL

asv_bbb <- asv[,names(asv) %in% bacblau$ASV_ID] %>% rowSums(.) %>%
  as.data.frame(.) %>% dplyr::rename("BBB" = ".")
asv_bbb$clinparam <- alpha$Shannon_effective[match(rownames(asv_bbb),
                                                   alpha$Heliusnr)]
asv_bbb$etn_migrgen <- dat_h$etn_migrgen[match(rownames(asv_bbb),
                                               dat_h$Heliusnr)]

asv_cmo <- asv[,names(asv) %in% rcmo$ASV_ID] %>% rowSums(.) %>%
  as.data.frame(.) %>% dplyr::rename("CMO" = ".")
asv_cmo$clinparam <- alpha$Shannon_effective[match(rownames(asv_cmo),
                                                   alpha$Heliusnr)]
asv_cmo$etn_migrgen <- dat_h$etn_migrgen[match(rownames(asv_cmo),
```

```
                                               dat_h$Heliusnr)]

asv_all <- asv_pster
asv_all$CMO <- asv_cmo$CMO[match(rownames(asv_all), rownames(asv_cmo))]
asv_all$BBB <- asv_bbb$BBB[match(rownames(asv_all), rownames(asv_bbb))]
asv_all <- asv_all %>% gather("group","value",1,4,5,6,7) %>% filter(
  group=="pcopri"|group=="pster"|group=="CMO"|group=="BBB")
asv_all$group <- as.factor(asv_all$group)
asv_all$group <- factor(asv_all$group,levels=c("pcopri","pster","CMO","BBB"))


asv_mo <- asv_all[asv_all$etn_migrgen=="Moroccan G1" |
                    asv_all$etn_migrgen=="Moroccan G2",]
asv_tu <- asv_all[asv_all$etn_migrgen=="Turkish G1" |
                    asv_all$etn_migrgen=="Turkish G2",]
asv_du <- asv_all[asv_all$etn_migrgen=="Dutch (Mean age = 57.2)" |
                    asv_all$etn_migrgen=="Dutch (Mean age = 32.4)",]
asv_as <- asv_all[asv_all$etn_migrgen=="Af Surinamese G1" |
                    asv_all$etn_migrgen=="Af Surinamese G2",]
asv_sasu <- asv_all[asv_all$etn_migrgen=="SA Surinamese G1" |
                      asv_all$etn_migrgen=="SA Surinamese G2",]

asv_list <- list(asv_mo, asv_tu, asv_du, asv_as, asv_sasu)
group_list <- list("Moroccan", "Turkish", "Dutch", "African Surinamese",
                   "South-Asian Surinamese")
plot_list <- vector(mode = 'list', length = length(asv_list))
cor_list <- plot_list

for (i in 1:length(asv_list)){
plot_list[[i]] <- ggscatter(
  asv_list[[i]], x = "value", y = "clinparam", color = "group",
  add = "reg.line", conf.int = TRUE,
  shape = 20,
  xlab = "", ylab = "",
  title = group_list[[i]],
  alpha=0.4, size=2,stroke=0
) +
  stat_cor(aes(color = group,
               label = paste(..r.label..,
                             cut(..p.., breaks = c(-Inf, 0.001, 0.01, 0.05, Inf),
                                 labels = c("'***'", "'**'", "'*'", "' '")),
                                            sep = "~")), method = "spearman",
           label.y = c(140,130,120,110), label.x = 40, cor.coef.name = "rho") +
  scale_color_manual(
    name = 'Group',
    labels = c(expression(italic("P. copri")),
               expression(italic("P. stercorea")~"trophic network"),
               "CMO trophic network", "BBB cluster"),
    values=c("darkred","darkgreen","darkorange","darkblue")
  ) +
  scale_fill_manual(
    name = 'Group',
    labels = c(expression(italic("P. copri")),
```

```
               expression(italic("P. stercorea")~"trophic network"),
               "CMO trophic network", "BBB cluster"),
    values=c("darkred","darkgreen","darkorange","darkblue")
  ) +
  scale_y_continuous(limits = c(0, 220)) +
  scale_x_continuous(breaks = seq(0,80, len=4.5), limits = c(0, 80)) +
  theme(plot.title = element_text(hjust = 0.5),
        legend.position=c(0.7,0.85),
        legend.text.align = 0)
}

plot_list[[1]]
```

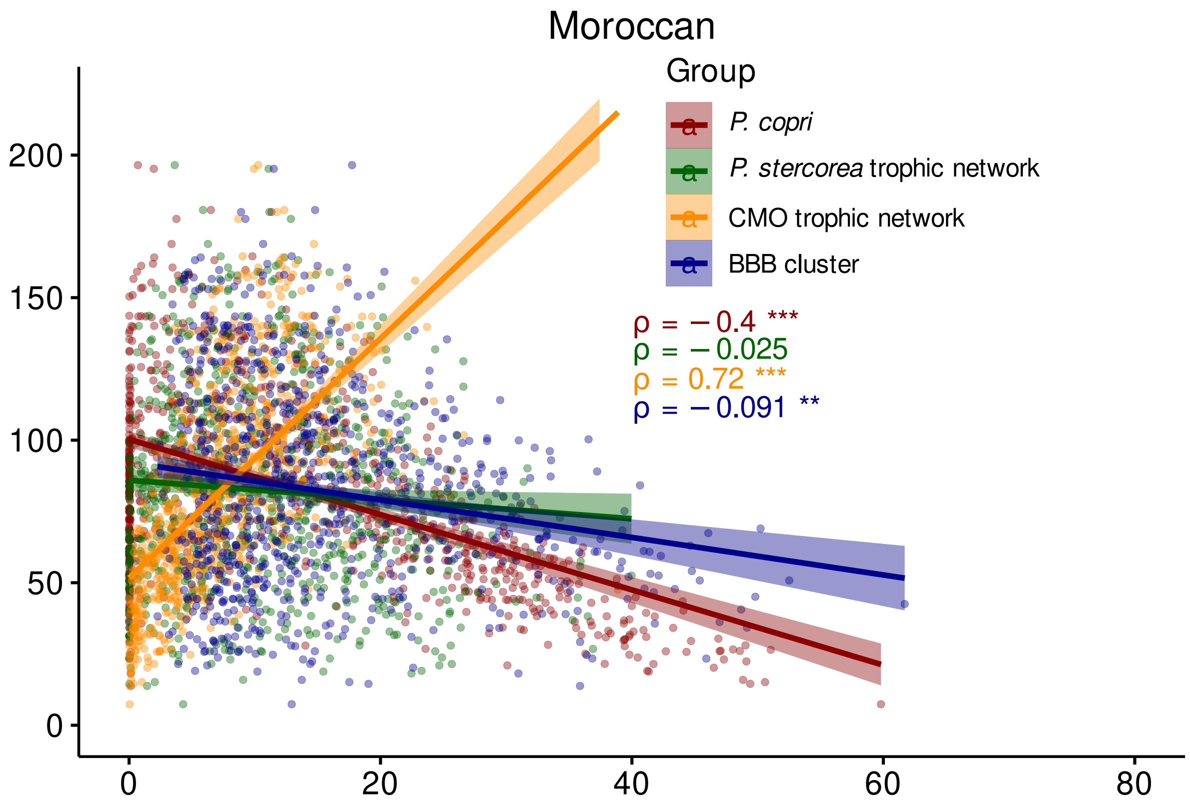


```
plot_list[[2]]
```

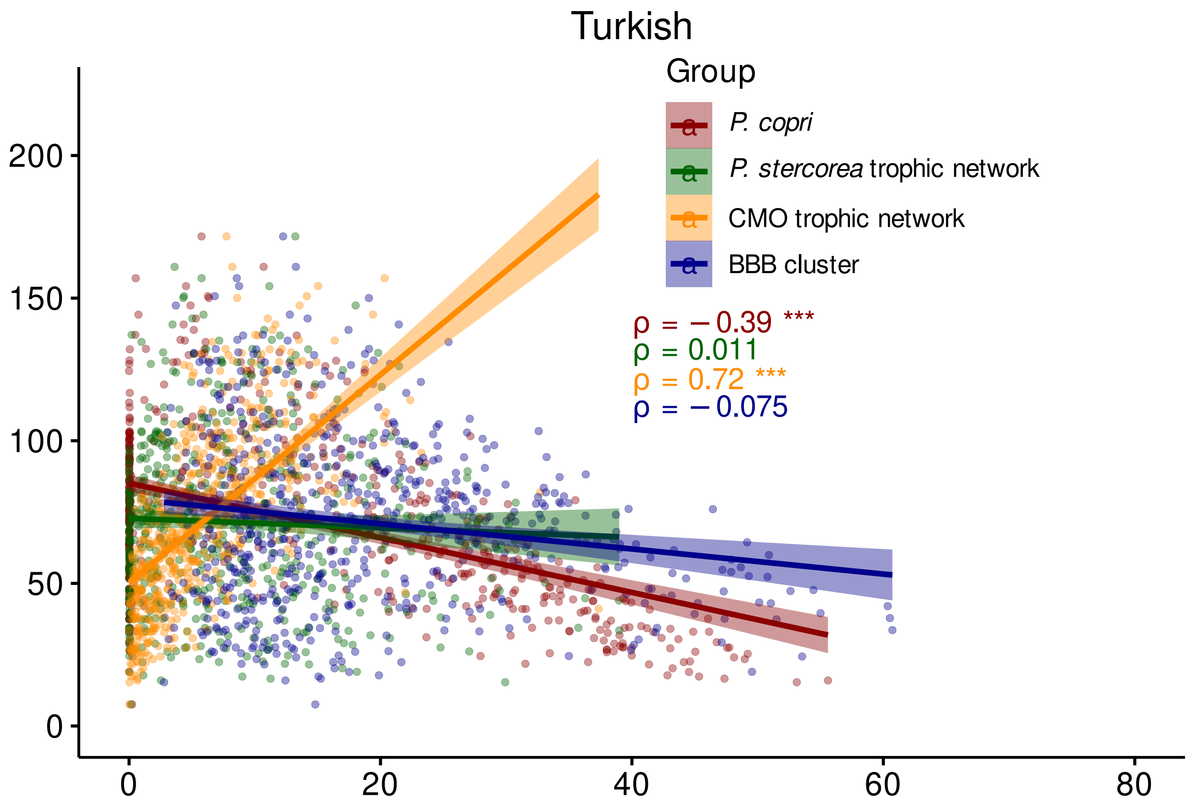
Turkish
Group
P. copri
P. stercorea trophic network
CMO trophic network
BBB cluster
ρ = −0.39 ***
ρ = 0.011
ρ = 0.72 ***
ρ = −0.075
200
150
100
50
0
0
20
40
60
80

```
plot_list[[3]]
```

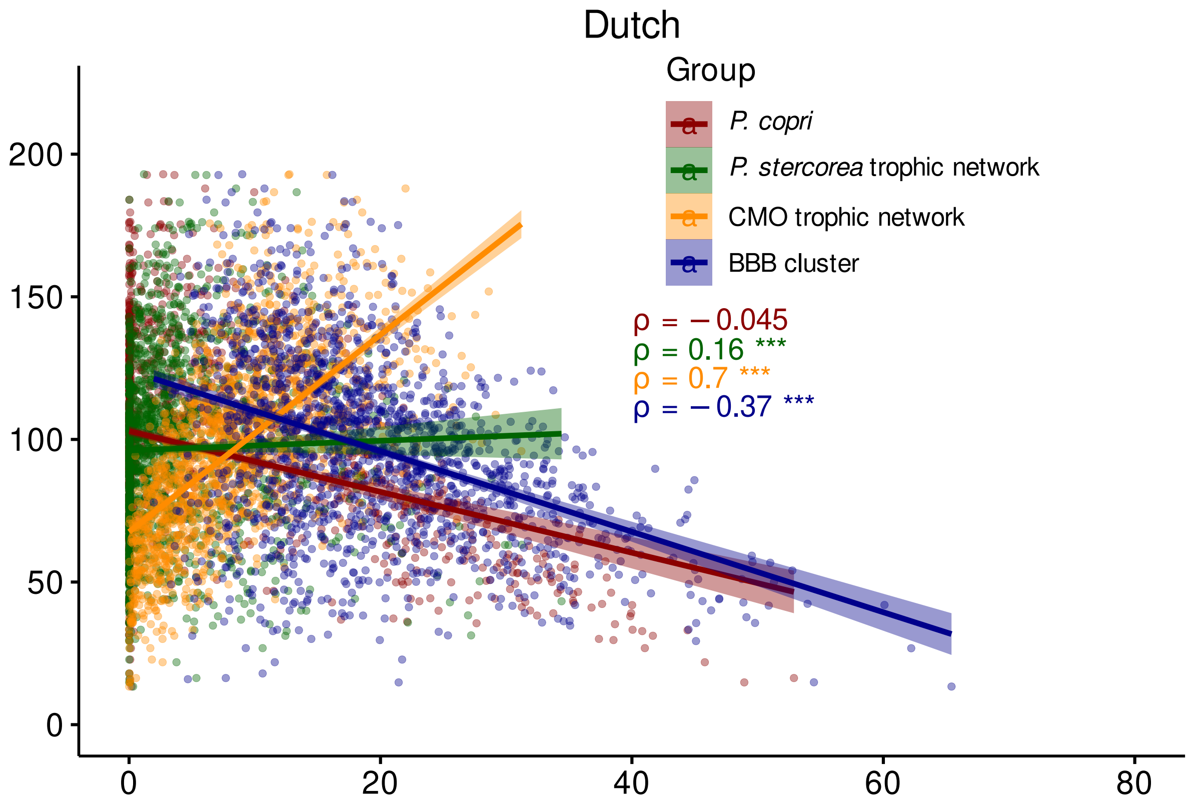
Dutch
Group
P. copri
P. stercorea trophic network
CMO trophic network
BBB cluster
ρ = −0.045
ρ = 0.16 ***
ρ = 0.7 ***
ρ = −0.37 ***
200
150
100
50
0
0
20
40
60
80

```
plot_list[[4]]
```

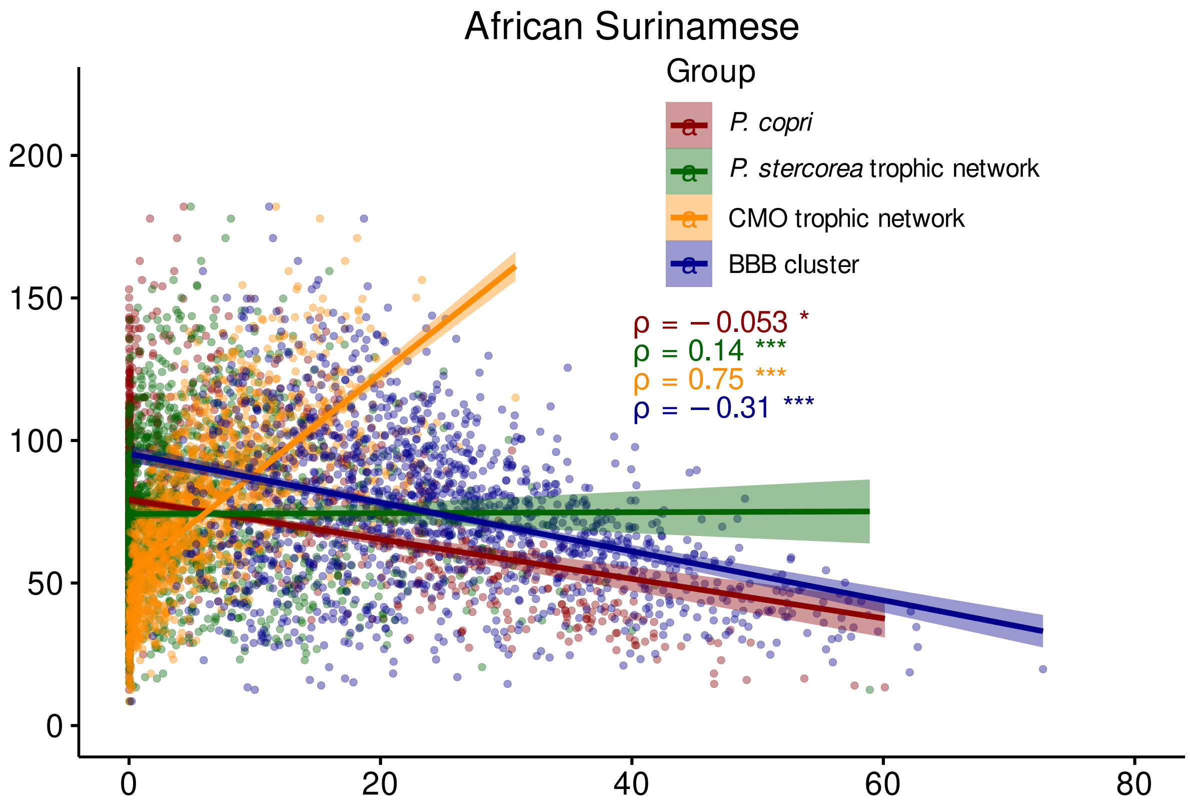


```
plot_list[[5]]
```

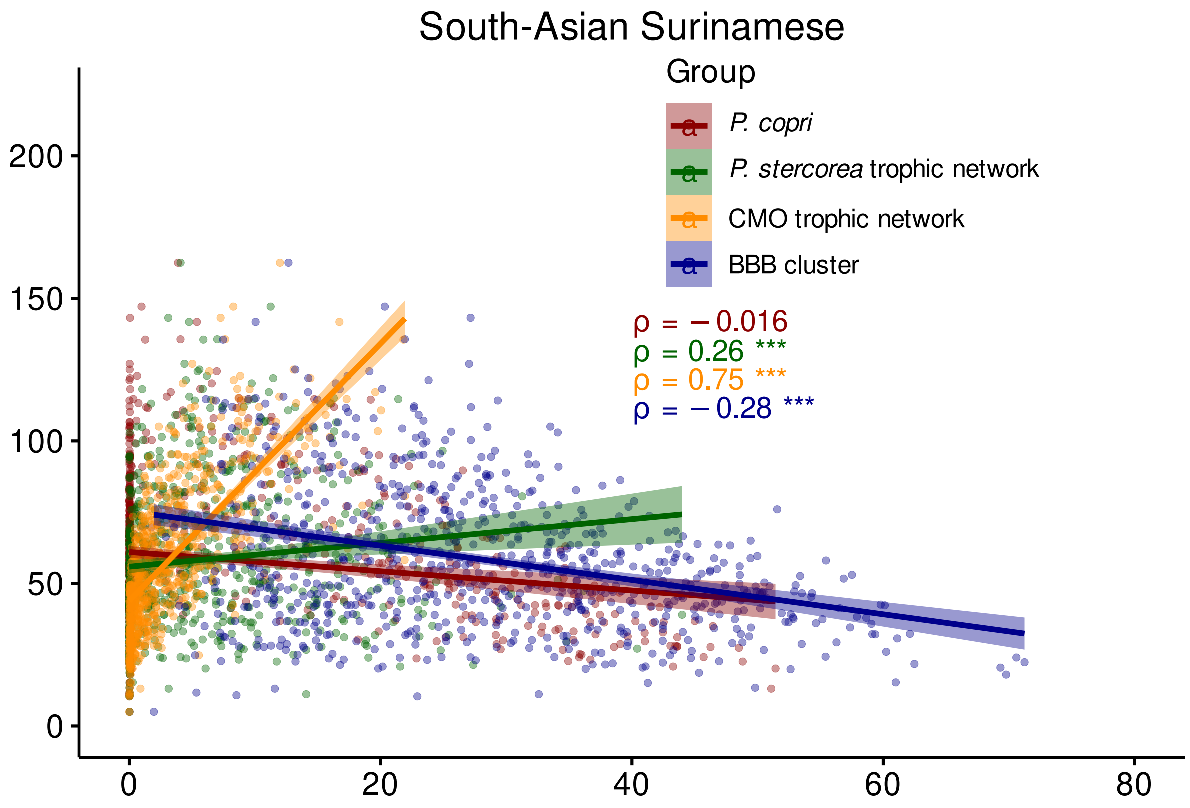

Heatmap used in the main figure

The heatmap used as figure 5 was based on the Turkish first generation.

```
asv_h <- as.data.frame(ps_h@otu_table)
ytrain_tu <- rio::import("ML_results/tu/Y_train_TU.xlsx")
xtrain_tu <- rio::import("ML_results/tu/X_train_TU.xlsx")
tu <- rio::import("ML_results/tu/feat_imp_gini.txt")
dat_tu1 <- dat_h[dat_h$etn_migrgen=="Turkish G1",]
turk_asv <- rio::import("Intermediate_files/cor_matrix_Turkish_G1.xlsx") %>%
  column_to_rownames("...1")
stables_hm <- rio::import(
  "Intermediate_files/All_Blasted_ASVs_heatmap_names.xlsx") %>%
   mutate(ASV_comb = paste0(.$ASV_name, " - (", .$ASV_ID,")"))

tu <- tu[order(as.numeric(-tu$RelFeatImp)),] %>%
  mutate(ASV_ID = gsub("_"," ", sub("0+","", .$FeatName))) %>%
  mutate(names_full = stables_hm$ASV_comb[match(.$ASV_ID,
                                                 stables_hm$ASV_ID)])

x_tu1 <- asv_h[rownames(asv_h) %in% dat_tu1$Heliusnr,] %>%
  .[,order(colSums(-., na.rm=TRUE))] %>%
  select(1:200)
x_tu1_hm <- asv_h[rownames(asv_h) %in% ytrain_tu$SAMPLE_ID,
                  names(asv_h) %in% names(x_tu1)] %>%
  .[,order(colSums(-.,na.rm=TRUE))]
x_tu1_hm <- x_tu1_hm[,order(colSums(-x_tu1_hm,na.rm=TRUE))]

names(x_tu1) <- stables_hm$ASV_comb[match(gsub("_"," ", sub("0+","",
                                                             names(x_tu1))),
                                          stables_hm$ASV_ID)]

turk_asv1 <- x_tu1[,names(x_tu1) %in% names(turk_asv)]


labs_tu1 <- ASV_labels_heatmap(xtrain = x_tu1_hm, ytrain = ytrain_tu)
labs_tu1$featname <- stables_hm$ASV_comb[match(
  gsub("_"," ", sub("0+","",labs_tu1$featname)), stables_hm$ASV_ID)]
```

note that the heatmap will not be visually pleasing as the plot area is too small in RMarkdown

```
heatmap_mainfig <- heatmap_main(x = turk_asv, toplist = tu, labs = labs_tu1)
```

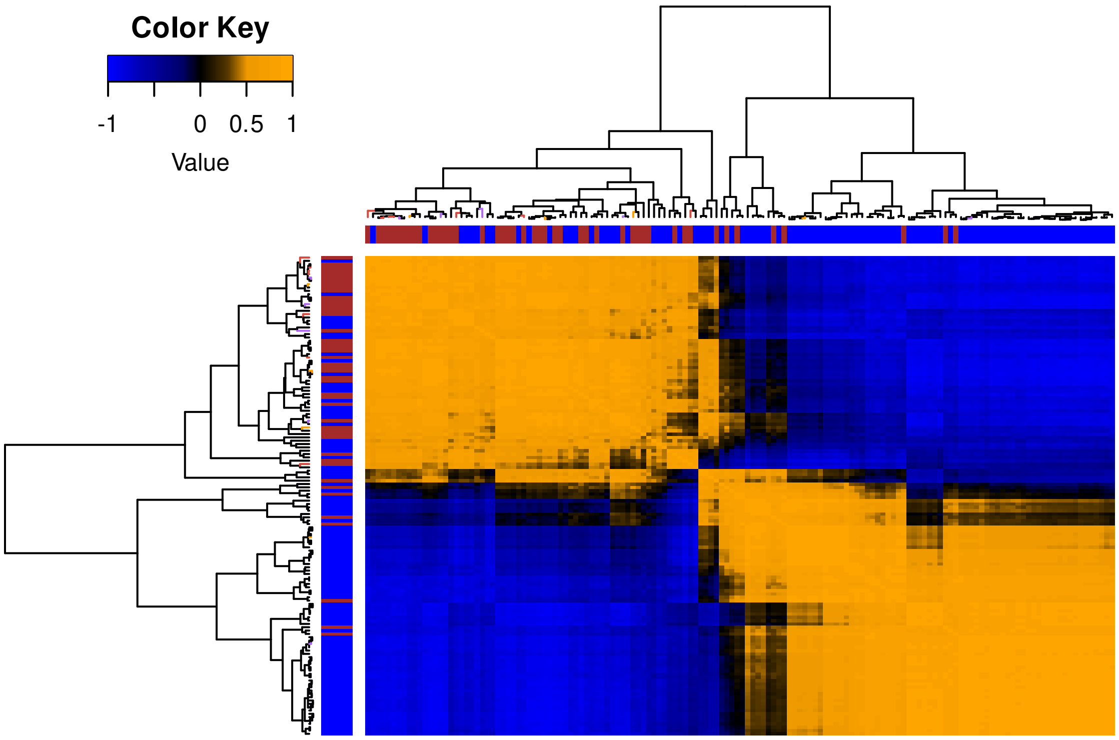
Color Key
-1
0
0.5
1
Value
